# Design, Synthesis, and Herbicidal Activity of Novel Dihydrochalcones Derived from Flavokawains and Their Analogs

**DOI:** 10.3390/molecules31101587

**Published:** 2026-05-09

**Authors:** Suriyaphong Poprom, Jatuporn Meesin, Warot Chotpatiwetchkul, Watcharee Waratchareeyakul, Nawasit Chotsaeng, Chamroon Laosinwattana, Naphat Somala

**Affiliations:** 1 Advanced Pure and Applied Chemistry Research Unit, Department of Chemistry, School of Science, King Mongkut’s Institute of Technology Ladkrabang, Bangkok 10520, Thailand; 67056084@kmitl.ac.th (S.P.); jatuporn.me@kmitl.ac.th (J.M.); 2 Applied Computational Chemistry Research Unit, Department of Chemistry, School of Science, King Mongkut’s Institute of Technology Ladkrabang, Bangkok 10520, Thailand; warot.ch@kmitl.ac.th; 3 Department of Chemistry, Faculty of Science and Technology, Rambhai Barni Rajabhat University, Chanthaburi 22000, Thailand; watcharee.w@rbru.ac.th; 4 Faculty of Health, Science, Social Care and Education, Kingston University, London KT1 2EE, UK; 5 Department of Plant Production Technology, School of Agricultural Technology, King Mongkut’s Institute of Technology Ladkrabang, Bangkok 10520, Thailand; chamroon.la@kmitl.ac.th (C.L.); naphat.so@kmitl.ac.th (N.S.)

**Keywords:** herbicidal, dihydrochalcones, flavokawains, semisynthetic herbicides, alkyl aryl ketones, mode of action

## Abstract

Weeds are a major cause of crop production losses worldwide, and environmentally friendly chemical control based on natural or semisynthetic compounds has attracted increasing attention. In this study, flavokawains, a class of natural chalcones, and their analogues were converted into dihydrochalcones (**1**–**27**) via Pd-catalyzed hydrogenation. The herbicidal activities of these compounds were evaluated against Chinese amaranth (*Amaranthus tricolor*) and barnyard grass (*Echinochloa crus-galli*). Several compounds significantly inhibited seed germination and seedling growth in both species. Herbicidal activity was strongly influenced by the type and position of aromatic substituents, with electron-withdrawing groups and *meta* substitution providing higher activity. The *meta*-chloro derivative (**15**) exhibited the highest activity, markedly inhibiting seed germination as well as shoot and root growth. Further investigation of its mode of action revealed that this compound interfered with seed imbibition, inhibited α-amylase activity, and affected membrane integrity and malondialdehyde (MDA) levels in *A. tricolor* in a concentration-dependent manner. These findings provide valuable insights for the development of natural product-derived herbicides.

## 1. Introduction

Since weeds are one of the leading causes of crop production losses worldwide, agrochemists have developed several approaches to weed control [1,2,3,4,5,6]. Among these, chemical control—particularly the use of synthetic herbicides—has been widely adopted because it is efficient, effective, and relatively inexpensive [7,8,9]. However, the long-term use of synthetic herbicides poses significant challenges, as their prolonged residual toxicity, slow degradation rates, and contribution to the development of herbicide-resistant weeds can harm both farmers and the environment [3,8,10,11,12,13,14,15]. These concerns have prompted farmers and agrochemists to seek alternative weed-control strategies, although chemical control remains a viable area for further development, and the synthesis of novel herbicides continues to be challenging [16,17,18,19,20,21].

In addition to the direct use of natural products or natural extracts for weed control, the semisynthetic modification of these compounds has recently gained increasing attention [3,21,22,23,24,25,26,27,28,29,30]. Natural substances and their semisynthetic derivatives are generally believed to degrade more rapidly and exhibit lower environmental toxicity [31,32,33,34,35,36,37,38]. Moreover, several studies have demonstrated that structural modification of certain natural products can lead to herbicides with enhanced biological activity [19,23,28,29,30,39,40,41].

**Figure 1:**
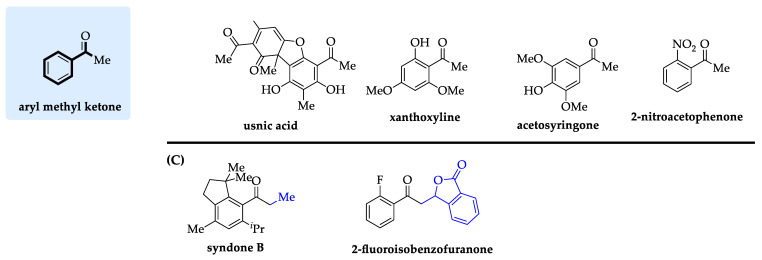
Chemical structures of aryl methyl ketone (**A**), aryl methyl ketone–based herbicides (**B**), and alkyl aryl ketone herbicides (**C**).

Aryl methyl ketones (ArCOMe) are versatile and cost-effective organic compounds that serve as key precursors for the synthesis of aromatic heterocycles and other related compounds (Figure 1) [42,43]. In addition, several reports have shown that various aryl methyl ketone derivatives (Figure 1B)—including natural products such as usnic acid, xanthoxyline, and acetosyringone, as well as synthetic compounds such as 2-nitroacetophenone—exhibit promising herbicidal activity against tested plant species [44,45,46,47]. Although the mechanisms of action of most of these compounds have not been fully elucidated, our previous research demonstrated that xanthoxyline reduces seed imbibition and inhibits α-amylase activity in tested plants [46]. Furthermore, molecular docking studies on α-amylase (PDB ID: 1BG9) and 4-hydroxyphenylpyruvate dioxygenase (HPPD) suggest that carbonyl and aromatic moieties play key roles in enzyme–inhibitor interactions [44,46].

Beyond aryl methyl ketones, certain alkyl aryl ketones (Figure 1C), in which the methyl group is replaced with bulkier substituents (e.g., syndone B and 2-fluoroisobenzofuranone), have also been investigated as herbicidal agents [48,49]. These compounds have demonstrated strong inhibitory effects on plant growth and development.

**Scheme 1:**
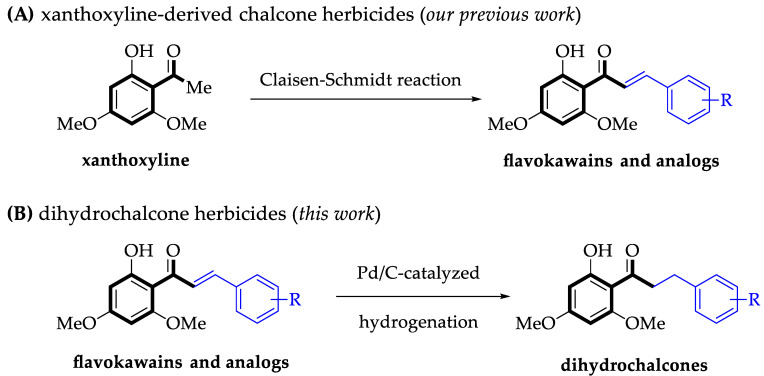
Synthetic routes to xanthoxyline-derived chalcone herbicides from our previous work (**A**) and to dihydrochalcone herbicides investigated in the present study (**B**).

As discussed above, extension of the methyl side chain of aryl methyl ketones to bulkier substituents can result in active herbicidal compounds. Therefore, in this study, we focused on transforming the natural herbicide xanthoxyline into dihydrochalcone derivatives (Scheme 1). In our previous work, xanthoxyline and other aryl methyl ketones were converted into flavokawains and chalcone derivatives, which were subsequently evaluated for their herbicidal properties (Scheme 1A) [50]. Several of these compounds exhibited promising inhibitory activity against plant growth. The herbicidal activity of chalcones is strongly influenced by the type and position of substituents on their aromatic rings. To further investigate this structure–activity relationship, flavokawains and xanthoxyline-derived chalcones were hydrogenated to yield dihydrochalcones via catalytic hydrogenation using palladium on carbon as the catalyst (Scheme 1B) [51]. The resulting dihydrochalcones were then evaluated for their herbicidal activity against plant growth and development. The most active compound was further investigated to elucidate its mode of action. The results of this study are expected to contribute to the development of new natural product-derived herbicides.

## 2. Results and Discussions

### 2.1. Synthesis of Dihydrochalcones (***1***–***27***)

**Scheme 2:**
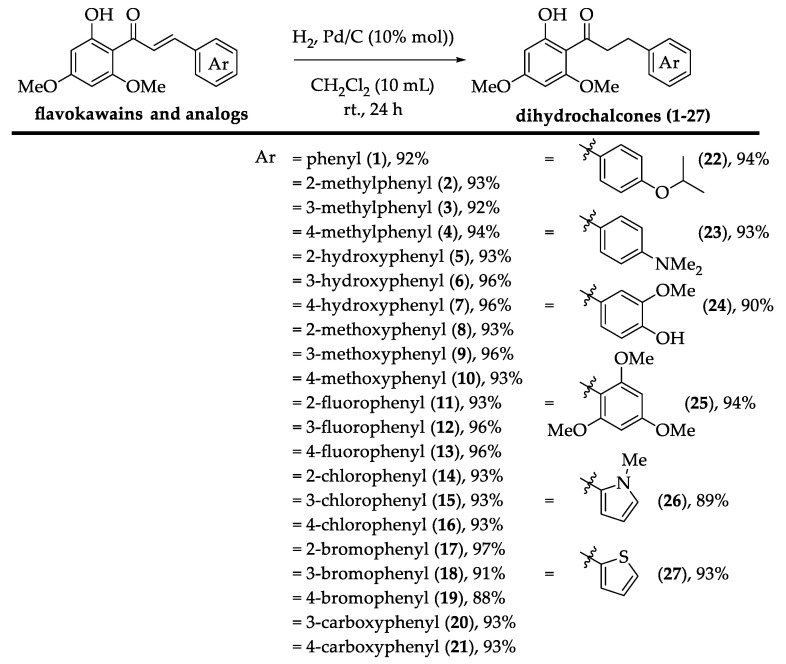
Synthesis of flavokawain-derived dihydrochalcones (**1**–**27**), and their isolated yields.

In this study, flavokawains and their chalcone derivatives bearing electron-donating and electron-withdrawing substituents, as well as heterocyclic moieties, were used as starting materials for hydrogenation to produce dihydrochalcones. The chalcone precursors were synthesized according to our previously reported procedures [50,52]. Subsequently, following the method reported by Sota and co-workers, the chalcones were reduced via Pd-catalyzed hydrogenation to afford the corresponding dihydrochalcones (**1**–**27**) in high yields (88–97%) (Scheme 2) [51]. The hydrogenation selectively reduced the C=C bond of the α,β-unsaturated ketone moiety while leaving the carbonyl group intact. This selectivity was confirmed by the disappearance of the olefinic proton signals in the ^1^H NMR spectra and the retention of the characteristic carbonyl stretching band in the IR spectra.

### 2.2. Herbicidal Activity of Dihydrochalcones (***1***–***27***)

**Figure 2:**
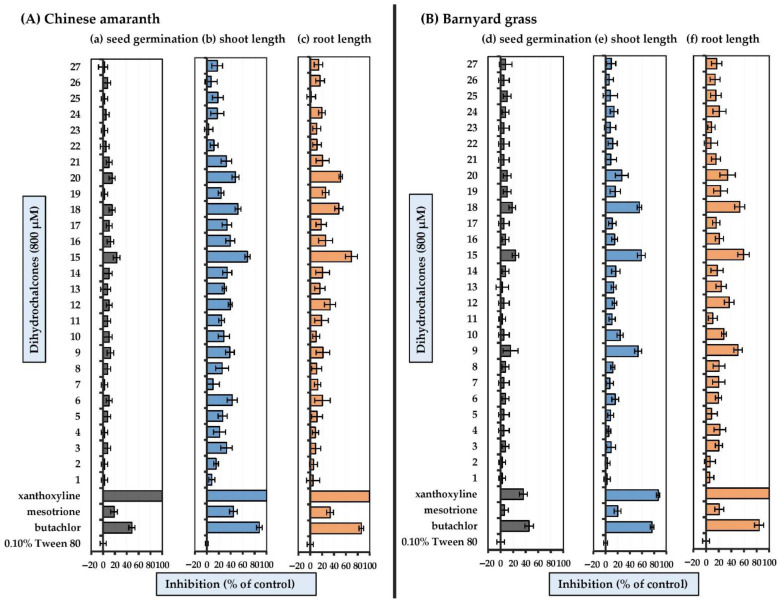
Herbicidal activity of dihydrochalcones (**1**–**27**) at 800 µM on seed germination, shoot growth, and root growth of Chinese amaranth (**A**) and barnyard grass (**B**). An aqueous solution of Tween 80 was used as the negative control. Butachlor, mesotrione, and xanthoxyline at 800 µM were used as positive controls. Horizontal bars represent the standard error of the mean from four replicates.

All synthesized dihydrochalcone derivatives (**1**–**27**) were initially evaluated at a concentration of 800 µM to assess their effects on seed germination and early seedling development of Chinese amaranth (*Amaranthus tricolor*) and barnyard grass (*Echinochloa crus-galli*), representing dicotyledonous and monocotyledonous plants, respectively (Figure 2). Commercial herbicides butachlor and the ketone herbicide mesotrione, together with the natural ketone xanthoxyline, were included as positive controls, whereas a 0.1% (*v*/*v*) aqueous solution of Tween 80 was used as the negative control.

For Chinese amaranth, most dihydrochalcones showed no inhibitory effect on seed germination; only 3-methoxydihydrochalcone (**9**), 3-chlorodihydrochalcone (**15**), 3-bromodihydrochalcone (**18**), and 3-carboxydihydrochalcone (**20**) caused a slight reduction (Figure 2A). In contrast, pronounced effects were observed on shoot and root growth, indicating that herbicidal activity is strongly influenced by the nature and position of aromatic substituents. Dihydrochalcones bearing electron-withdrawing groups generally exhibited stronger inhibition than those with electron-donating groups. For shoot elongation, inhibitory activity followed the order Cl > Br ≈ COOH > OH > F ≈ OMe > Me, with *meta*-substituted derivatives being more active than *para*- or *ortho*-substituted analogues. At 800 µM, compound **15** was the most active, inhibiting shoot growth by 68%.

A similar substituent-dependent trend was observed for root growth inhibition (Figure 2A), with activity decreasing in the order Cl > Br ≈ COOH > F. Compound **15** again showed the strongest effect, suppressing root elongation by 70% at 800 µM. Overall, although less active than xanthoxyline and butachlor, compound **15** exhibited stronger phytotoxicity than mesotrione.

For barnyard grass (Figure 2B), most of the dihydrochalcones did not affect seed germination, and only weak inhibition was observed for derivatives **9**, **15**, and **18**. As observed for Chinese amaranth, electron-withdrawing substituents generally enhanced shoot and root growth inhibition. Shoot growth inhibition followed the order Cl ≈ Br ≈ OMe > COOH, while root inhibition decreased in the order Cl > Br ≈ OMe > F ≈ COOH. Among the tested compounds, 3-chlorodihydrochalcone (**15**) was the most active, inhibiting root and shoot elongation by 59% and 53%, respectively. Although this compound was markedly less active than xanthoxyline and butachlor, it exhibited greater activity than mesotrione.

### 2.3. Dose Responses of the Most Active Dihydrochalcones

**Figure 3:**
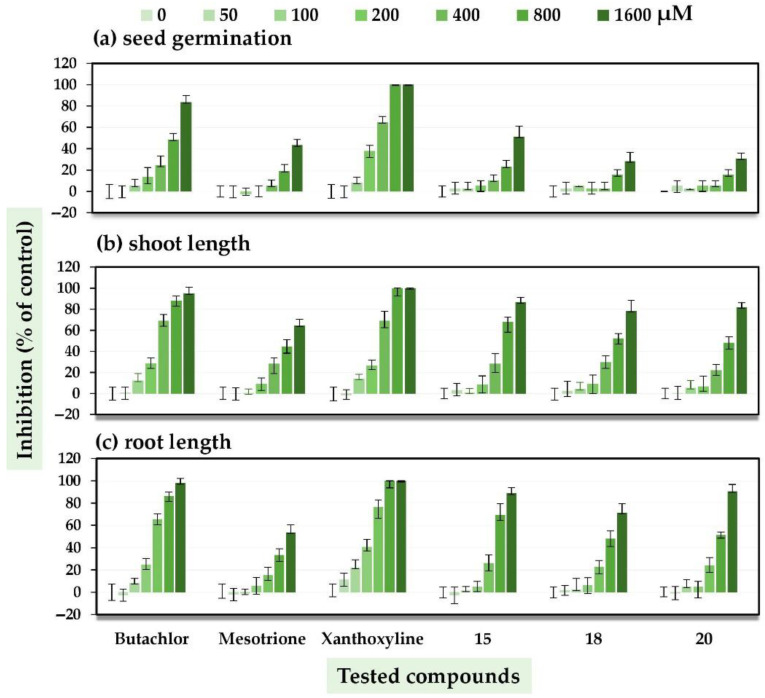
Dose–response effects of dihydrochalcones **15**, **18**, and **20** (50–1600 μM) on seed germination (**a**), shoot growth (**b**), and root growth (**c**) of Chinese amaranth (*A. tricolor*). Butachlor, mesotrione, and xanthoxyline were used as positive controls, while Tween 80 served as the negative control (0 μM in the graph). Error bars represent the standard error of the mean based on four replicates.

**Figure 4:**
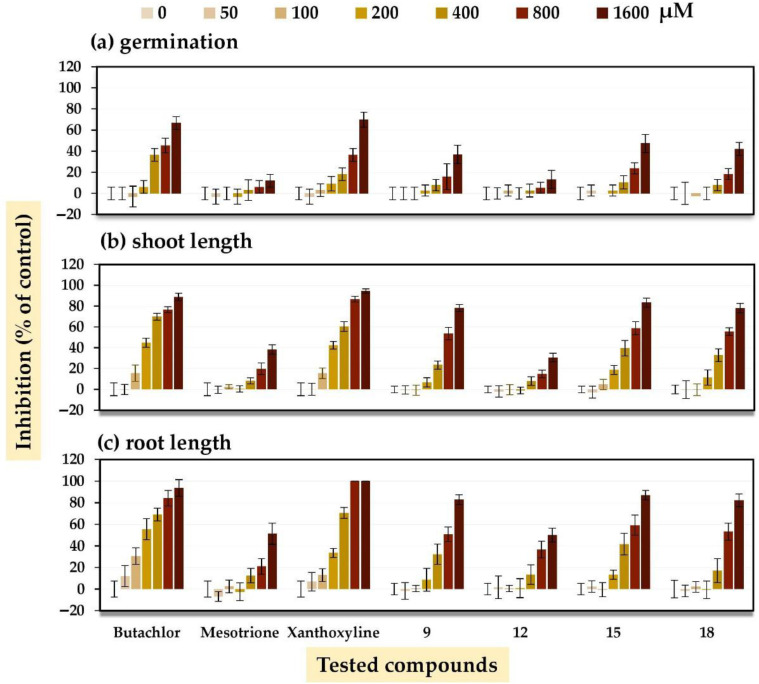
Dose–response effects of dihydrochalcones **15**, **18**, and **20** (50–1600 μM) on seed germination (**a**), shoot growth (**b**), and root growth (**c**) of barnyard grass (*E. crus-galli*). Butachlor, mesotrione, and xanthoxyline were used as positive controls, while Tween 80 served as the negative control (0 μM in the graph). Error bars represent the standard error of the mean based on four replicates.

The above results revealed the most effective dihydrochalcones against plant growth. In general, at 800 µM, dihydrochalcones inhibited plant growth only to a low-to-moderate extent; therefore, only a few selected dihydrochalcones were further investigated to evaluate their dose–response relationships using the procedures as our previously reported [44,46,49]. The tested concentrations ranged from 50 to 1600 μM, and butachlor, mesotrione, and xanthoxyline were used as positive controls (Figure 3 and Figure 4).

For Chinese amaranth, compounds **15**, **18**, and **20** were selected for further evaluation (Figure 3). At lower concentrations, these compounds did not inhibit seed germination. At higher concentrations, they exhibited moderate inhibition of seed germination, with 3-chlorodihydrochalcone (**15**) showing the greatest effect, reducing germination by 51% at 1600 μM.

A similar trend was observed for shoot and root growth inhibition (Figure 3). The order of inhibitory activity was compounds **15** > **20** > **18**, and the level of inhibition increased with increasing concentration. At 1600 μM, compound **15** inhibited shoot and root elongation by 87% and 89%, respectively. Compared with the positive controls, all tested compounds were less active than xanthoxyline and butachlor but slightly more active than mesotrione in terms of seedling growth inhibition.

In the case of barnyard grass, four compounds (**9**, **12**, **15**, and **18**) were further investigated, and they inhibited seed germination only at high applied concentrations (Figure 4). The trends observed for shoot and root growth inhibition were similar to those observed for Chinese amaranth. The order of inhibitory activity was **15** ≈ **18** > **9** > **12**, and the degree of inhibition increased with increasing concentration. Compounds **9**, **15**, and **18** exhibited stronger inhibitory effects than the commercial herbicide mesotrione but were less active than the natural product xanthoxyline.

### 2.4. Study Mode of Action

**Figure 5:**
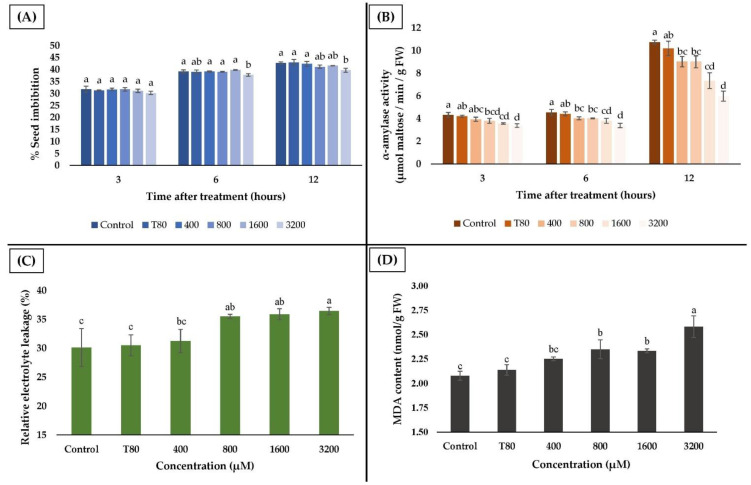
Effect of 3-chlorodihydrochalcone (**15**) on seed imbibition (**A**), α-amylase activity (**B**), membrane electrolyte leakage (**C**), and malondialdehyde (MDA) content (**D**) in *A. tricolor* seeds at 12 h after treatment. Tween 80 solution (T80, 3200 μM) was used as the solvent control, while deionized water (Control) served as the negative control. Error bars represent the standard deviation of four replicates. Means with different letters are significantly different as indicated by Tukey’s test (*p*  ≤  0.05).

Among the synthesized compounds, 3-chlorodihydrochalcone (**15**) exhibited the strongest inhibitory effects on seed germination and seedling growth in both test species. Accordingly, its mode of action was further investigated using Chinese amaranth as the model plant. The impacts on seed imbibition, α-amylase activity, membrane stability, and malondialdehyde (MDA) levels are described in the following section (Figure 5).

#### 2.4.1. Seed Imbibition and α-Amylase Activity

Seed imbibition, defined as the uptake of water by dry seeds, marks the first stage of germination. The influence of 3-chlorodihydrochalcone **15** on the imbibition behavior of *A. tricolor* seeds is shown in Figure 5A. As the soaking duration increased from 3 to 12 h, control treatments (distilled water and Tween 80) exhibited a steady rise in water absorption. In contrast, seeds exposed to compound **15** displayed a marked reduction in water uptake.

During the early phase (3–6 h), seeds in the water control demonstrated the greatest level of imbibition. Conversely, treatment with the highest concentration of compound **15** (3200 µM) resulted in the lowest imbibition percentage. This reduction may be associated with damage to cellular membranes, leading to impaired membrane integrity and increased permeability. Under normal conditions, the seed coat functions as a selectively permeable barrier that protects internal tissues from external stress; thus, disruption of this barrier can significantly hinder the germination process [53].

Figure 5B illustrates the effect of compound **15** on α-amylase activity and carbohydrate mobilization in seeds. Following imbibition, catabolic processes are initiated to support seed germination. α-Amylase is the key enzyme involved in starch mobilization, and its activity is essential for successful germination [54]. During germination, α-amylase hydrolyzes starch into smaller soluble sugars, providing the energy and nutrients required for early growth.

The activity of α-amylase was measured at 3, 6, and 12 h after treatment (Figure 5B). In general, α-amylase activity increased over time in all samples. At 3 h, no significant difference was observed between the treated seeds and the control. However, after 6 and 12 h, α-amylase activity decreased significantly depending on the concentration of the treatment solution. After 12 h, a clear dose-dependent effect was observed, with enzyme activity declining as the concentration increased. The lowest activity was detected at the highest concentration (3200 µM). The inhibition of α-amylase may limit starch hydrolysis and reduce the availability of soluble sugars required for growth and development, thereby contributing to the suppression of seed germination and seedling growth.

#### 2.4.2. Membrane Integrity

The effect of 3-chlorodihydrochalcone (**15**) on seed membrane integrity was evaluated by measuring relative electrolyte leakage (REL). The percentage of REL is an indicator of membrane permeability, as increased electrical conductivity reflects damage to membrane integrity [55]. Disruption of the plant plasma membrane can lead to cell death because the membrane plays a critical role in maintaining cellular structure and function [56].

Figure 5C shows the effect of compound **15** on membrane electrolyte leakage in *A. tricolor* seeds at 12 h after treatment. Overall, the percentage of REL increased with increasing concentrations of the compound, with the highest value (36.43%) observed at 3200 µM. An elevated REL indicates membrane damage and loss of membrane integrity. This leakage may also result from the excessive production and accumulation of reactive oxygen species (ROS) [57].

Changes in membrane permeability can affect biochemical and physiological processes associated with membrane function [58]. The impairment of membrane integrity disrupts cellular organization and metabolic activities, ultimately leading to inhibited plant growth and eventual cell death [59].

#### 2.4.3. Malondialdehyde (MDA) Content

Malondialdehyde (MDA) is a secondary product of the lipid peroxidation of polyunsaturated fatty acids and is widely used as an indicator of oxidative damage to plant cell membranes [57,60]. The MDA content in treated seeds was affected by dihydrochalcone **15** in a concentration-dependent manner (Figure 5D), with the highest value reaching 2.58 nmol/g FW.

The accumulation of MDA is associated with membrane damage and electrolyte leakage, as discussed above. Fatty acids, which are major components of plant cell membranes, are susceptible to oxidative degradation, leading to the formation of lipid peroxidation products. Elevated levels of reactive oxygen species (ROS) can enhance lipid peroxidation, which is reflected by increased MDA content. The observed increase in MDA levels suggests that treatment with compound **15** induced oxidative stress and lipid peroxidation, ultimately resulting in membrane disruption [61,62].

**Figure 6:**
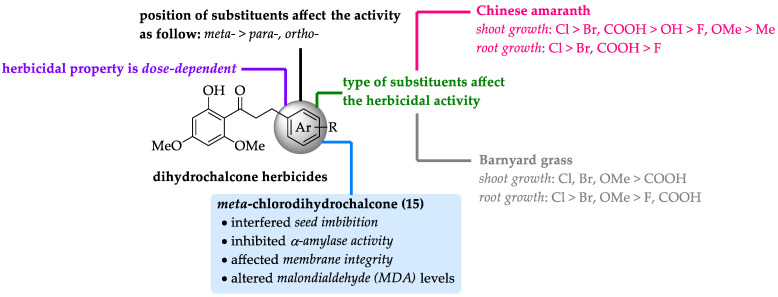
Summary of the herbicidal activities of the synthesized dihydrochalcones.

The above results indicated that the synthesized dihydrochalcones significantly affected plant germination and growth. The herbicidal activity of these compounds was strongly influenced by the type and position of aromatic substituents, with electron-withdrawing groups and *meta* substitution providing higher activity than other substituents, as summarized in Figure 6. In general, the herbicidal properties of organic compounds are influenced by the type and position of substituents [63,64,65,66,67,68,69,70,71]. Similarly, our previous reports demonstrated that the herbicidal activities of aromatic aldehydes, chalcones, and methyl ketones depend on the nature of the substituents [44,50,72]. Moreover, in our study on isobenzofuranone derivatives, we found that not only the type but also the position of substituents was crucial for activity [49]. Isobenzofuranones bearing *ortho*-substituted groups were more reactive than those with *meta*- or *para*-substituted groups.

In addition, the results revealed that the weed control ability of the novel dihydrochalcones was dose-dependent. As expected for herbicides, higher concentrations resulted in greater activity, whereas lower concentrations showed reduced effects. Similar findings have been previously reported. Some herbicides, such as glyphosate, aromatic aldehydes, and isobenzofuranones, inhibit plant growth at high concentrations, but at lower concentrations they may show little inhibitory effect or may even promote plant growth [49,72,73].

Although the mode of action of dihydrochalcone-based herbicides has not been previously investigated, the significant inhibitory effects of several dihydrochalcones on seed germination and seedling growth of Chinese amaranth prompted further study [46,57]. Therefore, the effects of the most active compound, 3-chlorodihydrochalcone (**15**), were examined with respect to its interference with seed imbibition, inhibition of α-amylase activity, and induction of membrane damage in *A. tricolor*, as indicated by increased electrolyte leakage and malondialdehyde (MDA) accumulation. Notably, 3-chlorodihydrochalcone (**15**) showed significant effects in all of these assays. These findings are consistent with our previous report, in which the natural ketone herbicide xanthoxyline significantly interfered with seed imbibition and α-amylase activity in Chinese amaranth [46].

Finally, this study provides insight into how the type and position of substituent groups influence the herbicidal activity of dihydrochalcones, and preliminary modes of action for this class of compounds have been proposed. Given that many herbicidal modes of action are already known, further mechanistic investigations are warranted to achieve a more comprehensive understanding of the biological activity of dihydrochalcone-based herbicides [16,74,75,76,77].

In addition, only two plant species were evaluated in the present study. Broader biological assessment, including important crop species (e.g., rice, corn, and soybean), would be valuable to determine the selectivity and potential impact of these compounds on crops.

Moreover, future studies should include molecular docking analyses to predict the binding interactions of active dihydrochalcones with target enzymes, in comparison with known inhibitors. Such investigations would provide deeper insight into their potential mechanisms of action and further support their development as herbicidal agents [46].

## 3. Materials and Methods

### 3.1. Chemicals and Instruments

Commercial butachlor was obtained from Sinon Corporation (Bangkok, Thailand), while mesotrione was purchased from Sigma-Aldrich (St. Louis, MO, USA). Xanthoxyline was extracted from dried fruits of *Zanthoxylum limonella* following our previously reported method [46]. Flavokawains and their corresponding chalcone derivatives were synthesized according to established procedures [50,52].

Melting points were measured using a Gallenkamp melting point apparatus (Loughborough, UK). Infrared (IR) spectra were recorded on a PerkinElmer 8900 spectrometer (Shelton, CT, USA) at the Department of Chemistry, School of Science, King Mongkut’s Institute of Technology Ladkrabang (KMITL).

^1^H and ^13^C NMR spectra were acquired using a JEOL JNM-ECZ-500R/S1 spectrometer (500 MHz) (Tokyo, Japan) at the Scientific Instruments Centre, School of Science, KMITL. Chemical shifts (δ) are expressed in parts per million (ppm) relative to residual solvent peaks used as internal references: CDCl_3_ (δ 7.26 for ^1^H and 77.00 for ^13^C), acetone-d_6_ (δ 2.05 for ^1^H and 29.84 for ^13^C), CD_3_OD (δ 3.31 for ^1^H and 49.00 for ^13^C), and DMSO-d_6_ (δ 2.50 for ^1^H and 39.52 for ^13^C).

High-resolution mass spectrometry (HRMS) data were collected using an Agilent 1260 Infinity Series system (Agilent Technologies, Waldbronn, Germany) at the Faculty of Science, Naresuan University, Phitsanulok, Thailand.

### 3.2. Preparation of Dihydrochalcones (***1***–***27***)

A library of 27 dihydrochalcone derivatives containing diverse substituents–such as electron-donating groups, electron-withdrawing groups, and heterocyclic units–was prepared via catalytic hydrogenation using a method adapted from previously reported procedures [51].

*General Procedure 1*: Chalcone substrates (1 mmol) were combined with 10% palladium on carbon (0.01 g, 0.1 mmol) in a 100 mL round-bottom flask. Ethyl acetate (10 mL) was added as the solvent, and hydrogen gas was introduced using a balloon setup. The reaction mixture was stirred at ambient temperature for 24 h, and its progress was monitored by thin-layer chromatography (TLC). After completion, the mixture was filtered through silica gel under reduced pressure. The resulting filtrate was recrystallized from methanol to obtain the purified dihydrochalcone products. Structural characterization of all compounds was carried out using IR, NMR, and HRMS techniques (see Supplementary Materials).

### 3.3. Evaluation of the Herbicidal Activity of Dihydrochalcones (***1***–***27***)

#### 3.3.1. Preparation of 0.10% (*v*/*v*) Tween 80 Aqueous Solution

A stock solution of Tween 80 (0.10%, *v*/*v*) was prepared by dissolving 1.0 mL of Tween 80 in distilled water in a 1000 mL volumetric flask, following previously reported procedures [44,46,49]. This solution served as the negative control and was also used as the diluent for all test solutions in the bioassays.

#### 3.3.2. Preparation of 800 μM Aqueous Solutions of Dihydrochalcones and Reference Compounds

To prepare an 800 μM solution, dihydrochalcones (80 µmol) were mixed thoroughly with 0.1 mL of Tween 80 in a 50 mL beaker. Subsequently, 40 mL of distilled water was added, and the mixture was transferred to a 100 mL volumetric flask. The volume was adjusted to the mark with distilled water to yield an 800 μM solution containing 0.10% (*v*/*v*) Tween 80 [44,46,49].

The same procedure was employed to prepare 800 μM solutions of butachlor, mesotrione, and xanthoxyline, which were used as positive controls.

#### 3.3.3. Preparation of Dilution Series (1600–50 μM)

A 1600 μM stock solution of the active dihydrochalcones was first prepared using the method described above. This stock was then serially diluted with 0.10% (*v*/*v*) Tween 80 solution to obtain concentrations of 800, 400, 200, 100, and 50 μM.

#### 3.3.4. Plant Materials

Seeds of Chinese amaranth (*Amaranthus tricolor*) were obtained from Thai Seed & Agriculture Co., Ltd. (Bangkok, Thailand). Seeds of barnyard grass (*Echinochloa crus-galli*) were collected from rice fields in the Lat Krabang area of Bangkok, Thailand. All seed lots used in the experiments exhibited germination rates above 90%.

#### 3.3.5. Bioassay for Seed Germination and Seedling Growth

The bioassay was conducted using a vial-based method adapted from previous reports [44,46,49]. Briefly, 0.5 mL of the test solution was applied to a small vial (4.5 × 2.0 cm) lined with germination paper. Ten seeds of each plant species were placed in each vial, which was then sealed with Parafilm to prevent moisture loss.

The vials were incubated in a growth chamber (cool white 840 Climacell 707, Munich, Germany) under controlled conditions: 28–30 °C, a 12-h light/dark cycle (light intensity of 100 µmol m^−2^ s^−1^), and 80% relative humidity for 7 days.

Each treatment was carried out with four replicates. After the incubation period, germination was recorded, and shoot and root lengths were measured. The percentage inhibition of germination and seedling growth was calculated using the following equation:

Inhibition (% of control) = 100 − [(dihydrochalcone/Tween 80) × 100]

### 3.4. Mode of Mechanisms

#### 3.4.1. Evaluation of the Seed Imbibition and α-Amylase Activity

Dihydrochalcone **15** was investigated for its influence on seed imbibition and α-amylase activity. Treatment solutions were prepared using aqueous concentrations of compound **15** at 400, 800, 1600, and 3200 µM. A 3200 ppm Tween solution was employed as the solvent control, while distilled water served as the negative control. The study followed a completely randomized design with four replications.

The seed imbibition assay was adapted from the method reported by Turk and Tawaha [78]. In brief, 100 seeds of *A. tricolor* were weighed to obtain the initial mass (W_1_), then immersed in the respective treatment solutions for 3, 6, and 12 h. After soaking, the seeds were removed, gently dried to eliminate excess surface moisture, and weighed again (W_2_). Seed imbibition percentage was determined using the formula:

Seed imbibition (%) = [(W_2_ − W_1_)/W_1_] × 100

The activity of α-amylase was measured using the dinitrosalicylic acid (DNS) method as described by Sadasivam and Manickam [79]. Seeds were homogenized in 4 mL of 0.1 M CaCl_2_ under chilled conditions to extract the enzyme. The homogenate was then centrifuged at 10,000 rpm for 20 min at 4 °C, and the resulting supernatant was collected and stored at 4 °C for further analysis.

For the enzymatic assay, 1 mL of the supernatant was combined with 1 mL of 1% (*w*/*v*) soluble starch in acetate buffer (pH 5.5). The mixture was incubated at 37 °C for 15 min. The reaction was stopped by adding 1 mL of DNS reagent, followed by heating in boiling water (100 °C) for 5 min, and then rapidly cooled in an ice bath.

Absorbance was recorded at 560 nm using a UV–Vis spectrophotometer (Thermo Fisher Scientific, Waltham, MA, USA). The α-amylase activity was calculated and expressed as μmol maltose per minute per gram of fresh weight (FW).

#### 3.4.2. Relative Electrolyte Leakage (REL)

Relative electrolyte leakage (REL) in fresh seeds was assessed following a modified procedure based on Singh et al. [80]. Samples of *A. tricolor* seeds (200 mg) were treated with solutions of dihydrochalcone **15** at concentrations of 400, 800, 1600, and 3200 µM. A Tween 80 solution (3200 µM) was used as the solvent control, while deionized water served as the negative control. The seeds were incubated in the respective treatment solutions for 12 h.

Following treatment, the seeds were transferred into 20 mL of deionized water and kept at room temperature for an additional 24 h. The initial electrical conductivity (EC_1_) of the solution was then measured using a Consort C3010 multiparameter analyzer (Consort, Turnhout, Belgium). To achieve complete electrolyte release, the samples were subsequently boiled for 20 min, allowed to cool to room temperature, and the final electrical conductivity (EC_2_) was recorded.

REL was expressed as a percentage and calculated using the equation:

REL (%) = (EC1/EC2) × 100

#### 3.4.3. Lipid Peroxidation

Lipid peroxidation was evaluated by quantifying malondialdehyde (MDA), a key indicator of oxidative membrane damage, through the measurement of thiobarbituric acid-reactive substances (TBARS). *A. tricolor* seeds were exposed to the treatment solutions for 3, 6, and 12 h prior to analysis.

For the assay, 0.2 g of treated seeds were homogenized in 4 mL of 0.1% (*w*/*v*) trichloroacetic acid (TCA). The homogenate was then centrifuged at 6000× *g* for 20 min, and the resulting supernatant was collected. A portion of this extract was combined with a thiobarbituric acid (TBA) reagent containing 0.5% (*w*/*v*) TBA and 4% (*w*/*v*) butylated hydroxytoluene (BHT) prepared in TCA to form the reaction mixture.

The mixture was heated at 95 °C for 30 min, followed by rapid cooling in an ice bath. It was then centrifuged again at 6000× *g* for 10 min at 4 °C to clarify the solution. The absorbance of the supernatant was recorded at 532 and 600 nm using a UV–Vis spectrophotometer (Thermo Fisher Scientific, MA, USA). TBARS levels were calculated using an extinction coefficient of 155 mM/cm and expressed as nmol per gram of fresh weigh [81].

## 4. Conclusions

In summary, twenty-seven dihydrochalcones (**1**–**27**) were successfully synthesized from flavokawains and their chalcone analogues via Pd-catalyzed hydrogenation in high yields. The synthesized compounds were evaluated for their herbicidal activity against Chinese amaranth and barnyard grass. Several dihydrochalcones significantly inhibited seed germination as well as shoot and root growth in both plant species. The herbicidal activity was strongly influenced by the type and position of aromatic substituents and by concentration. In general, dihydrochalcones bearing electron-withdrawing groups and meta substitution exhibited higher inhibitory activity, and the effects increased with increasing concentration. The *meta*-chloro derivative (**15**) showed the highest activity against both test species. Preliminary mechanistic studies revealed that compound **15** interfered with seed imbibition, inhibited α-amylase activity, and induced membrane damage, as evidenced by increased electrolyte leakage and malondialdehyde (MDA) accumulation in *A. tricolor* in a concentration-dependent manner. Overall, these findings provide important insights into the structure–activity relationships (SAR) of dihydrochalcones and highlight their potential as lead compounds for the development of new herbicides derived from natural products.

## Data Availability

The original contributions presented in this study are included in the article and the Supplementary Materials. Further inquiries may be directed to the corresponding authors.

## Supplementary Materials

The following supporting information can be downloaded at: https://www.mdpi.com/article/10.3390/molecules31101587/s1, 1. Spectroscopic data for dihydrochalcones (**1**–**27**); 2. ^1^H NMR and ^13^C NMR spectra of dihydrochalcones (**1**–**27**) 3. Reference [82].

## Abbreviations

The following abbreviations are used in this manuscript:

BHT	Butylated hydroxytoluene
°C	Degree Celsius
cm	Centimeter
DNS	Dinitrosalicylic acid
FW	Fresh weight
h	Hour
HRMS	High-resolution mass spectrometra
IR	Infrared
min	Minute
mL	Milliliter
µM	Micromolar
µmol	Micromole
MDA	Malondialdehyde
nm	Nanometer
NMR	Nuclear Magnetic Resonance
REL	Relative electrolyte leakage
ROS	Reactive oxygen species
TBA	Thiobarbituric acid
TBARS	Thiobarbituric acid-Reactive substances
TCA	Trichloroacetic acid
TLC	Thin-layer chromatography
UV–Vis	Ultraviolet–Visible
*v*/*v*	Volume-to-volume
*w*/*v*	Weight-to-volume
