# Supplementary material for: Design, Synthesis, and Herbicidal Activity of Novel Dihydrochalcones Derived from Flavokawains and Their Analogs
Source: Molecules. 2026 May 9;31(10):1587. doi: 10.3390/molecules31101587 (PMC13210083; doi:10.3390/molecules31101587)
Supplement: Supplementary file 1 [file molecules-31-01587-s001.zip › molecules-4195415-supplementary.pdf]

Supplementary Material

Design, Synthesis, and Herbicidal Activity of Novel Dihydrochalcones Derived from Flavokawains and Their Analogs

Suriyaphong Poprom [1], Jatuporn Meesin [1], Warot Chotpatiwetchkul [2], Watcharee Waratchareeyakul [3], Nawasit Chotsaeng [1,*], Chamroon Laosinwattana [4] and Naphat Somala [4]

[1] Department of Chemistry and Advanced Pure and Applied Chemistry Research Unit, School of Science, King Mongkut's Institute of Technology Ladkrabang, Bangkok 10520, Thailand
[2] Department of Chemistry and Applied Computational Chemistry Research Unit, School of Science, King Mongkut's Institute of Technology Ladkrabang, Bangkok 10520, Thailand
[3] Department of Chemistry, Faculty of Science and Technology, Rambhai Barni Rajabhat University, Chanthaburi 22000, Thailand
[4] Department of Plant Production Technology, School of Agricultural Technology, King Mongkut's Institute of Technology Ladkrabang, Bangkok 10520, Thailand
* Correspondence: nawasit.ch@kmitl.ac.th; Tel.: +66-2329-8400

Total number of Pages: 40 (Pages 1-40).

Table of contents

		page
1	Spectroscopic data for dihydrochalcones (**1–27**)	2
2	^{1}H NMR and ^{13}C NMR spectra of dihydrochalcones (**1–27**)	13
3	Reference	40

1) Spectroscopic data for dihydrochalcones (1–27)

1-(2-hydroxy-4,6-dimethoxyphenyl)-3-phenylpropan-1-one (1)

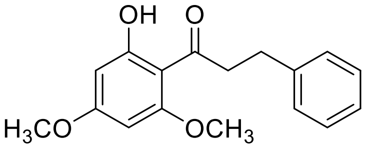

The title compound was obtained as a white solid (92%).
m.p. 95–96 °C (MeOH);
Rf = 0.51 (10% EtOAc/hexane);
IR (film) 2924, 1732, 1622, 1587, 1276, 1209, 1155, 1116, 1043, 817 cm^{-1};
^{1}H NMR (500 MHz, $CDCl_3$) δ 14.02 (1H, s, O**H**), 7.30 (2H, t, *J* = 6.0 Hz, Ar**H**), 7.25 (2H, d, *J* = 6.2 Hz, Ar**H**), 7.21 (1H, t, *J* = 5.7 Hz, Ar**H**), 6.08 (1H, d, *J* = 1.7 Hz, Ar**H**), 5.93 (1H, d, *J* = 1.5 Hz, Ar**H**), 3.84 (3H, s, OC**H**$_3$), 3.82 (3H, s, OC**H**$_3$), 3.34 – 3.31 (2H, m, C**H**$_2$), 3.02 – 2.98 (2H, m, C**H**$_2$).
^{13}C NMR (125.8 MHz, $CDCl_3$) δ 204.6, 167.7, 166.0, 162.8, 141.8, 128.5 (2 × CH), 128.5 (2 × CH), 126.0, 105.8, 93.7, 90.9, 55.7, 55.6, 45.8, 30.8.
HRMS (ESI) Exact mass calcd for $C_{17}H_{19}O_4$ $[M+H]^+$: 287.1283, found 287.1276.
The spectroscopic data were in agreement with the literature.[1]

1-(2-hydroxy-4,6-dimethoxyphenyl)-3-(o-tolyl)propan-1-one (2)

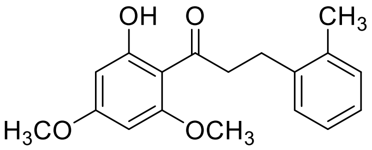

The title compound was obtained as a white solid (93%).
m.p. 101-102 °C (MeOH);
Rf = 0.58 (10% EtOAc/hexane);
IR (film) 2943, 1618, 1589, 1417, 1273, 1207, 1157, 1114, 819 cm^{-1};
^{1}H NMR (500 MHz, $CDCl_3$) δ 14.04 (1H, s, O**H**), 7.16 (2H, d, *J* = 3.3 Hz, Ar**H**), 7.15 – 7.12 (2H, m, Ar**H**), 6.08 (1H, s, Ar**H**), 5.93 (1H, s, Ar**H**), 3.82 (6H, s, 2 × OC**H**$_3$), 3.30 – 3.26 (2H, m, C**H**$_2$), 3.00 – 2.96 (2H, m, C**H**$_2$), 2.35 (3H, s, C**H**$_3$).
^{13}C NMR (125.8 MHz, $CDCl_3$) δ 204.8, 167.8, 166.0, 162.8, 139.8, 136.2, 130.3, 128.8, 126.2, 126.1, 105.9, 93.8, 90.9, 55.7, 55.6, 44.3, 28.1, 19.4.
HRMS (ESI) Exact mass calcd for $C_{18}H_{21}O_4$ $[M+H]^+$: 301.1440, found 301.1435.

1-(2-hydroxy-4,6-dimethoxyphenyl)-3-(m-tolyl)propan-1-one (3)

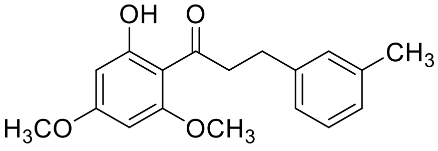

The title compound was obtained as a white solid (92%).
m.p. 103-104 °C (MeOH);
Rf = 0.62 (10% EtOAc/hexane);
IR (film) 2941, 1618, 1585, 1417, 1273, 1207, 1157, 1114, 970, 819 cm^{-1};

^{1}H NMR (500 MHz, $CDCl_3$) δ 14.02 (1H, s, O**H**), 7.19 (1H, t, *J* = 6.0 Hz, Ar**H**), 7.06 (1H, s, Ar**H**), 7.05 – 7.00 (2H, m, Ar**H**), 6.08 (1H, d, *J* = 1.4 Hz, Ar**H**), 5.93 (1H, d, *J* = 1.2 Hz, Ar**H**), 3.84 (3H, s, OC**H**$_3$), 3.82 (3H, s, OC**H**$_3$), 3.33 – 3.29 (2H, m, C**H**$_2$), 2.98 – 2.94 (2H, m, C**H**$_2$), 2.34 (3H, s, C**H**$_3$).
^{13}C NMR (125.8 MHz, $CDCl_3$) δ 204.7, 167.8, 166.0, 162.8, 141.7, 138.1, 129.4, 128.4, 126.8, 125.5, 105.8, 93.7, 90.9, 55.7, 55.6, 45.9, 30.7, 21.5.
HRMS (ESI) Exact mass calcd for $C_{18}H_{21}O_4$ $[M+H]^+$: 301.1440, found 301.1435.

1-(2-hydroxy-4,6-dimethoxyphenyl)-3-(p-tolyl)propan-1-one (4)

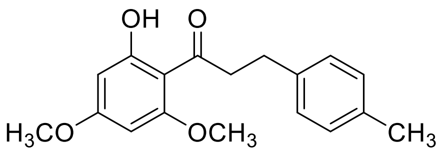

The title compound was obtained as a white solid (94%).
m.p. 113-114 °C (MeOH);
Rf = 0.62 (10% EtOAc/hexane);
IR (film) 2943, 1622, 1587, 1415, 1365, 1159, 1112, 972, 819 cm^{-1};
^{1}H NMR (500 MHz, $CDCl_3$) δ 14.03 (1H, s, O**H**), 7.12 (4H, q, *J* = 6.4 Hz, 2 × Ar**H**), 6.07 (1H, d, *J* = 1.6 Hz, Ar**H**), 5.92 (1H, s, Ar**H**), 3.83 (3H, s, OC**H**$_3$), 3.82 (3H, s, OC**H**$_3$), 3.32 – 3.28 (2H, m, C**H**$_2$), 2.97 – 2.93 (2H, m, C**H**$_2$), 2.33 (3H, s, C**H**$_3$).
^{13}C NMR (125.8 MHz, $CDCl_3$) δ 204.7, 167.8, 166.0, 162.8, 138.7, 135.5, 129.2 (2 × CH), 128.4 (2 × CH), 105.8, 93.7, 90.9, 55.7, 55.6, 46.0, 30.4, 21.1.
HRMS (ESI) Exact mass calcd for $C_{18}H_{21}O_4$ $[M+H]^+$: 301.1440, found 301.1434.

1-(2-hydroxy-4,6-dimethoxyphenyl)-3-(2-hydroxyphenyl)propan-1-one (5)

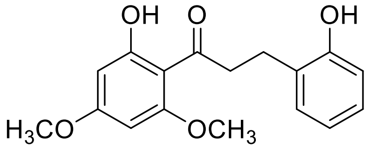

The title compound was obtained as a white solid (93%).
m.p. 127-128 °C (MeOH);
Rf = 0.14 (20% EtOAc/hexane);
IR (film) 3369, 332941, 1618, 1583, 1417, 1367, 1159, 1114, 970, 819 cm^{-1};
^{1}H NMR (500 MHz, $CDCl_3$) δ 13.50 (1H, s, O**H**), 7.61 (1H, s, O**H**), 7.14 – 7.09 (2H, m, Ar**H**), 6.89 (1H, d, *J* = 6.4 Hz, Ar**H**), 6.85 (1H, t, *J* = 5.9 Hz, Ar**H**), 6.05 (1H, d, *J* = 1.3 Hz, Ar**H**), 5.91 (1H, d, *J* = 1.5 Hz, Ar**H**), 3.86 (3H, s, OC**H**$_3$), 3.81 (3H, s, OC**H**$_3$), 3.46 – 3.42 (2H, m, C**H**$_2$), 2.99 – 2.92 (2H, m, C**H**$_2$).
^{13}C NMR (125.8 MHz, $CDCl_3$) δ 205.6, 167.7, 166.7, 163.3, 154.5, 130.7, 128.2, 127.9, 120.6, 117.2, 105.8, 93.8, 91.2, 55.8, 55.7, 45.7, 23.7.
HRMS (ESI) Exact mass calcd for $C_{17}H_{19}O_5$ $[M+H]^+$: 303.1232, found 303.1224.

1-(2-hydroxy-4,6-dimethoxyphenyl)-3-(3-hydroxyphenyl)propan-1-one (6)

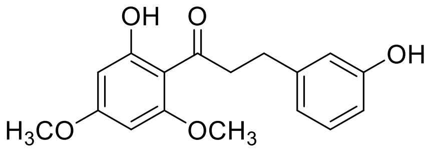

The title compound was obtained as a white solid (96%).
m.p. 113-114 °C (MeOH);
Rf = 0.14 (20% EtOAc/hexane);
IR (film) 3390, 2929, 1618, 1549, 1417, 1365, 1157, 1114, 970, 821 cm^{-1};
^{1}H NMR (500 MHz, $CDCl_3$) δ 14.02 (1H, s, O**H**), 7.16 (1H, t, *J* = 6.3 Hz, Ar**H**), 6.81 (1H, d, *J* = 6.0 Hz, Ar**H**), 6.72 (1H, s, Ar**H**), 6.68 (1H, d, *J* = 6.5 Hz, Ar**H**), 6.07 (1H, s, Ar**H**), 5.93 (1H, d, *J* = 1.5 Hz, Ar**H**), 4.97 (1H, s, O**H**), 3.83 (3H, s, OC**H**$_3$), 3.82 (3H, s, OC**H**$_3$), 3.32 – 3.28 (2H, m, C**H**$_2$), 2.97 – 2.93 (2H, m, C**H**$_2$).
^{13}C NMR (125.8 MHz, $CDCl_3$) δ 204.5, 167.7, 166.1, 162.8, 155.7, 143.8, 129.7, 121.0, 115.5, 113.0, 105.8, 93.7, 90.1, 55.7, 55.6, 45.6, 30.6.
HRMS (ESI) Exact mass calcd for $C_{17}H_{19}O_5$ $[M+H]^+$: 303.1232, found 303.1229.

1-(2-hydroxy-4,6-dimethoxyphenyl)-3-(4-hydroxyphenyl)propan-1-one (7)

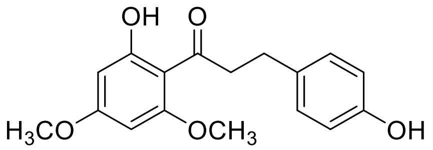

The title compound was obtained as a white solid (96%).
m.p. 113-114 °C (MeOH);
Rf = 0.08 (20% EtOAc/hexane);
IR (film) 3392, 2933, 1618, 1585, 1417, 1365, 1159, 1114, 970, 821 cm^{-1};
^{1}H NMR (500 MHz, $CDCl_3$) δ 14.03 (1H, s, O**H**), 7.10 (2H, d, *J* = 6.4 Hz, Ar**H**), 6.76 (2H, d, *J* = 6.3 Hz, Ar**H**), 6.07 (1H, d, *J* = 1.7 Hz, Ar**H**), 5.92 (1H, d, *J* = 1.5 Hz, Ar**H**), 4.84 (1H, s, O**H**), 3.83 (3H, s, OC**H**$_3$), 3.82 (3H, s, OC**H**$_3$), 3.30 – 3.24 (2H, m, C**H**$_2$), 2.92 (2H, t, *J* = 6.1 Hz, C**H**$_2$).
^{13}C NMR (125.8 MHz, $CDCl_3$) δ 204.7, 167.8, 166.0, 162.8, 153.8, 133.9, 129.6 (2 × CH), 115.3 (2 × CH) , 105.8, 93.7, 90.9, 55.7, 55.6, 46.1, 29.9.
HRMS (ESI) Exact mass calcd for $C_{17}H_{19}O_5$ $[M+H]^+$: 303.1232, found 303.1229.

1-(2-hydroxy-4,6-dimethoxyphenyl)-3-(2-methoxyphenyl)propan-1-one (8)

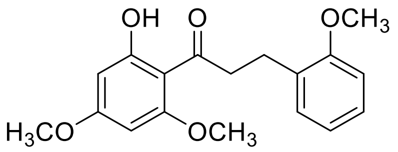

The title compound was obtained as a white solid (93%).
m.p. 99-100 °C (MeOH);
Rf = 0.42 (10% EtOAc/hexane);
IR (film) 2937, 1616, 1585, 1415, 1365, 1157, 1112, 970, 819 cm^{-1};
^{1}H NMR (500 MHz, $CDCl_3$) δ 14.10 (1H, s, O**H**), 7.21 – 7.17 (2H, m, Ar**H**), 6.90 (1H, t, *J* = 5.9 Hz, Ar**H**), 6.86 (1H, d, *J* = 6.5 Hz, Ar**H**), 6.07 (1H, d, *J* = 1.7 Hz, Ar**H**), 5.91 (1H, d, *J* = 1.7 Hz, Ar**H**), 3.82 (6H, s, 2 × OC**H**$_3$), 3.81 (3H, s, OC**H**$_3$), 3.31 – 3.27 (2H, m, C**H**$_2$), 3.02 – 2.97 (2H, m, C**H**$_2$).
^{13}C NMR (125.8 MHz, $CDCl_3$) δ 205.3, 167.7, 165.9, 162.9, 157.7, 130.0 (2 × CH), 127.3, 120.5, 110.3, 105.9, 93.7, 90.3, 55.6 (2 × CH_3), 55.3, 44.2, 25.6.
HRMS (ESI) Exact mass calcd for $C_{18}H_{21}O_5$ $[M+H]^+$: 317.1389, found 317.1384.

1-(2-hydroxy-4,6-dimethoxyphenyl)-3-(3-methoxyphenyl)propan-1-one (9)

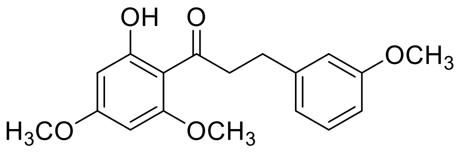

The title compound was obtained as a white solid (96%).
m.p. 95-96 °C (MeOH);
Rf = 0.42 (10% EtOAc/hexane);
IR (film) 2941, 1618, 1585, 1417, 1365, 1157, 1114, 970, 821 cm^{-1};
^{1}H NMR (500 MHz, $CDCl_3$) δ 14.02 (1H, s, O**H**), 7.22 (1H, t, *J* = 6.3 Hz, Ar**H**), 6.84 (1H, d, *J* = 6.1 Hz, Ar**H**), 6.80 (1H, s, Ar**H**), 6.76 (1H, d, *J* = 6.5 Hz, Ar**H**), 6.07 (1H, d, *J* = 1.2 Hz, Ar**H**), 5.92 (1H, d, *J* = 1.4 Hz, Ar**H**), 3.84 (3H, s, OC**H**$_3$), 3.82 (3H, s, OC**H**$_3$), 3.80 (3H, s, OC**H**$_3$), 3.35 – 3.29 (2H, m, C**H**$_2$), 3.00 – 2.93 (2H, m, C**H**$_2$).
^{13}C NMR (125.8 MHz, $CDCl_3$) δ 204.5, 167.8, 166.0, 162.8, 159.7, 143.5, 129.5, 120.9, 114.4, 111.2, 105.8, 93.7, 90.9, 55.7, 55.7, 55.2, 45.7, 30.8.
HRMS (ESI) Exact mass calcd for $C_{18}H_{21}O_5$ $[M+H]^+$: 317.1389, found 317.1387.

1-(2-hydroxy-4,6-dimethoxyphenyl)-3-(4-methoxyphenyl)propan-1-one (10)

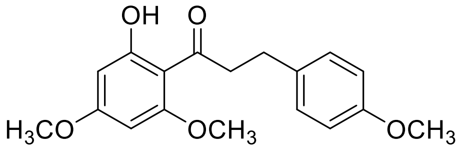

The title compound was obtained as a white solid (93%).
m.p. 95-96 °C (MeOH);
Rf = 0.42 (10% EtOAc/hexane);
IR (film) 2941, 1614, 1585, 1417, 1365, 1217, 1159, 1114, 970, 821 cm^{-1};
^{1}H NMR (500 MHz, $CDCl_3$) δ 14.04 (1H, s, O**H**), 7.16 (2H, d, *J* = 6.7 Hz, Ar**H**), 6.84 (2H, d, *J* = 6.9 Hz, Ar**H**), 6.07 (1H, s, Ar**H**), 5.92 (1H, d, *J* = 1.3 Hz, Ar**H**), 3.83 (3H, s, OC**H**$_3$), 3.82 (3H, s, OC**H**$_3$), 3.79 (3H, s, OC**H**$_3$), 3.31 – 3.26 (2H, m, C**H**$_2$), 2.95 – 2.91 (2H, m, C**H**$_2$).
^{13}C NMR (125.8 MHz, $CDCl_3$) δ 204.7, 167.8, 166.0, 162.8, 157.9, 133.8, 129.4 (2 × CH), 113.9 (2 × CH), 105.8, 93.7, 90.9, 55.7, 55.6, 55.4, 46.1, 29.9.
HRMS (ESI) Exact mass calcd for $C_{18}H_{21}O_5$ $[M+H]^+$: 317.1389, found 317.1389.

3-(2-fluorophenyl)-1-(2-hydroxy-4,6-dimethoxyphenyl)propan-1-one (11)

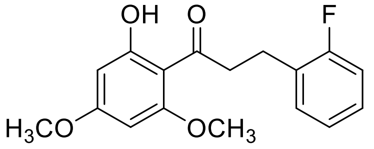

The title compound was obtained as a white solid (93%).
m.p. 83-84 °C (MeOH);
Rf = 0.40 (10% EtOAc/hexane);
IR (film) 2941, 1618, 1585, 1417, 1365, 1209, 1157, 1114, 970, 819 cm^{-1};
^{1}H NMR (500 MHz, $CDCl_3$) δ 14.00 (1H, s, O**H**), 7.25 (1H, t, *J* = 6.1 Hz, Ar**H**), 7.19 (1H, dd, *J* = 10.8, 5.7 Hz, Ar**H**), 7.06 (1H, t, *J* = 6.1 Hz, Ar**H**), 7.04 – 7.00 (1H, m, Ar**H**), 6.07 (1H, d, *J* = 1.4 Hz, Ar**H**), 5.92 (1H, d, *J* = 1.3 Hz, Ar**H**), 3.83 (3H, s, OC**H**$_3$), 3.82 (3H, s, OC**H**$_3$), 3.33 – 3.29 (2H, m, C**H**$_2$), 3.02 (2H, t, *J* = 6.1 Hz, C**H**$_2$).

^{13}C NMR (125.8 MHz, $CDCl_3$) δ 204.3, 167.7, 166.1, 162.8, 162.3, 160.4, 131.0 (d, *J* = 14.8 Hz), 128.5 (d, *J* = 50.0 Hz), 127.8 (d, *J* = 25.5 Hz), 124.1 (d, *J* = 9.0 Hz), 115.3 (d, *J* = 70.4 Hz), 105.8, 93.7, 90.9, 55.6 (2 × CH_3), 44.2, 24.3.
HRMS (ESI) Exact mass calcd for $C_{17}H_{18}FO_4$ $[M+H]^+$: 305.1189, found 305.1185.

3-(3-fluorophenyl)-1-(2-hydroxy-4,6-dimethoxyphenyl)propan-1-one (12)

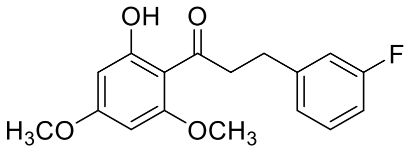

The title compound was obtained as a white solid (96%).
m.p. 83-84 °C (MeOH);
Rf = 0.30 (10% EtOAc/hexane);
IR (film) 2972, 1618, 1585, 1423, 1359, 1207, 1155, 1118, 950, 817 cm^{-1};
^{1}H NMR (500 MHz, $CDCl_3$) δ 13.97 (1H, s, O**H**), 7.26 – 7.22 (1H, m, Ar**H**), 7.02 (1H, d, *J* = 6.1 Hz, Ar**H**), 6.94 (1H, d, *J* = 8.1 Hz, Ar**H**), 6.89 (1H, td, *J* = 7.0, 1.6 Hz, Ar**H**), 6.07 (1H, d, *J* = 1.7 Hz, Ar**H**), 5.93 (1H, d, *J* = 1.7 Hz, Ar**H**), 3.84 (3H, s, OC**H**3), 3.82 (3H, s, OC**H**3), 3.33 – 3.29 (2H, m, C**H**2), 3.01 – 2.98 (2H, m, C**H**2)
^{13}C NMR (125.8 MHz, $CDCl_3$) δ 204.0, 167.8, 166.1, 164.0, 162.8, 162.0, 144.4 (d, *J* = 22.9 Hz), 129.9 (d, *J* = 27.1 Hz), 124.2 (d, *J* = 7.1 Hz), 115.4 (d, *J* = 67.4 Hz), 112.9 (d, *J* = 65.7 Hz), 105.7, 93.7, 91.0, 55.7, 55.7, 45.4, 30.4.
HRMS (ESI) Exact mass calcd for $C_{17}H_{18}FO_4$ $[M+H]^+$: 305.1189, found 305.1185.

3-(4-fluorophenyl)-1-(2-hydroxy-4,6-dimethoxyphenyl)propan-1-one (13)

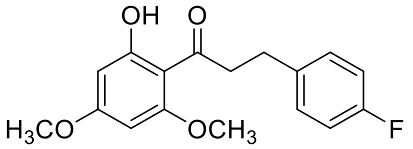

The title compound was obtained as a white solid (96%).
m.p. 79-80 °C (MeOH);
Rf = 0.40 (10% EtOAc/hexane);
IR (film) 2927, 1614, 1583, 1417, 1367, 1205, 1157, 1114, 970, 819 cm^{-1};
^{1}H NMR (500 MHz, $CDCl_3$) δ 13.98 (1H, s, O**H**), 7.19 (2H, dd, *J* = 6.4, 4.6 Hz, Ar**H**), 6.97 (2H, t, *J* = 6.9 Hz, Ar**H**), 6.07 (1H, d, *J* = 1.5 Hz, Ar**H**), 5.92 (1H, d, *J* = 1.5 Hz, Ar**H**), 3.83 (3H, s, OC**H**3), 3.82 (3H, s, OC**H**3), 3.31 – 3.27 (2H, m, C**H**2), 2.99 – 2.95 (2H, m, C**H**2).
^{13}C NMR (125.8 MHz, $CDCl_3$) δ 204.3, 167.8, 166.1, 162.8, 162.4, 160.4, 137.4, 129.9 (d, *J* = 25.4 Hz), 115.2 (d, *J* = 66.1 Hz), 105.8, 93.8, 90.9, 55.7, 55.6, 45.8, 29.9.
HRMS (ESI) Exact mass calcd for $C_{17}H_{18}FO_4$ $[M+H]^+$: 305.1189, found 305.1183.

3-(2-chlorophenyl)-1-(2-hydroxy-4,6-dimethoxyphenyl)propan-1-one (14)

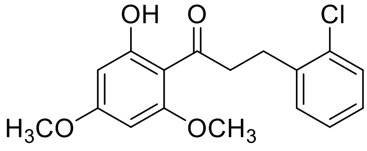

The title compound was obtained as a white solid (93%).
m.p. 91-92 °C (MeOH);
Rf = 0.70 (10% EtOAc/hexane);

IR (film) 2941, 1616, 1583, 1417, 1367, 1207, 1157, 1112, 970, 819 cm^{-1};
^{1}H NMR (500 MHz, $CDCl_3$) δ 14.02 (1H, s, O**H**), 7.31 (2H, t, *J* = 6.0 Hz, Ar**H**), 7.25 (1H, s, Ar**H**), 7.21 (1H, t, *J* = 5.6 Hz, Ar**H**), 6.09 (1H, d, *J* = 1.5 Hz, Ar**H**), 5.93 (1H, d, *J* = 1.4 Hz, Ar**H**), 3.84 (3H, s, OC**H**3), 3.83 (3H, s, OC**H**3), 3.35 – 3.31 (2H, m, C**H**2), 3.03 – 2.98 (2H, m, C**H**2)
^{13}C NMR (125.8 MHz, $CDCl_3$) δ 204.6, 167.8, 166.0, 162.8, 141.8, 128.5 (2 × CH), 128.5 (2 × CH), 126.0, 105.8, 93.7, 90.9, 55.7, 55.6, 45.8, 30.8.
HRMS (ESI) Exact mass calcd for $C_{17}H_{18}ClO_4$ [M+H]$^+$: 321.0893, found 321.0889.

3-(3-chlorophenyl)-1-(2-hydroxy-4,6-dimethoxyphenyl)propan-1-one (15)

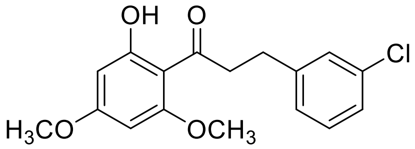

The title compound was obtained as a white solid (93%).
m.p. 91-92 °C (MeOH);
Rf = 0.70 (10% EtOAc/hexane);
IR (film) 2941, 1614, 1585, 1417, 1365, 1207, 1157, 1114, 970, 819 cm^{-1};
^{1}H NMR (500 MHz, $CDCl_3$) δ 14.02 (1H, s, O**H**), 7.31 (2H, t, *J* = 6.0 Hz, Ar**H**), 7.24 (1H, s, Ar**H**), 7.21 (1H, t, *J* = 6.2 Hz, Ar**H**), 6.09 (1H, d, *J* = 1.5 Hz, Ar**H**), 5.93 (1H, s, Ar**H**), 3.84 (3H, s, OC**H**3), 3.83 (3H, s, OC**H**3), 3.35 – 3.31 (2H, m, C**H**2), 3.03 – 2.99 (2H, m, C**H**2).
^{13}C NMR (125.8 MHz, $CDCl_3$) δ 204.6, 167.8, 166.0, 162.8, 141.8, 128.5 (2 × CH), 128.5 (2 × CH), 126.0, 105.8, 93.8, 90.9, 55.7, 55.6, 45.8, 30.8.
HRMS (ESI) Exact mass calcd for $C_{17}H_{18}ClO_4$ [M+H]$^+$: 321.0893, found 321.0888.

3-(4-chlorophenyl)-1-(2-hydroxy-4,6-dimethoxyphenyl)propan-1-one (16)

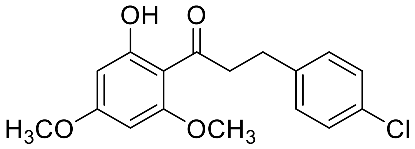

The title compound was obtained as a white solid (93%).
m.p. 91-92 °C (MeOH);
Rf = 0.70 (10% EtOAc/hexane);
IR (film) 2941, 1614, 1585, 1417, 1365, 1207, 1157, 1114, 970, 819 cm^{-1};
^{1}H NMR (500 MHz, $CDCl_3$) δ 14.03 (1H, s, O**H**), 7.30 (d, *J* = 5.8 Hz, Ar**H**), 7.24 (d, *J* = 8.0 Hz, Ar**H**), 6.09 (1H, d, *J* = 1.6 Hz, Ar**H**), 5.93 (1H, s, Ar**H**), 3.84 (3H, s, OC**H**3), 3.83 (3H, s, OC**H**3), 3.35 – 3.31 (2H, m, C**H**2), 3.03 – 2.98 (2H, m, C**H**2).
^{13}C NMR (125.8 MHz, $CDCl_3$) δ 204.6, 167.8, 166.0, 162.8, 141.8, 128.5 (2 × CH), 128.5 (2 × CH), 126.0, 105.8, 93.8, 90.9, 55.7, 55.6, 45.8, 30.8.
HRMS (ESI) Exact mass calcd for $C_{17}H_{18}ClO_4$ [M+H]$^+$: 321.0893, found 321.0889.

3-(2-bromophenyl)-1-(2-hydroxy-4,6-dimethoxyphenyl)propan-1-one (17)

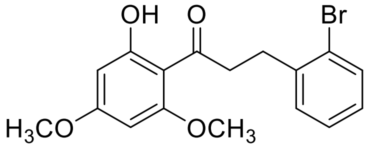

The title compound was obtained as a white solid (97%).
m.p. 83-84 °C (MeOH);
Rf = 0.65 (10% EtOAc/hexane);
IR (film) 2929, 1618, 1593, 1417, 1367, 1273, 1159, 1114, 970, 819 cm^{-1};
^{1}H NMR (500 MHz, $CDCl_3$) δ 14.01 (1H, s, O**H**), 7.32 – 7.28 (2H, m, Ar**H**) 7.25 (1H, d, *J* = 6.1 Hz, Ar**H**), 7.20 (1H, t, *J* = 5.9 Hz, Ar**H**), 6.08 (1H, d, *J* = 1.5 Hz, Ar**H**), 5.93 (1H, s, Ar**H**), 3.83 (3H, s, OC**H**3), 3.82 (3H, s, OC**H**3), 3.35 – 3.30 (2H, m, C**H**2), 3.02 – 2.98 (2H, m, C**H**2).
^{13}C NMR (125.8 MHz, $CDCl_3$) δ 204.6, 167.8, 166.0, 162.8, 141.8, 128.5 (2 × CH), 128.5 (2 × CH), 126.0, 105.8, 93.8, 90.9, 55.7, 55.6, 45.8, 30.8.
HRMS (ESI) Exact mass calcd for $C_{17}H_{18}BrO_4$ $[M+H]^+$: 365.0388, found 365.0383.

3-(3-bromophenyl)-1-(2-hydroxy-4,6-dimethoxyphenyl)propan-1-one (18)

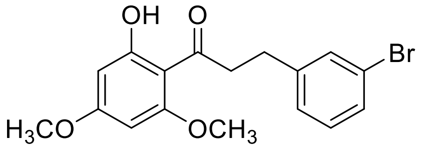

The title compound was obtained as a white solid (91%).
m.p. 69-70 °C (MeOH);
Rf = 0.63 (10% EtOAc/hexane);
IR (film) 2941, 1616, 1589, 1417, 1367, 1273, 1157, 1114, 970, 819 cm^{-1};
^{1}H NMR (500 MHz, $CDCl_3$) δ 14.01 (1H, s, O**H**), 7.29 (2H, d, *J* = 5.8 Hz, Ar**H**), 7.24 (1H, s, Ar**H**), 7.20 (1H, t, *J* = 5.8 Hz, Ar**H**), 6.08 (1H, d, *J* = 1.2 Hz, Ar**H**), 5.93 (1H, s, Ar**H**), 3.83 (3H, s, OC**H**3), 3.82 (3H, s, OC**H**3), 3.35 – 3.30 (2H, m, C**H**2), 3.00 (2H, t, *J* = 6.2 Hz, C**H**2);
^{13}C NMR (125.8 MHz, $CDCl_3$) δ 204.6, 167.8, 166.0, 162.8, 141.8, 128.6 (2 × CH), 128.5 (2 × CH), 126.0, 105.8, 93.7, 90.9, 55.7, 55.7, 45.8, 30.8.
HRMS (ESI) Exact mass calcd for $C_{17}H_{18}BrO_4$ $[M+H]^+$: 365.0388, found 365.0392.

3-(4-bromophenyl)-1-(2-hydroxy-4,6-dimethoxyphenyl)propan-1-one (19)

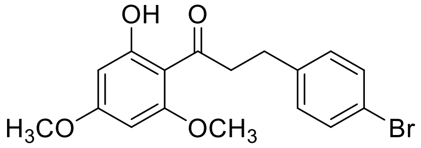

The title compound was obtained as a white solid (88%).
m.p. 85-86 °C (MeOH);
Rf = 0.66 (10% EtOAc/hexane);
IR (film) 2927, 1616, 1589, 1417, 1367, 1277, 1157, 1114, 970, 821 cm^{-1};
^{1}H NMR (500 MHz, $CDCl_3$) δ 14.00 (1H, s, O**H**), 7.29 (2H, d, *J* = 5.8 Hz, Ar**H**), 7.25 (2H, d, *J* = 6.4 Hz, Ar**H**), 6.07 (1H, d, *J* = 1.4 Hz, Ar**H**), 5.92 (1H, d, *J* = 1.5 Hz, Ar**H**), 3.82 (3H, s, OC**H**3), 3.81 (3H, s, OC**H**3), 3.34 – 3.29 (2H, m, C**H**2), 3.01 – 2.97 (2H, m, C**H**2).

^{13}C NMR (125.8 MHz, $CDCl_3$) δ 204.6, 167.8, 166.0, 162.8, 141.8, 128.5 (2 × CH), 128.5 (2 × CH), 126.0, 105.8, 93.8, 90.9, 55.7, 55.6, 45.8, 30.8.
HRMS (ESI) Exact mass calcd for $C_{17}H_{18}BrO_4$ $[M+H]^+$: 365.0388, found 365.0382.

3-(3-(2-hydroxy-4,6-dimethoxyphenyl)-3-oxopropyl)benzoic acid (20)

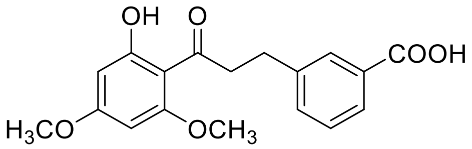

The title compound was obtained as a white solid (93%).
m.p. 161-162 °C (MeOH);
Rf = 0.07 (20% EtOAc/hexane);
IR (film) 2923, 1612, 1596, 1421, 1373, 1271, 1159, 1116, 974, 819 cm^{-1};
^{1}H NMR (500 MHz, $CDCl_3$) δ 13.98 (1H, s, O**H**), 7.99 (1H, s, ArH), 7.94 (1H, d, *J* = 6.2 Hz, Ar**H**), 7.49 (1H, d, *J* = 6.0 Hz, Ar**H**), 7.39 (1H, t, *J* = 6.1 Hz, Ar**H**), 6.07 (1H, d, *J* = 1.4 Hz, Ar**H**), 5.91 (1H, d, *J* = 1.4 Hz, Ar**H**), 3.84 (3H, s, OC**H**$_3$), 3.81 (3H, s, OC**H**$_3$), 3.33 (2H, t, *J* = 6.1 Hz, C**H**$_2$), 3.05 (2H, t, *J* = 6.1 Hz, C**H**$_2$), 2.60 (1H, s, COO**H**).
^{13}C NMR (125.8 MHz, $CDCl_3$) δ 204.0, 171.6, 167.8, 166.1, 162.8, 142.3, 134.2, 130.2, 129.5, 128.7, 128.0, 105.7, 93.7, 91.0, 55.8, 55.7, 45.5, 30.5.
HRMS (ESI) Exact mass calcd for $C_{18}H_{19}O_6$ $[M+H]^+$: 331.1181, found 331.1174.

4-(3-(2-hydroxy-4,6-dimethoxyphenyl)-3-oxopropyl)benzoic acid (21)

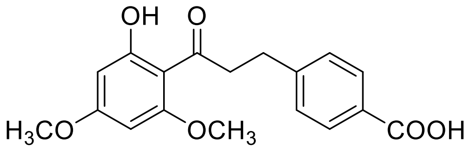

The title compound was obtained as a white solid (93%).
m.p. 259-260 °C (MeOH);
Rf = 0.08 (20% EtOAc/hexane);
IR (film) 2924, 1612, 1597, 1421, 1373, 1273, 1159, 1116, 974, 821 cm^{-1};
^{1}H NMR (500 MHz, $CDCl_3$) δ 13.94 (1H, s, O**H**), 8.03 (2H, d, *J* = 6.3 Hz, Ar**H**), 7.35 (2H, d, *J* = 6.4 Hz, Ar**H**), 6.08 (1H, d, *J* = 1.2 Hz, Ar**H**), 5.93 (1H, d, *J* = 1.4 Hz, Ar**H**), 3.83 (3H, s, OC**H**$_3$), 3.82 (3H, s, OC**H**$_3$), 3.35 (2H, t, *J* = 6.1 Hz, C**H**$_2$), 3.07 (2H, t, *J* = 6.1 Hz, C**H**$_2$), 2.43 (1H, s, COO**H**);
^{13}C NMR (125.8 MHz, $CDCl_3$) δ 203.8, 167.8, 166.2, 162.8, 148.4, 130.5 (2 × CH), 129.3, 128.7 (2 × CH), 127.1, 105.7, 93.8, 91.0, 55.7, 55.7, 45.1, 30.7.
HRMS (ESI) Exact mass calcd for $C_{18}H_{19}O_6$ $[M+H]^+$: 331.1181, found 331.1173.

1-(2-hydroxy-4,6-dimethoxyphenyl)-3-(4-isopropoxyphenyl)propan-1-one (22)

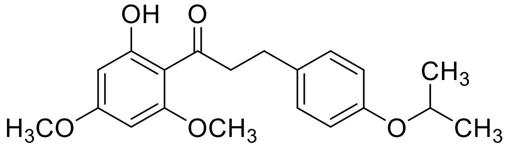

The title compound was obtained as a white solid (94%).
m.p. 73-74 °C (MeOH);
Rf = 0.23 (10% EtOAc/hexane);

IR (film) 2932, 1616, 1585, 1267, 1202, 1155, 1112, 952, 820 cm^{-1};
^{1}H NMR (500 MHz, $CDCl_3$) δ 14.05 (1H, s, O**H**), 7.12 (2H, d, *J* = 6.5 Hz, Ar**H**), 6.81 (2H, d, *J* = 6.9 Hz, Ar**H**), 6.06 (1H, d, *J* = 1.3 Hz, Ar**H**), 5.91 (1H, d, *J* = 1.5 Hz, Ar**H**), 3.82 (3H, s, OC**H**$_3$), 3.81 (3H, s, OC**H**$_3$), 3.29 – 3.25 (2H, m, C**H**$_2$), 2.93 – 2.90 (2H, m, C**H**$_2$), 1.61 (1H, s, C**H**), 1.32 (3H, s, C**H**$_3$), 1.31 (3H, s, C**H**$_3$).
^{13}C NMR (125.8 MHz, $CDCl_3$) δ 204.8, 167.8, 166.0, 162.8, 156.2, 133.6, 129.4 (2 × CH), 115.9 (2 × CH), 105.8, 93.7, 90.9, 70.0, 55.7, 55.6, 46.1, 29.9, 22.2 (2 × CH_3).
HRMS (ESI) Exact mass calcd for $C_{20}H_{25}O_5$ $[M+H]^+$: 345.1702, found 345.1692.

3-(4-(dimethylamino)phenyl)-1-(2-hydroxy-4,6-dimethoxyphenyl)propan-1-one (23)

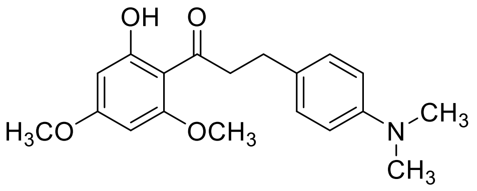

The title compound was obtained as a white solid (93%).
m.p. 125-126 °C (MeOH);
Rf = 0.28 (10% EtOAc/hexane);
IR (film) 2924, 1614, 1583, 1267, 1213, 1163, 1109, 970, 810 cm^{-1};
^{1}H NMR (500 MHz, $CDCl_3$) δ 14.09 (1H, s, O**H**), 7.12 (2H, d, *J* = 6.7 Hz, Ar**H**), 6.71 (2H, d, *J* = 6.9 Hz, Ar**H**), 6.06 (1H, d, *J* = 1.5 Hz, Ar**H**), 5.91 (1H, d, *J* = 1.5 Hz, Ar**H**), 3.83 (3H, s, OC**H**$_3$), 3.81 (3H, s, OC**H**$_3$), 3.29 – 3.24 (2H, t, C**H**$_2$), 2.91 (6H, s, 2 × C**H**$_3$), 2.88 (2H, d, *J* = 6.2 Hz, C**H**$_2$).
^{13}C NMR (125.8 MHz, $CDCl_3$) δ 205.1, 167.8, 166.0, 162.8, 149.2, 129.8, 129.1 (2 × CH), 113.1 (2 × CH), 105.8, 93.7, 90.9, 55.7, 55.6, 46.3, 41.0 (2 × CH_3), 29.9.
HRMS (ESI) Exact mass calcd for $C_{19}H_{24}NO_4$ $[M+H]^+$: 330.1705, found 330.1702.

3-(4-hydroxy-3-methoxyphenyl)-1-(2-hydroxy-4,6-dimethoxyphenyl)propan-1-one (24)

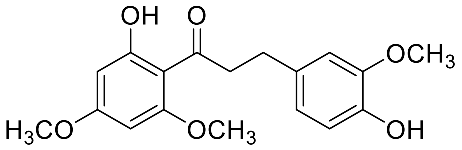

The title compound was obtained as a white solid (90%).
m.p. 121-122 °C (MeOH);
Rf = 0.33 (10% EtOAc/hexane);
IR (film) 2922, 1614, 1583, 1267, 1205, 1155, 1111, 970, 819 cm^{-1};
^{1}H NMR (500 MHz, $CDCl_3$) δ 14.03 (1H, s, O**H**), 6.83 (1H, d, *J* = 6.4 Hz, Ar**H**), 6.71 (1H, d, *J* = 6.6 Hz, Ar**H**), 6.06 (1H, d, *J* = 1.6 Hz, Ar**H**), 5.91 (1H, d, *J* = 1.3 Hz, Ar**H**), 5.51 (1H, s, Ar**H**), 3.86 (3H, s, OC**H**$_3$), 3.82 (3H, s, OC**H**$_3$), 3.81 (3H, s, OC**H**$_3$), 3.29 – 3.25 (2H, m, C**H**$_2$), 2.91 (2H, t, *J* = 6.2 Hz, C**H**$_2$).
^{13}C NMR (125.8 MHz, $CDCl_3$) δ 204.7, 167.7, 166.0, 162.8, 146.4, 143.8, 133.7, 120.9, 114.3, 111.3, 105.8, 93.7, 90.9, 55.9, 55.7, 55.7, 46.3, 30.5.
HRMS (ESI) Exact mass calcd for $C_{18}H_{21}O_6$ $[M+H]^+$: 333.1338, found 333.1332.

1-(2-hydroxy-4,6-dimethoxyphenyl)-3-(2,4,6-trimethoxyphenyl)propan-1-one (25)

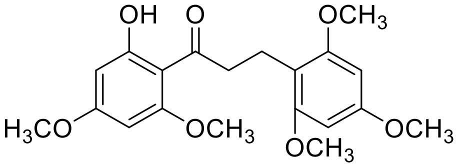

The title compound was obtained as a white solid (94%).
m.p. 133-134 °C (MeOH);
Rf = 0.08 (20% EtOAc/hexane);
IR (film) 2926, 1728, 1593, 1415, 1271, 1215, 1161, 1118, 948, 812 cm^{-1};
^{1}H NMR (500 MHz, $CDCl_3$) δ 14.18 (1H, s, O**H**), 6.13 (2H, s, Ar**H**), 6.06 (1H, d, *J* = 1.7 Hz, Ar**H**), 5.89 (1H, d, *J* = 1.7 Hz, Ar**H**), 3.81 (6H, d, *J* = 1.6 Hz, 2 × OC**H**$_3$), 3.78 (3H, s, OC**H**$_3$), 3.76 (6H, s, 2 × OC**H**$_3$), 3.14 – 3.10 (2H, m, C**H**$_2$), 2.96 – 2.92 (2H, m, C**H**$_2$).
^{13}C NMR (125.8 MHz, $CDCl_3$) δ 206.3, 167.6, 165.7, 162.9, 159.5, 159.0, 131.0, 128.9, 110.2, 106.1, 93.6, 90.7, 90.5, 55.7 (2 × CH_3), 55.5 (2 × CH_3), 55.4, 43.9, 18.3.
HRMS (ESI) Exact mass calcd for $C_{20}H_{25}O_7$ $[M+H]^+$: 377.1600, found 377.1596.

1-(2-hydroxy-4,6-dimethoxyphenyl)-3-(1-methyl-1H-pyrrol-2-yl)propan-1-one (26)

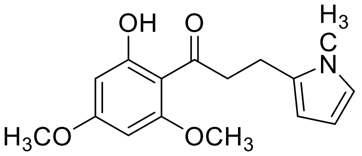

The title compound was obtained as a white solid (89%).
m.p. 119-120 °C (MeOH);
Rf = 0.40 (10% EtOAc/hexane);
IR (film) 2926, 1728, 1597, 1417, 1274, 1213, 1159, 1120, 939, 813 cm^{-1};
^{1}H NMR (500 MHz, $CDCl_3$) δ 13.99 (1H, s, O**H**), 6.57 (1H, s, Ar**H**), 6.08 (1H, d, *J* = 1.6 Hz, Ar**H**), 6.06 (1H, d, *J* = 2.5 Hz, Ar**H**), 5.94 (1H, d, *J* = 1.5 Hz, Ar**H**), 5.91 (1H, s, Ar**H**), 3.87 (3H, s, OC**H**$_3$), 3.83 (3H, s, OC**H**$_3$), 3.59 (3H, s, C**H**$_3$), 3.39 – 3.35 (2H, m, C**H**$_2$), 2.94 – 2.90 (2H, m, C**H**$_2$).
^{13}C NMR (125.8 MHz, $CDCl_3$) δ 204.3, 167.7, 166.1, 162.9, 132.7, 121.4, 106.6, 105.8, 105.3, 93.7, 91.0, 55.7, 55.6, 43.1, 33.7, 21.0.
HRMS (ESI) Exact mass calcd for $C_{16}H_{20}NO_4$ $[M+H]^+$: 290.1392, found 290.1386.

1-(2-hydroxy-4,6-dimethoxyphenyl)-3-(thiophen-2-yl)propan-1-one (27)

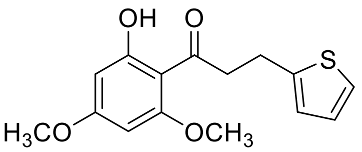

The title compound was obtained as a white solid (93%).
m.p. 85-86 °C (MeOH);
Rf = 0.60 (10% EtOAc/hexane);
IR (film) 2937, 1614, 1583, 1415, 1369, 1273, 1155, 1112, 968, 817 cm^{-1};

^{1}H NMR (500 MHz, $CDCl_3$) δ 13.96 (1H, s, O**H**), 7.12 (1H, d, *J* = 4.3 Hz, Ar**H**), 6.94 – 6.91 (1H, m, Ar**H**), 6.85 (1H, d, *J* = 2.5 Hz, Ar**H**), 6.07 (1H, d, *J* = 1.7 Hz, Ar**H**), 5.93 (1H, d, *J* = 1.8 Hz, Ar**H**), 3.85 (3H, s, OCH_3), 3.81 (3H, s, OCH_3), 3.40 – 3.36 (2H, m, CH_2), 3.23 – 3.19 (2H, m, CH_2).
^{13}C NMR (125.8 MHz, $CDCl_3$) δ 203.7, 167.7, 166.1, 162.8, 144.6, 126.8, 124.5, 123.3, 105.8, 93.8, 90.9, 55.7, 55.6, 46.0, 24.8.
HRMS (ESI) Exact mass calcd for $C_{15}H_{17}O_4S$ $[M+H]^+$: 293.0847, found 293.0843.

2) ^{1}H NMR and ^{13}C NMR spectra of dihydrochalcones (1–27)

1-(2-hydroxy-4,6-dimethoxyphenyl)-3-phenylpropan-1-one (1)

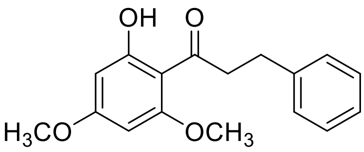

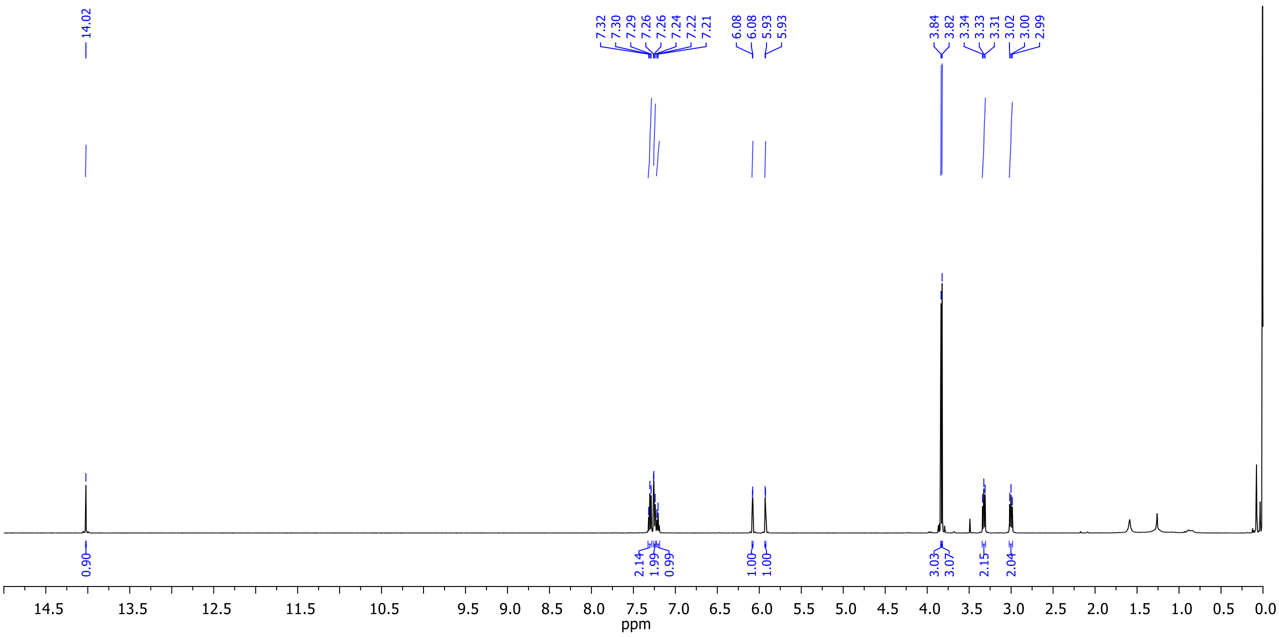

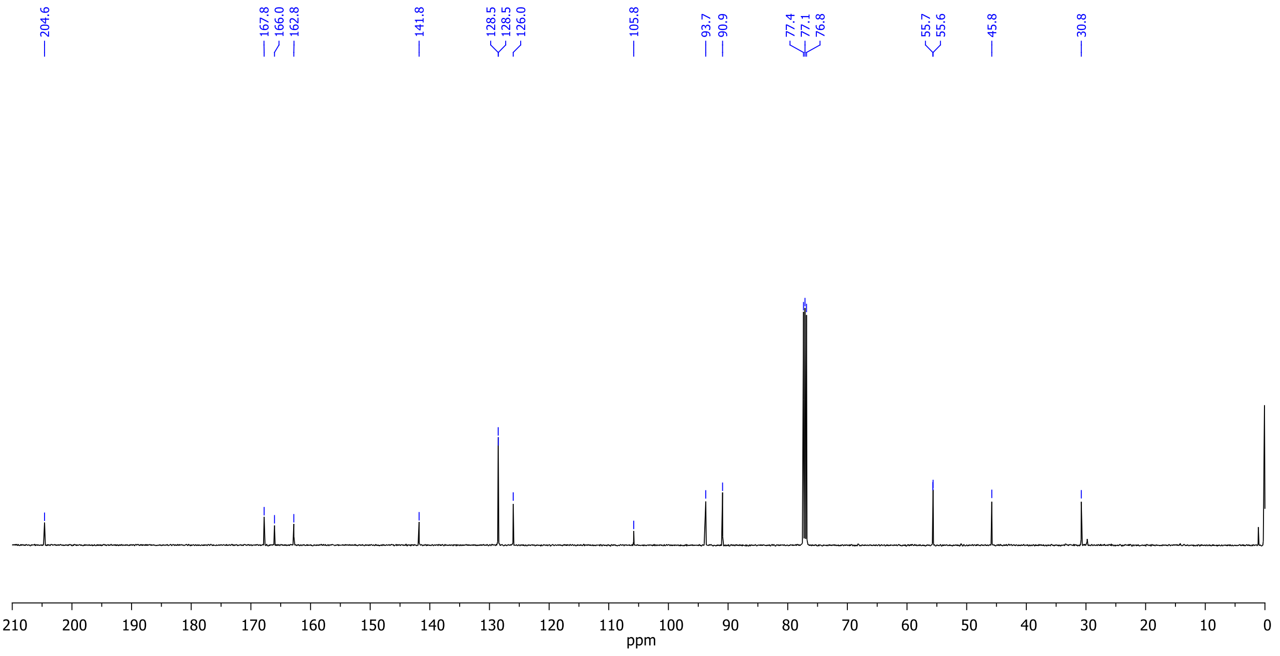

1-(2-hydroxy-4,6-dimethoxyphenyl)-3-(o-tolyl)propan-1-one (2)

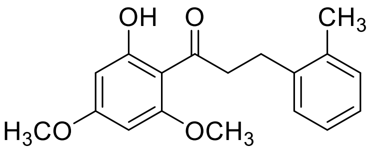

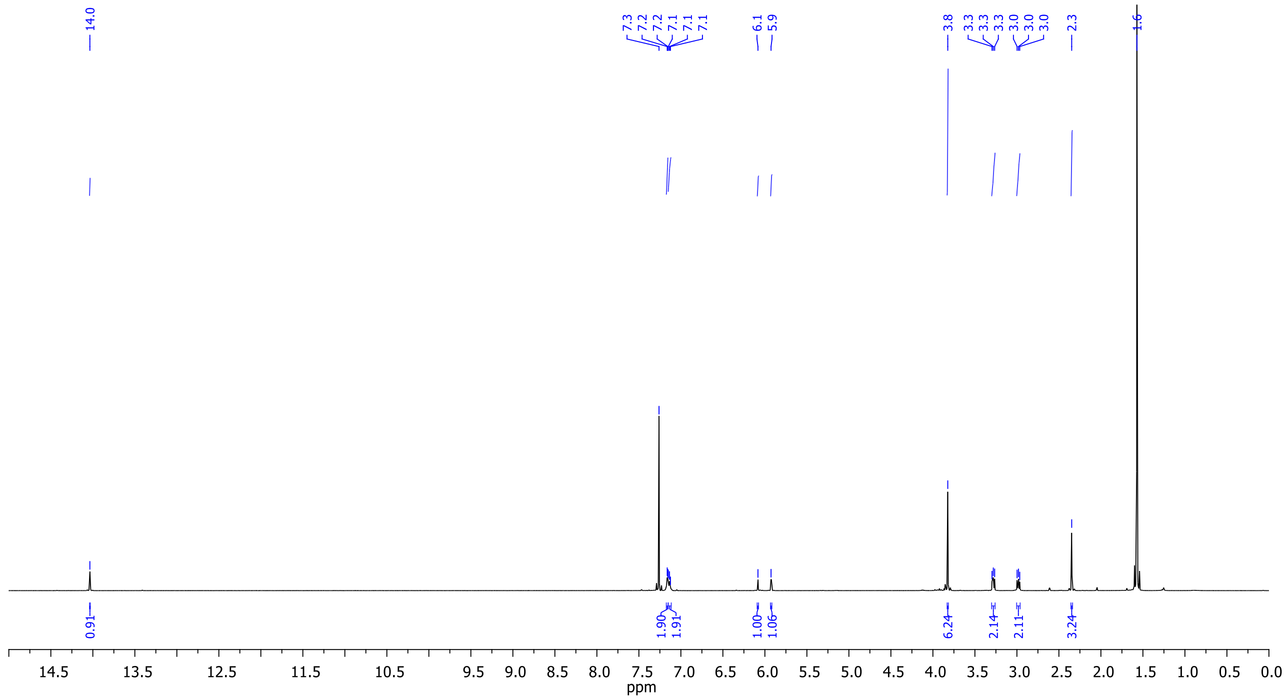

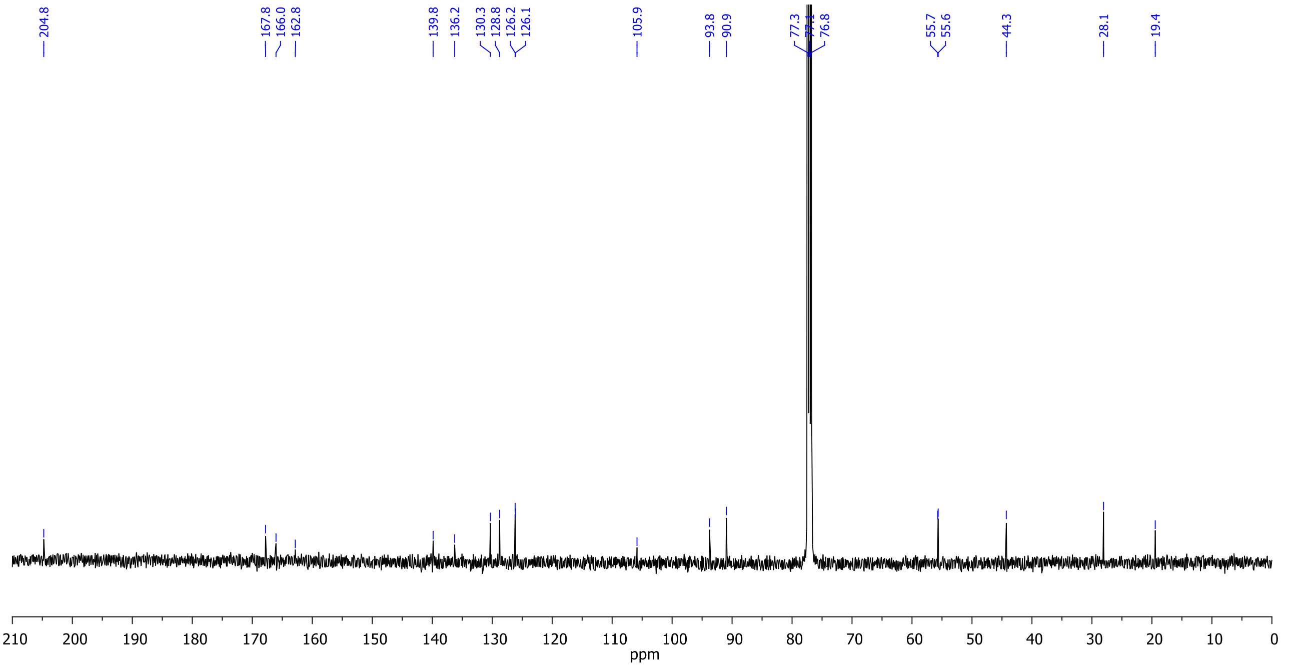

1-(2-hydroxy-4,6-dimethoxyphenyl)-3-(m-tolyl)propan-1-one (3)

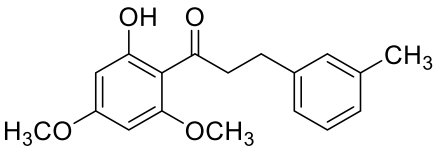

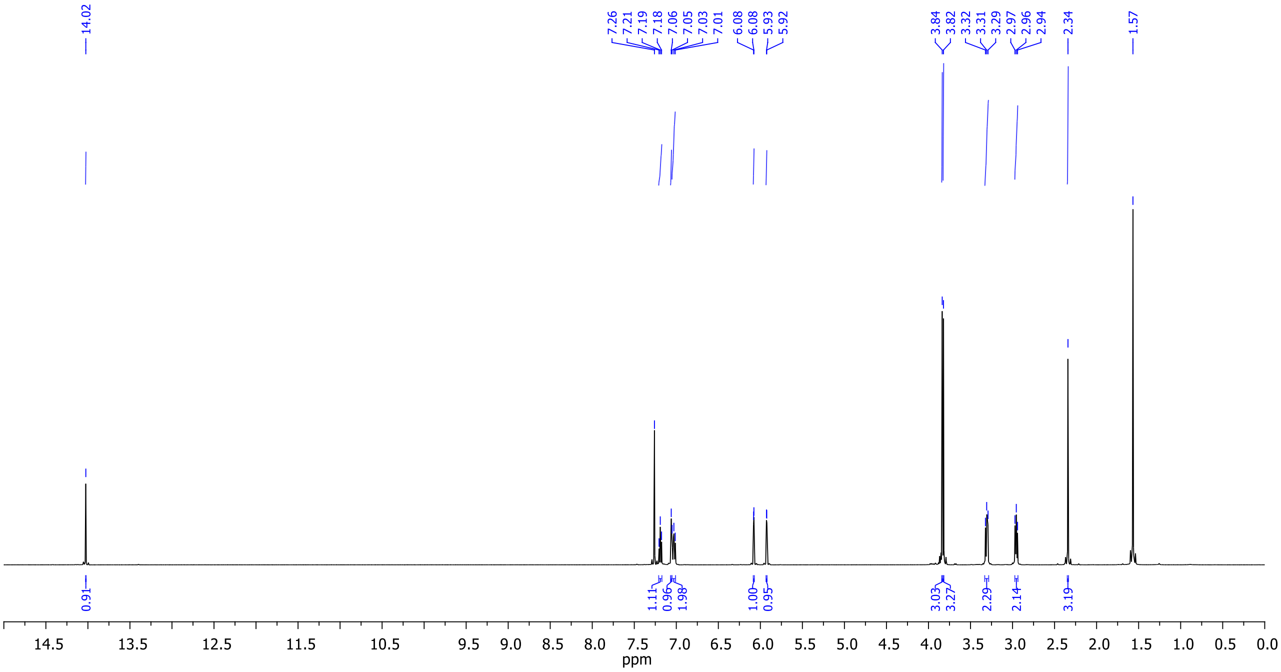

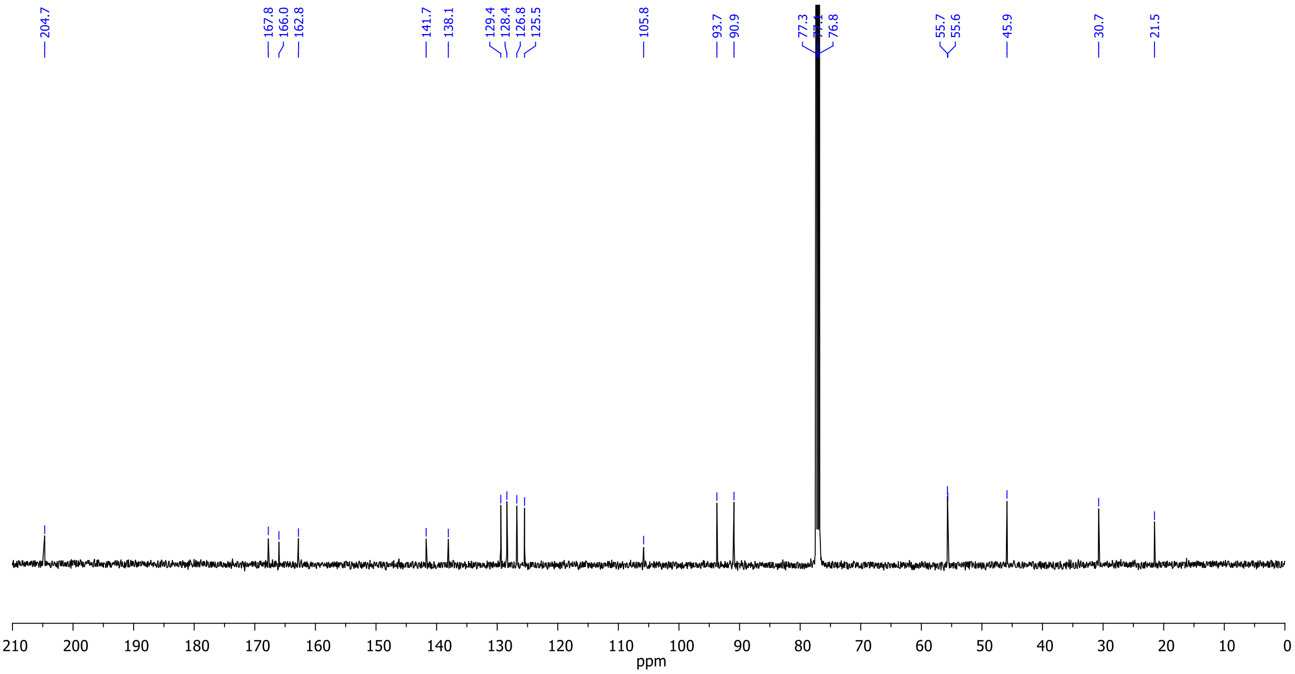

1-(2-hydroxy-4,6-dimethoxyphenyl)-3-(p-tolyl)propan-1-one (4)

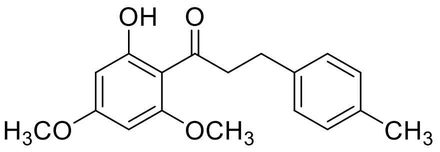

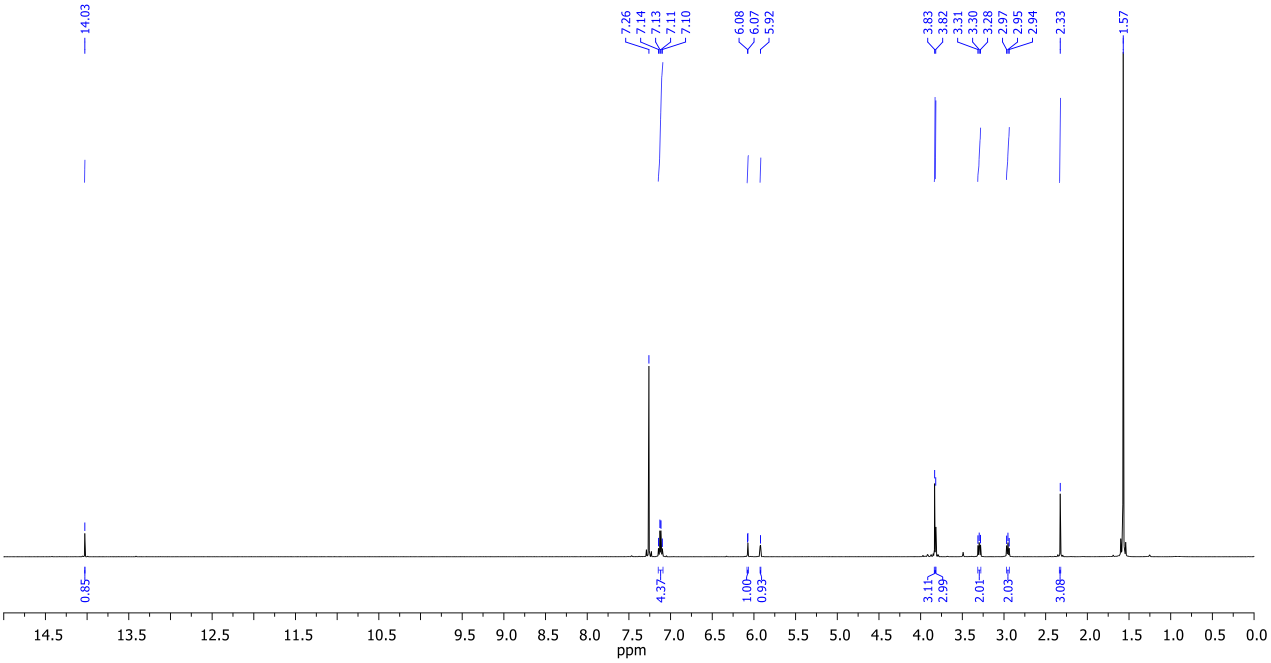

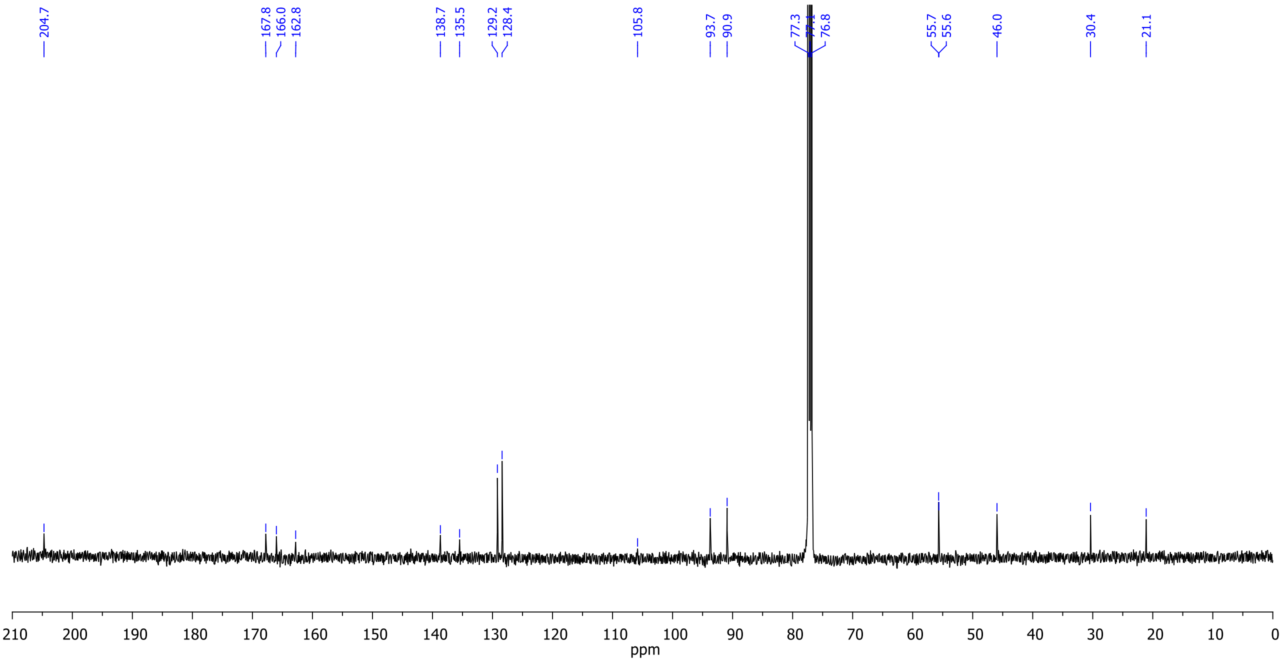

1-(2-hydroxy-4,6-dimethoxyphenyl)-3-(2-hydroxyphenyl)propan-1-one (5)

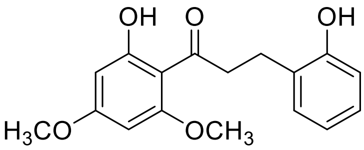

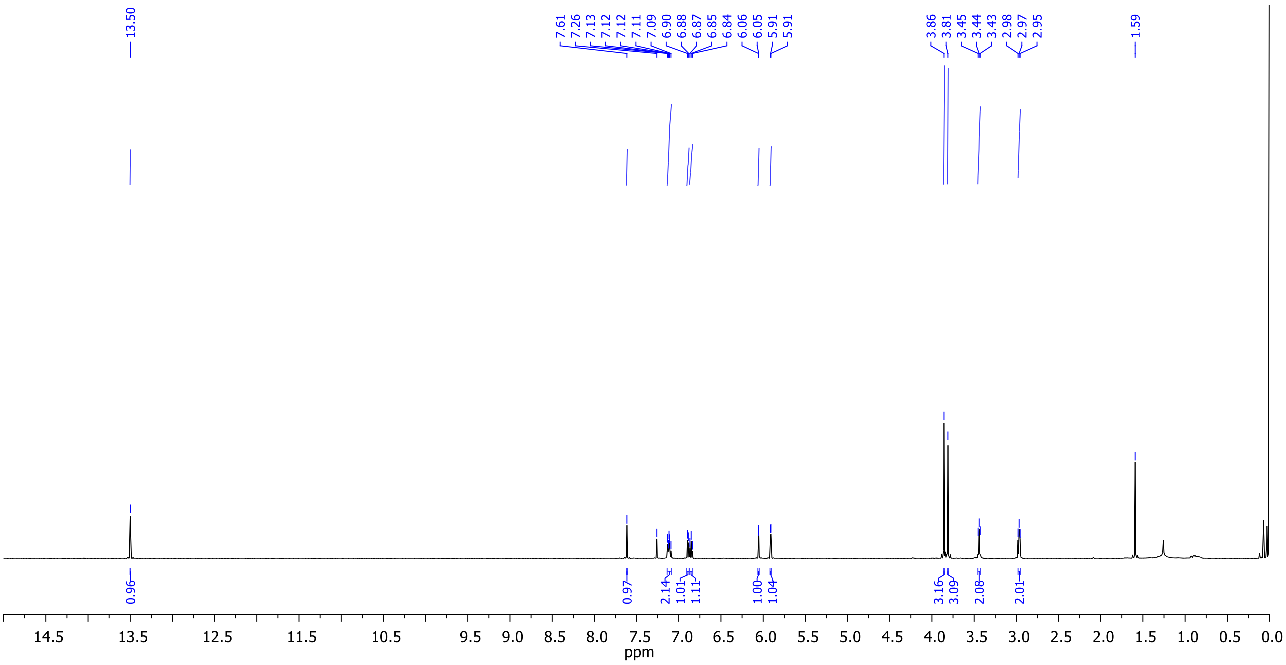

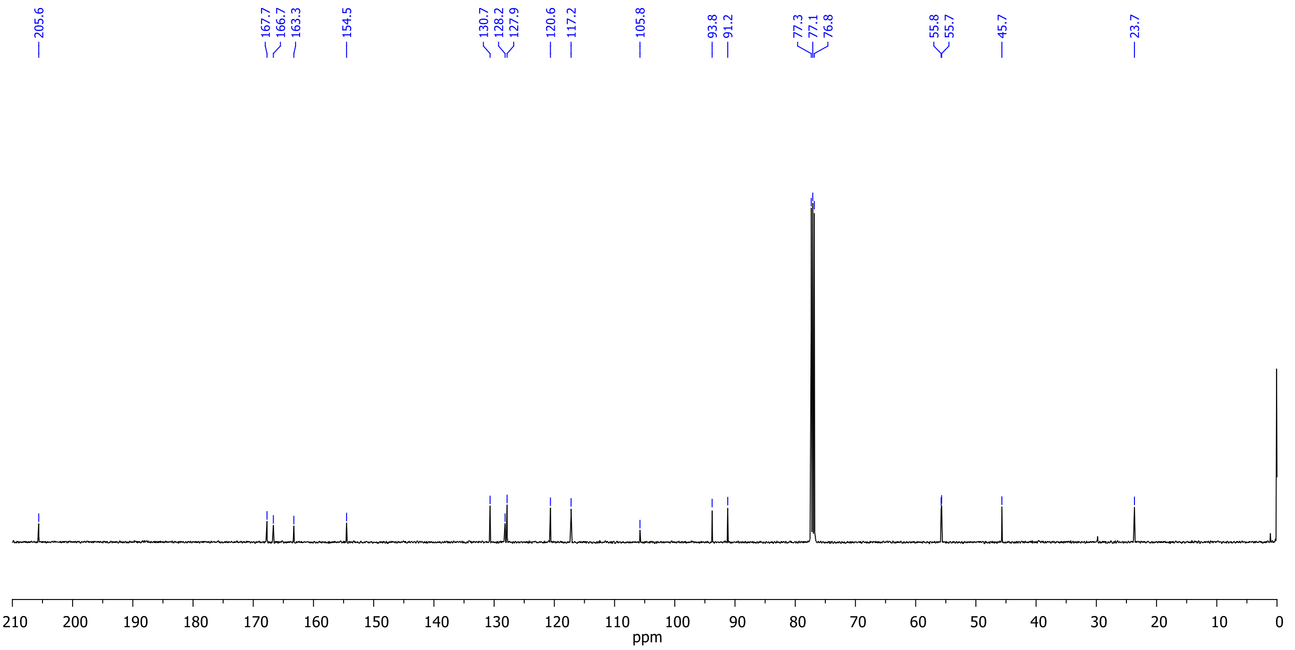

1-(2-hydroxy-4,6-dimethoxyphenyl)-3-(3-hydroxyphenyl)propan-1-one (6)

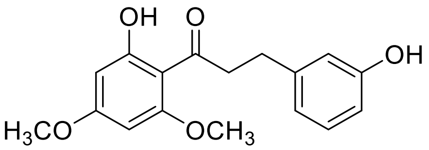

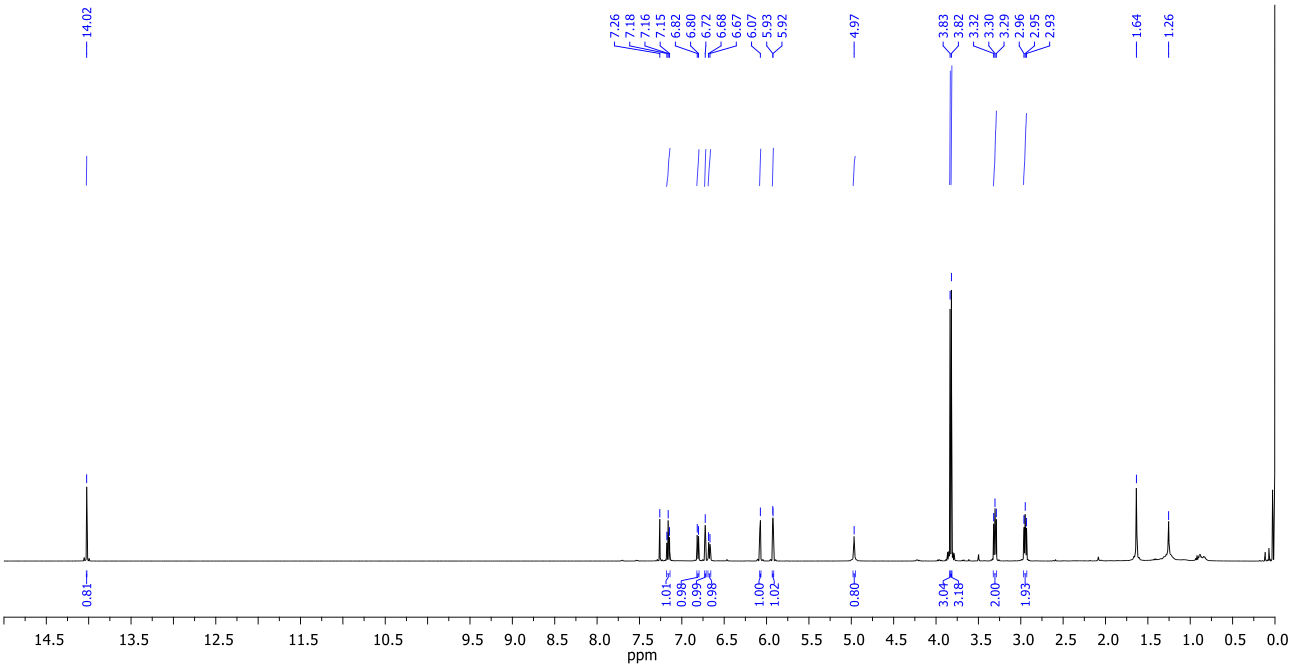

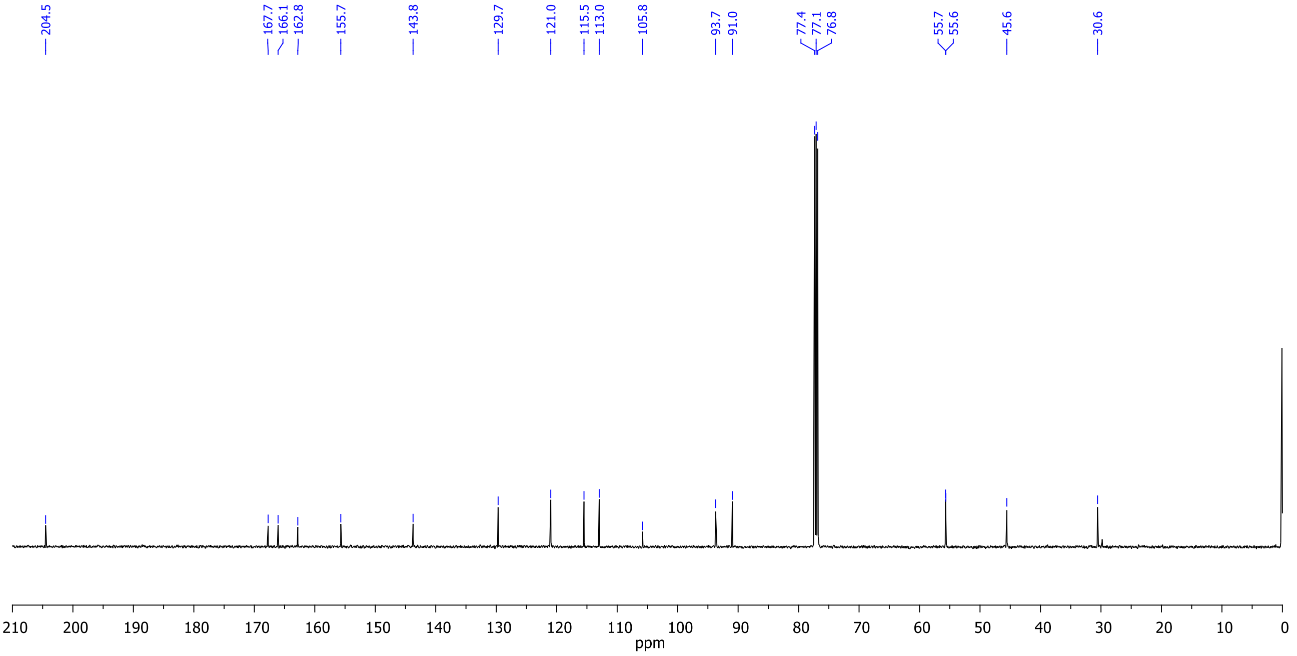

1-(2-hydroxy-4,6-dimethoxyphenyl)-3-(4-hydroxyphenyl)propan-1-one (7)

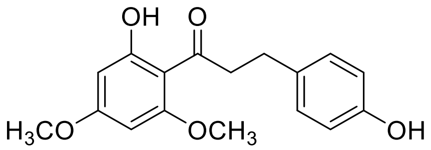

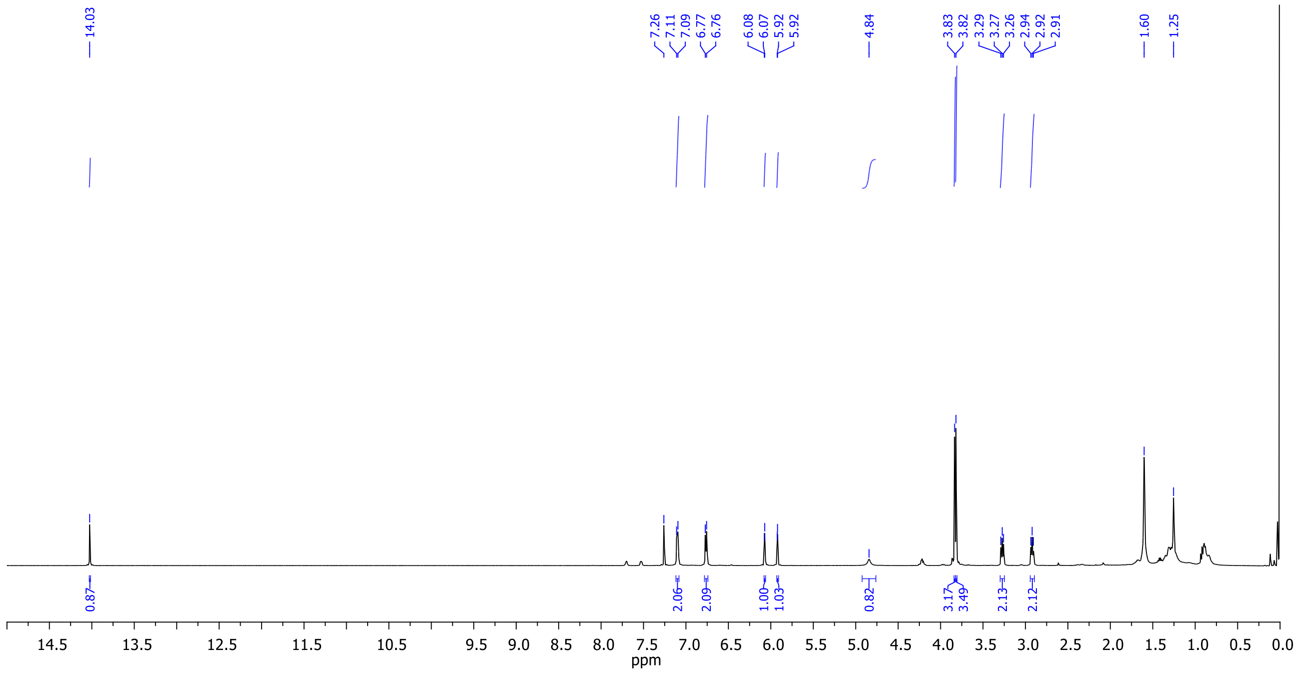

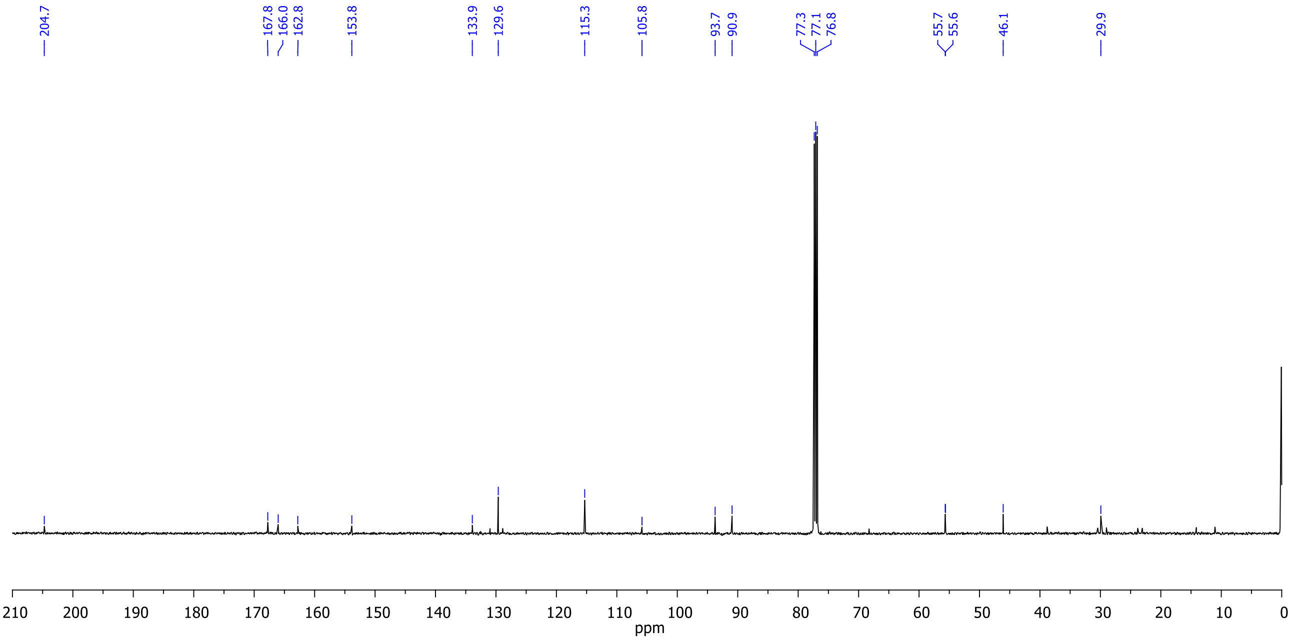

1-(2-hydroxy-4,6-dimethoxyphenyl)-3-(2-methoxyphenyl)propan-1-one (8)

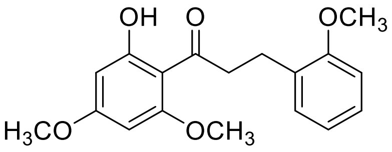

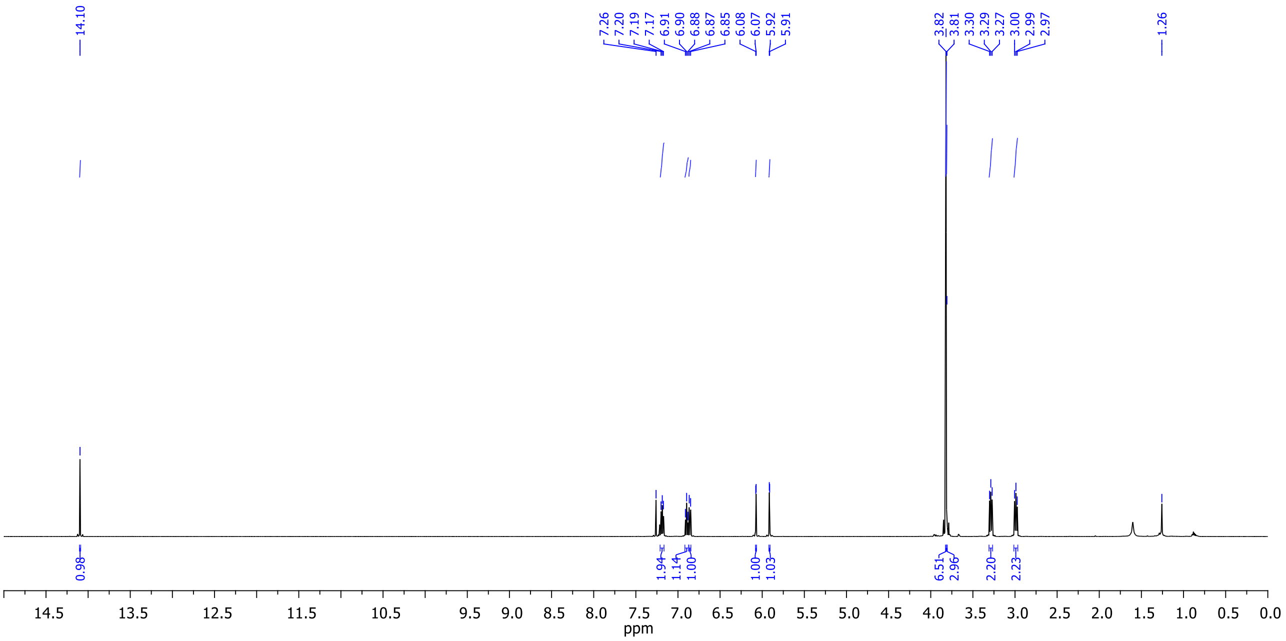

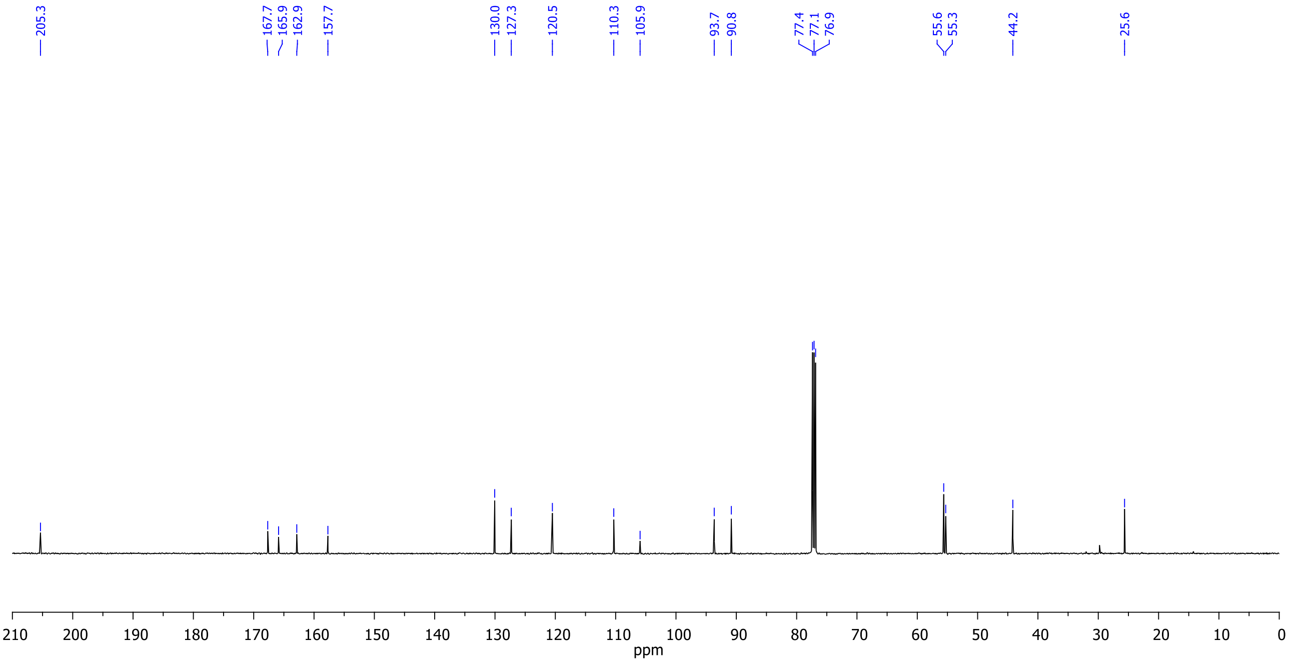

1-(2-hydroxy-4,6-dimethoxyphenyl)-3-(3-methoxyphenyl)propan-1-one (9)

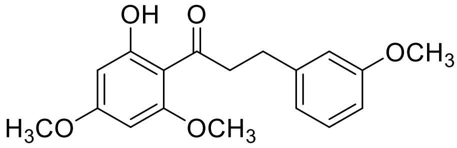

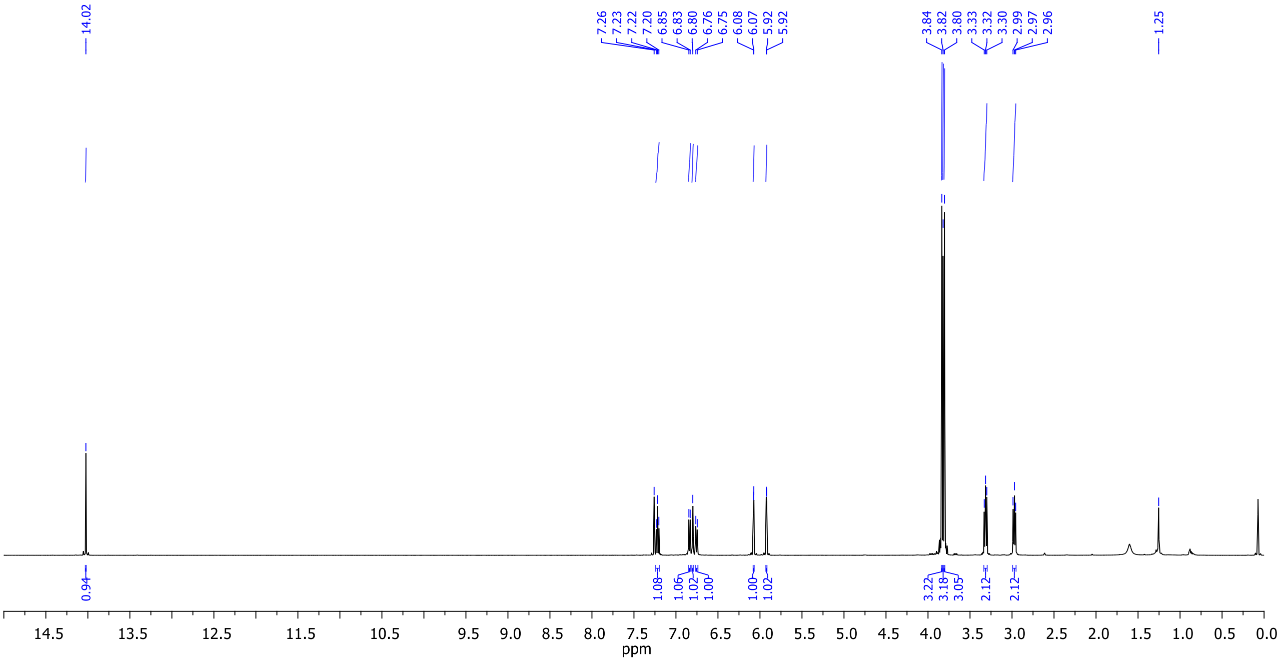

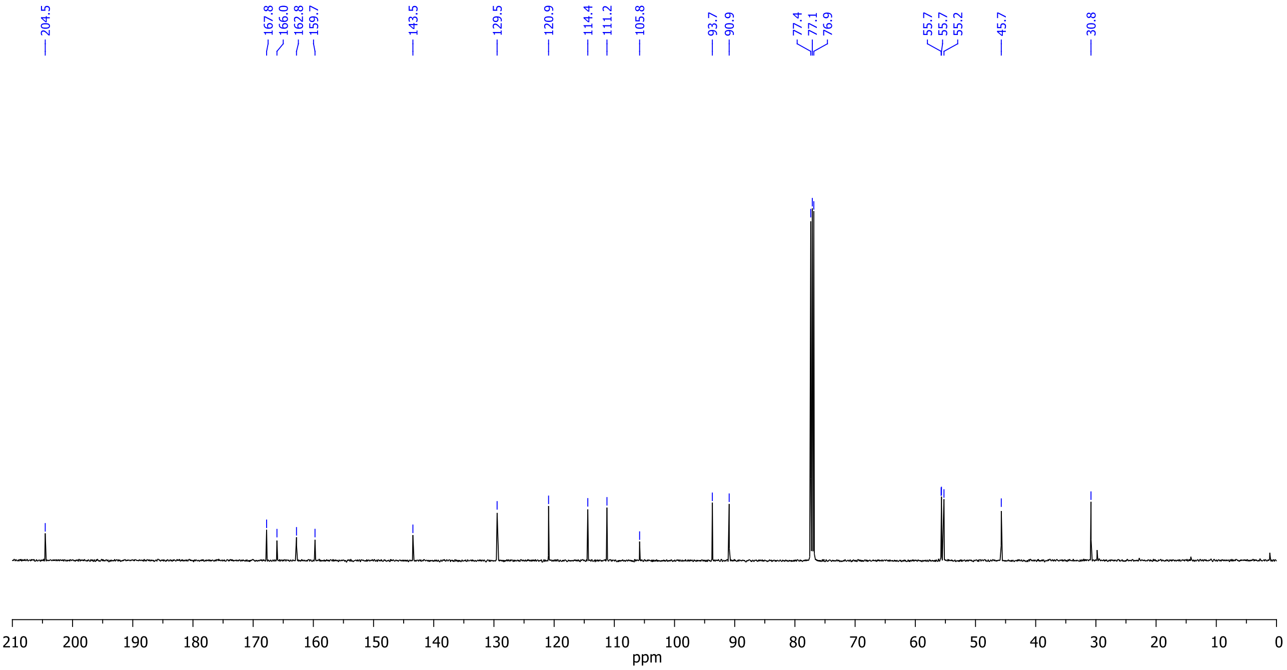

1-(2-hydroxy-4,6-dimethoxyphenyl)-3-(4-methoxyphenyl)propan-1-one (10)

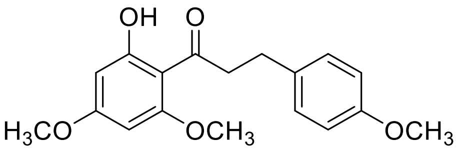

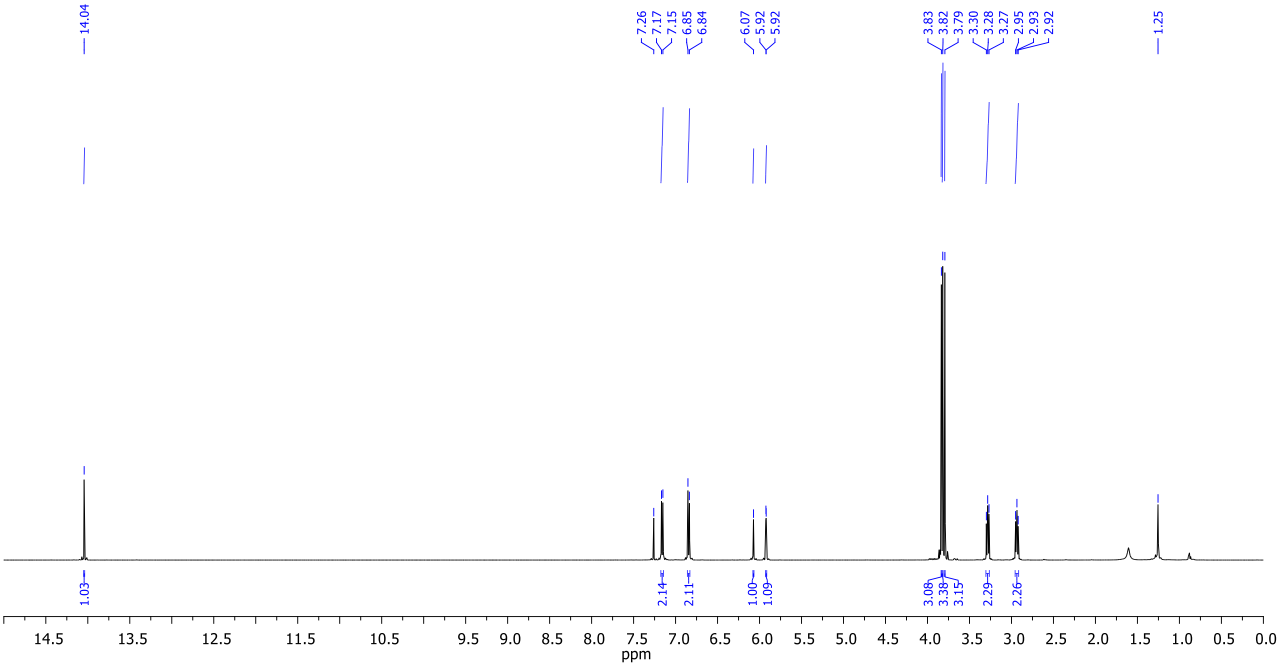

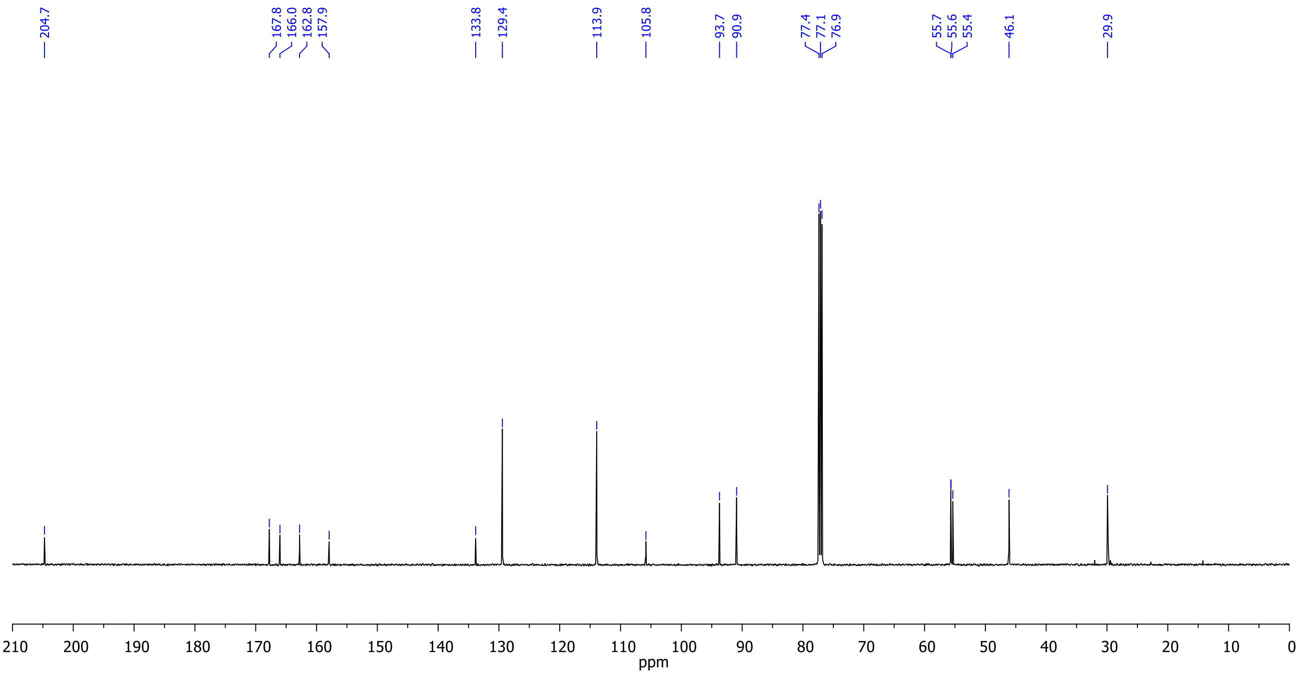

3-(2-fluorophenyl)-1-(2-hydroxy-4,6-dimethoxyphenyl)propan-1-one (11)

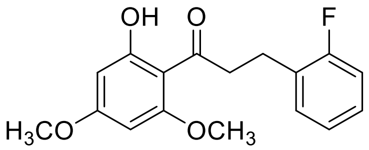

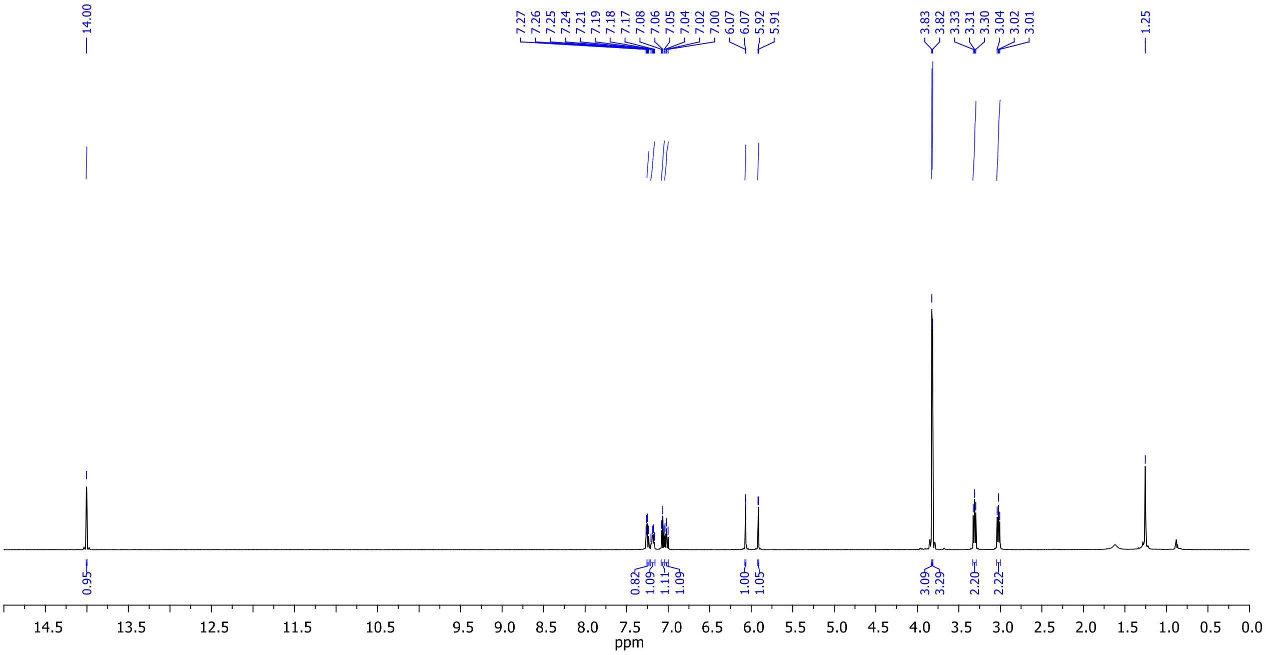

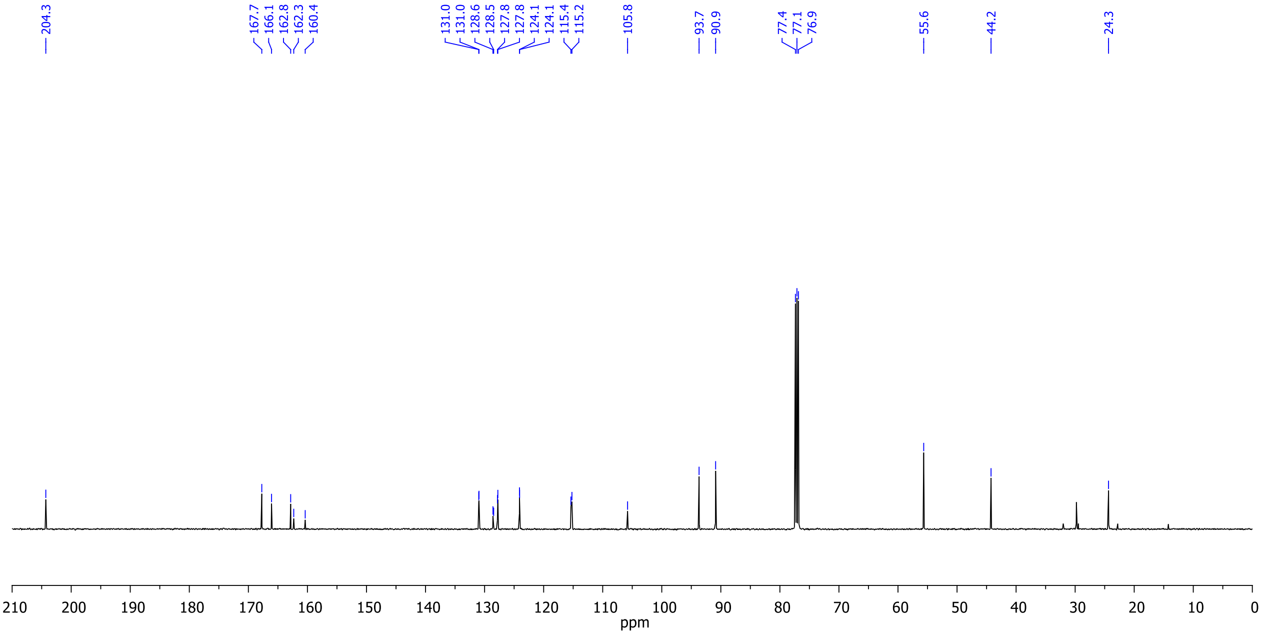

3-(3-fluorophenyl)-1-(2-hydroxy-4,6-dimethoxyphenyl)propan-1-one (12)

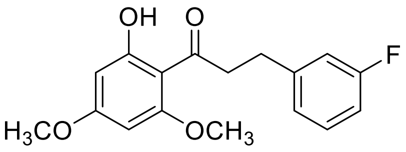

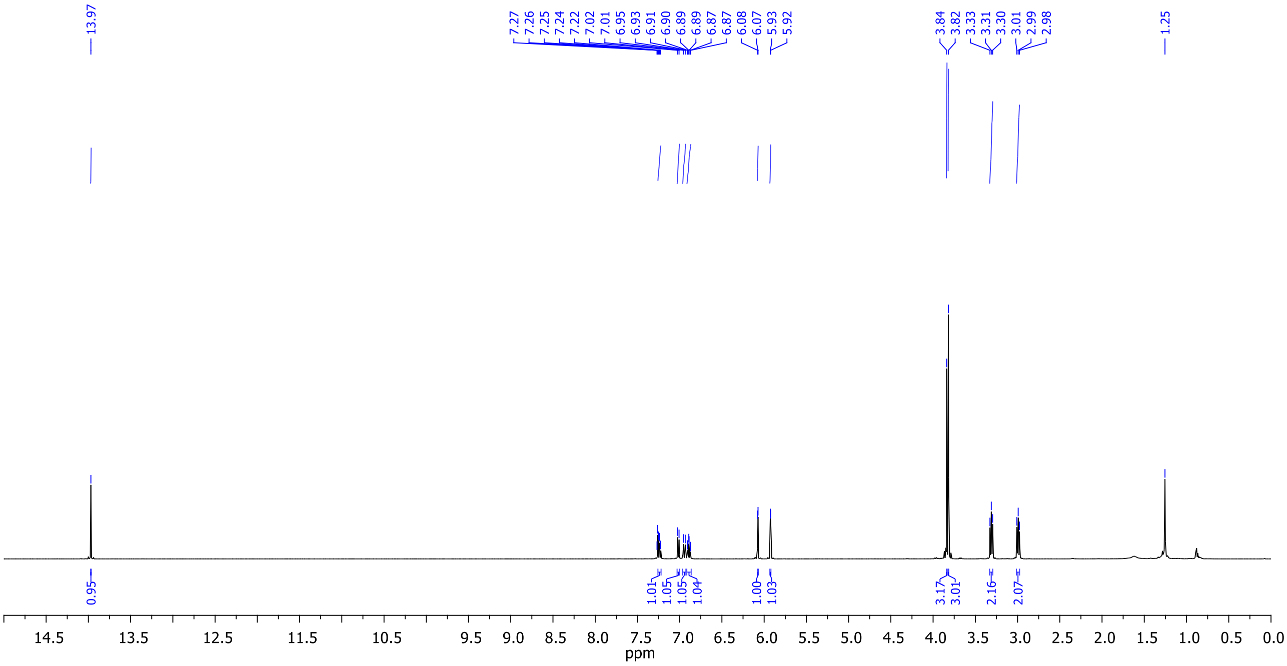

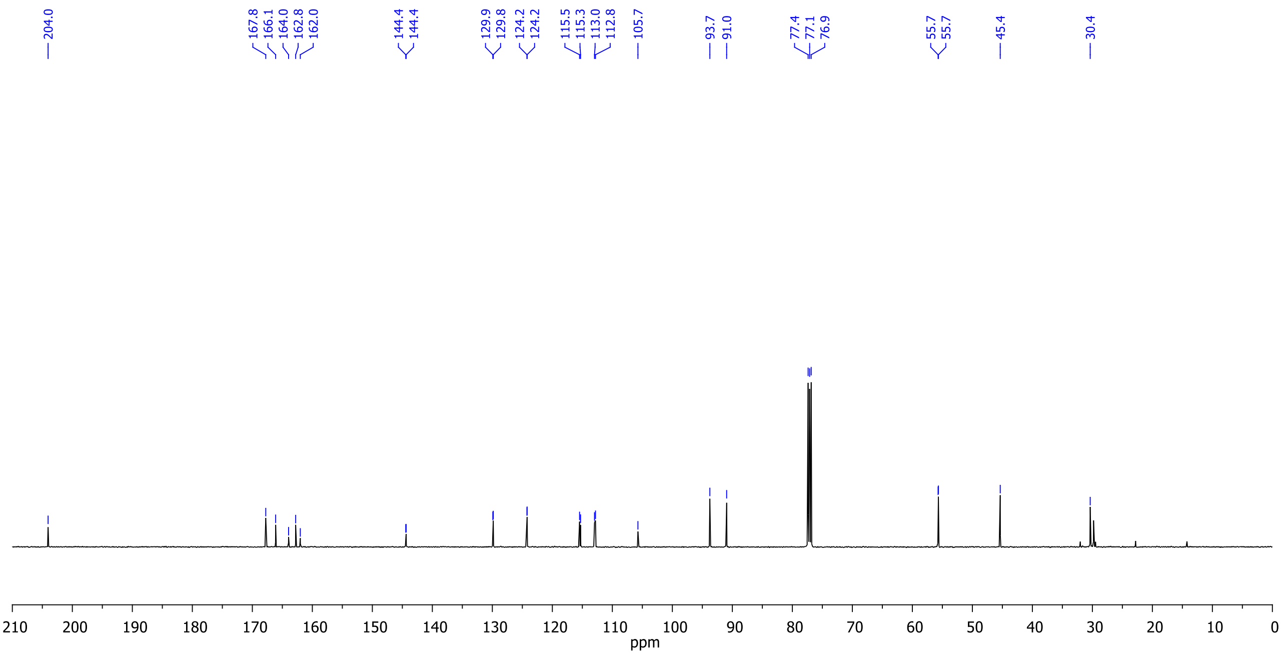

3-(4-fluorophenyl)-1-(2-hydroxy-4,6-dimethoxyphenyl)propan-1-one (13)

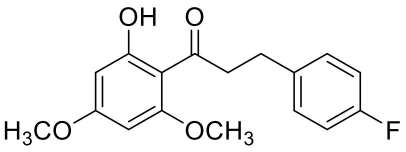

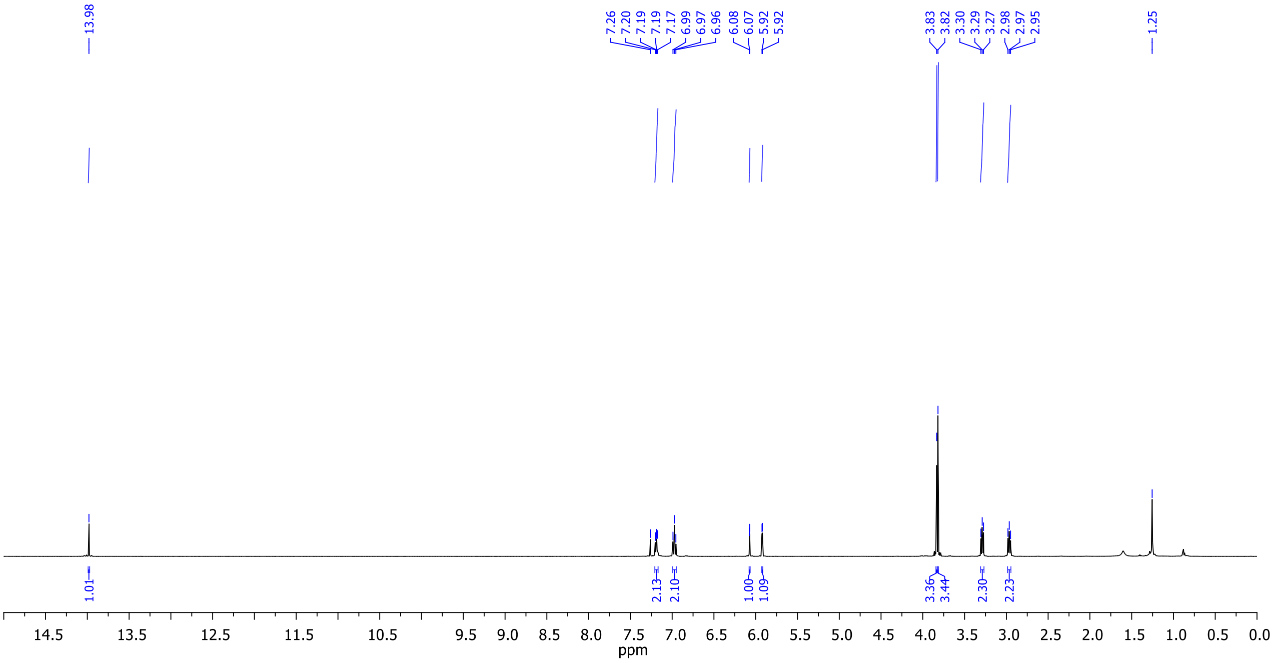

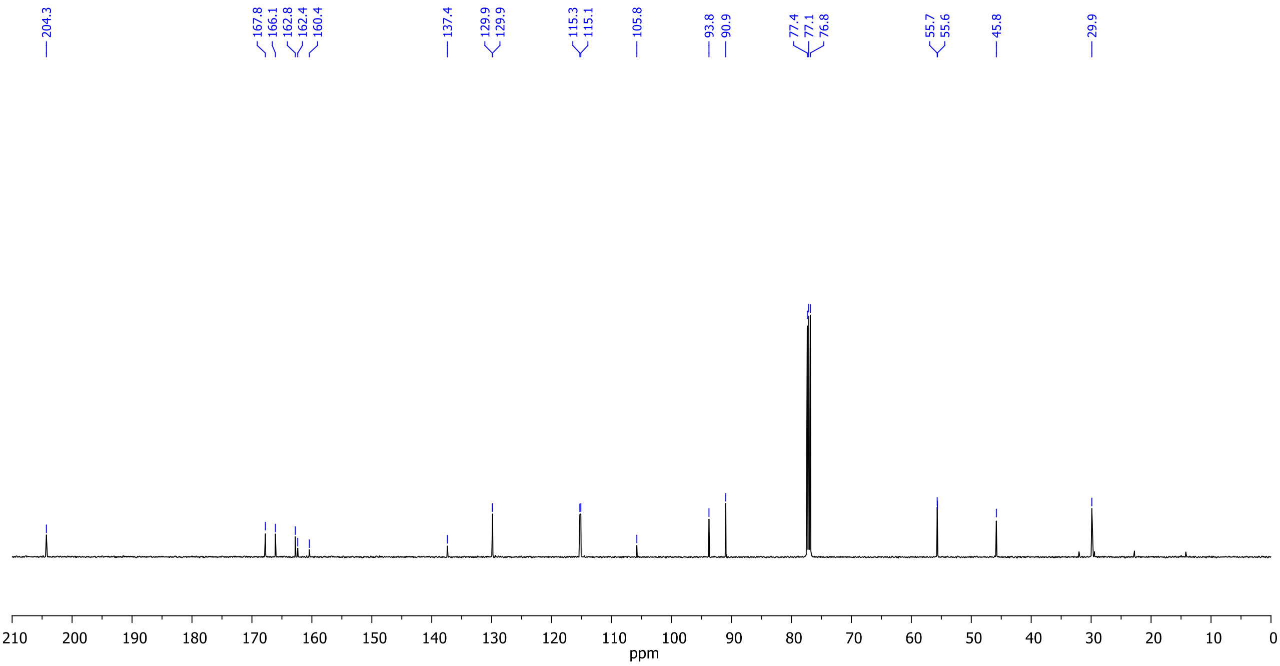

3-(2-chlorophenyl)-1-(2-hydroxy-4,6-dimethoxyphenyl)propan-1-one (14)

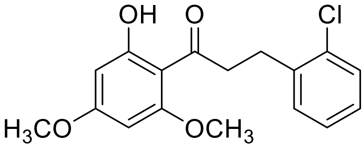

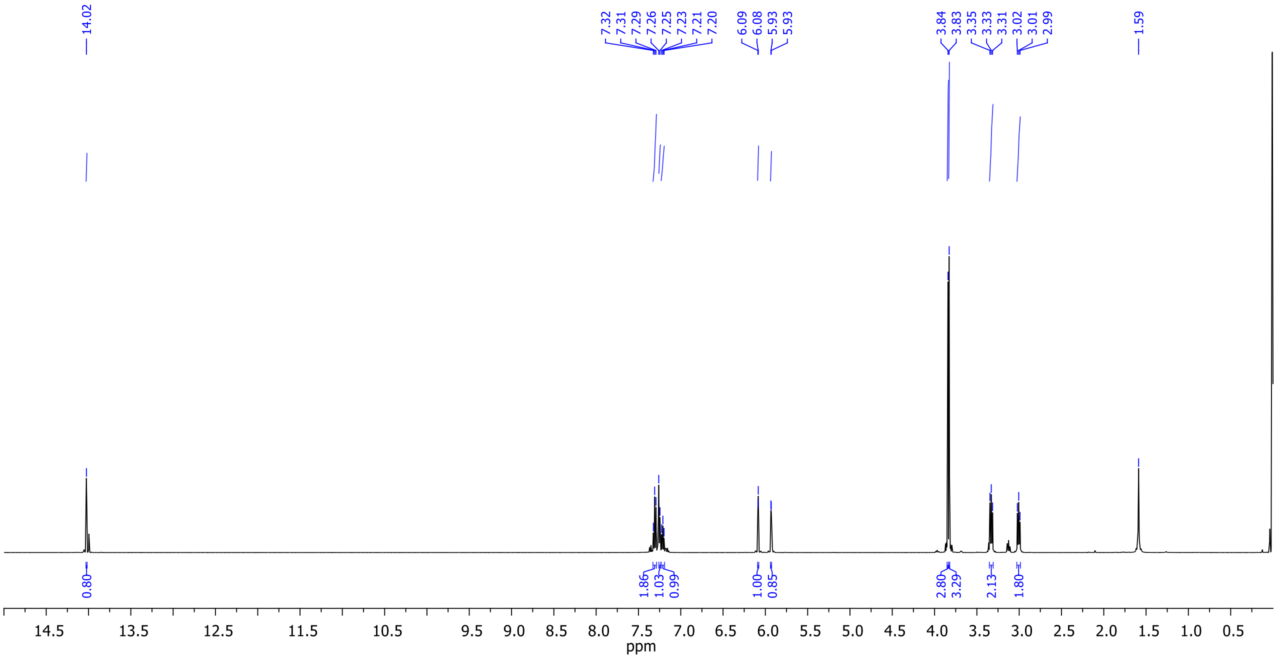

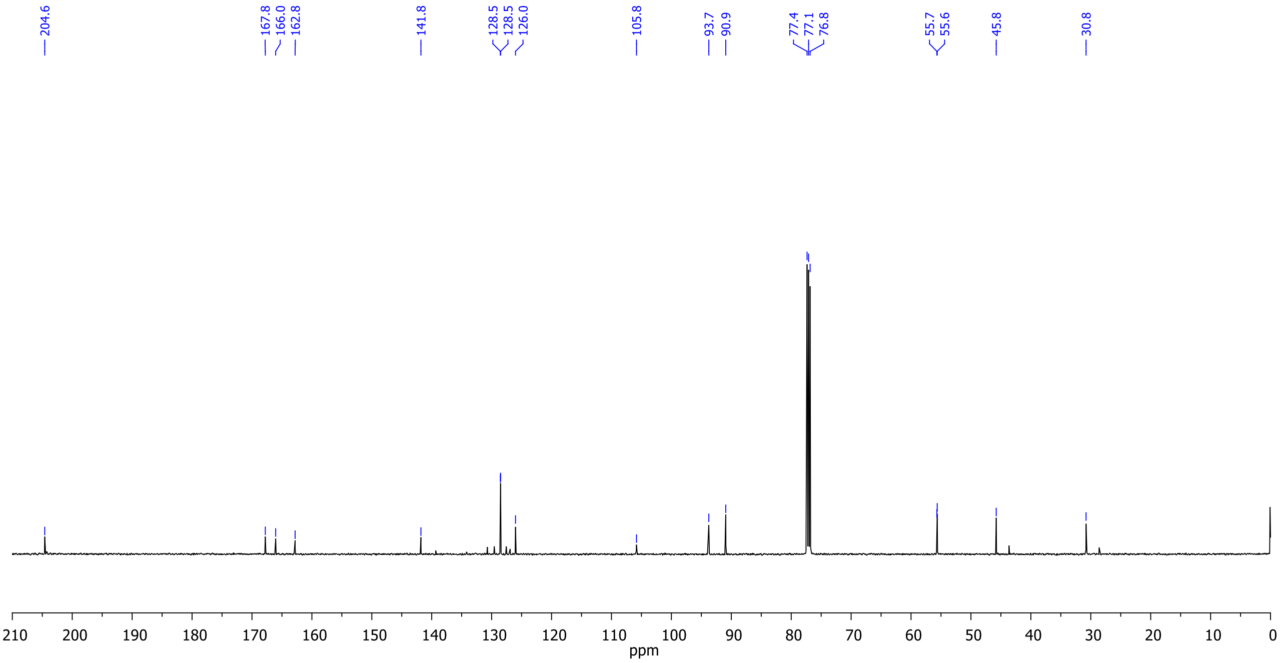

3-(3-chlorophenyl)-1-(2-hydroxy-4,6-dimethoxyphenyl)propan-1-one (15)

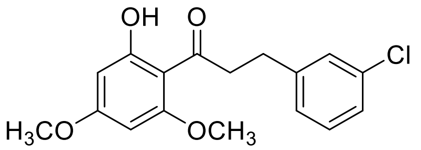

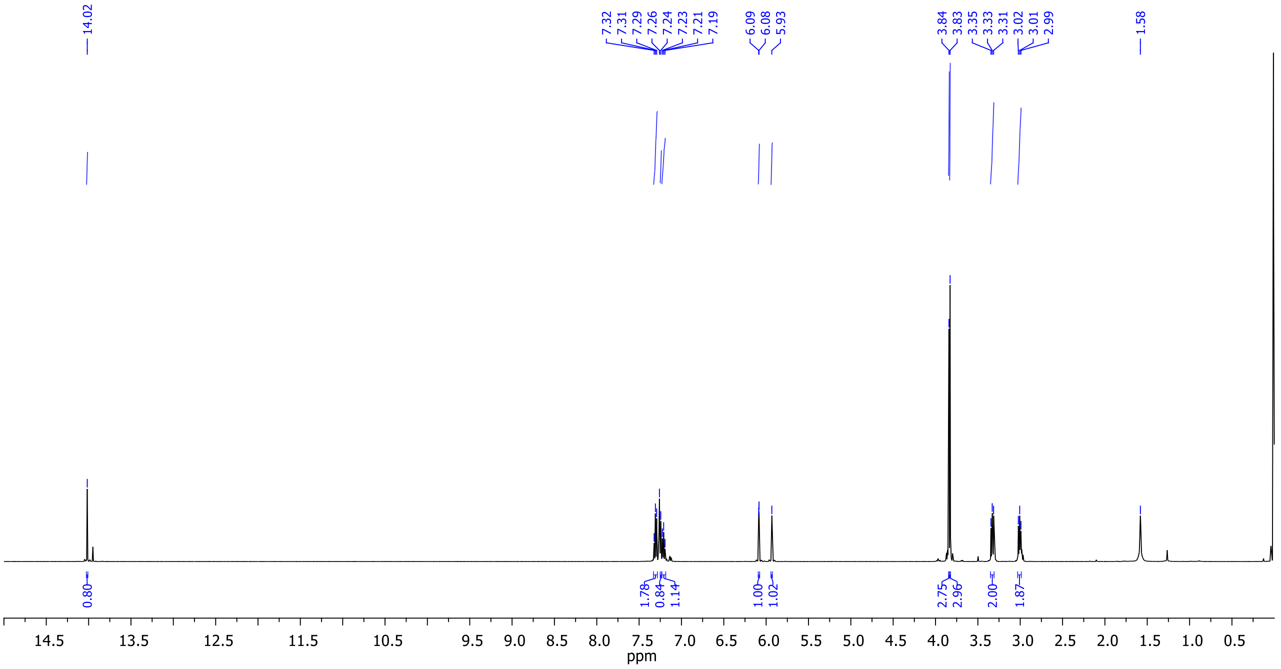

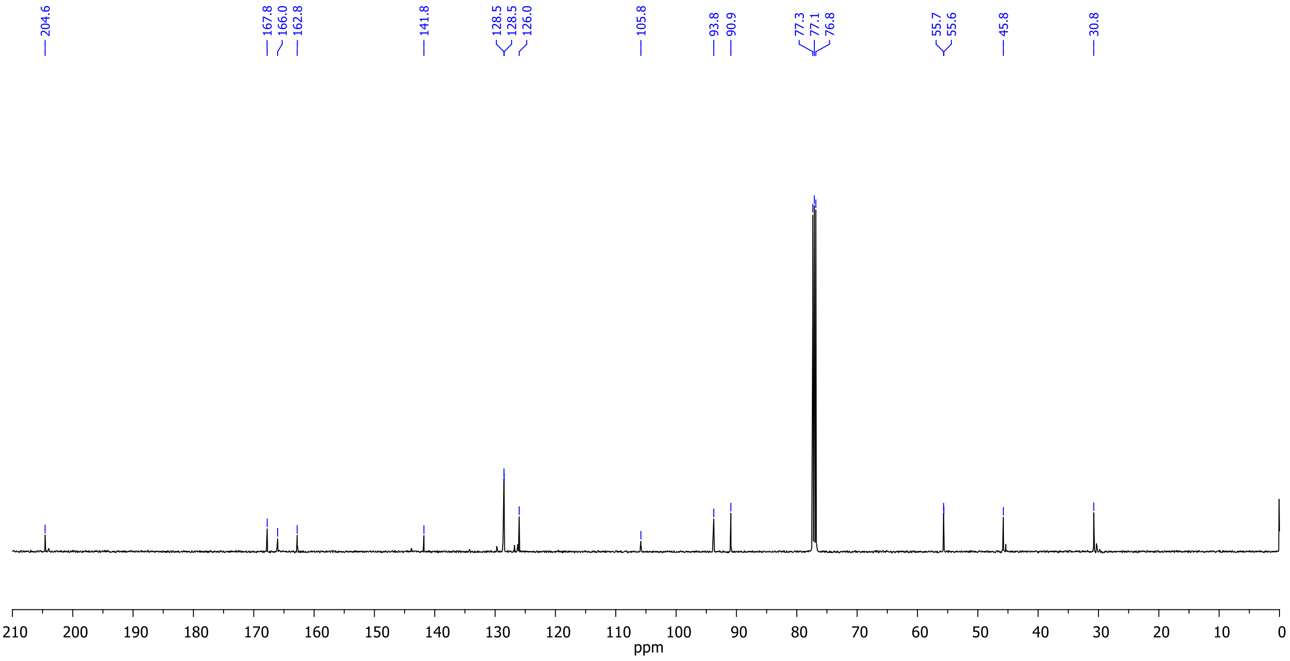

3-(4-chlorophenyl)-1-(2-hydroxy-4,6-dimethoxyphenyl)propan-1-one (16)

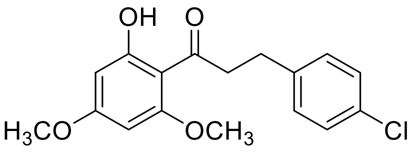

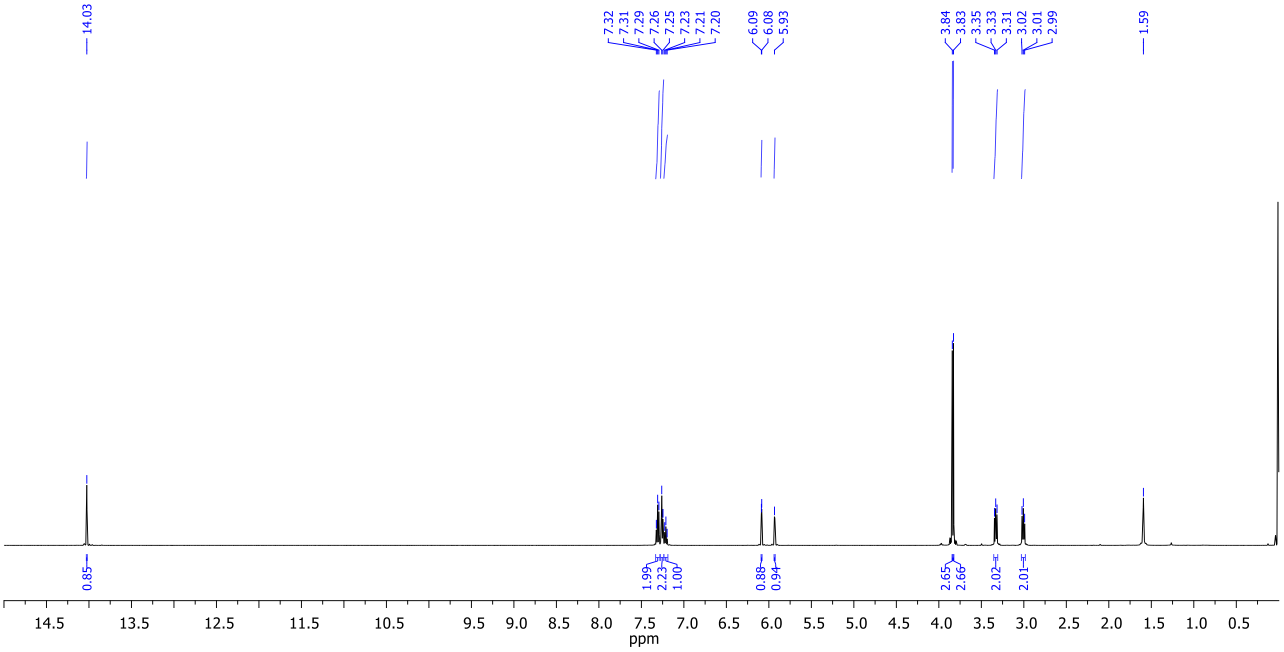

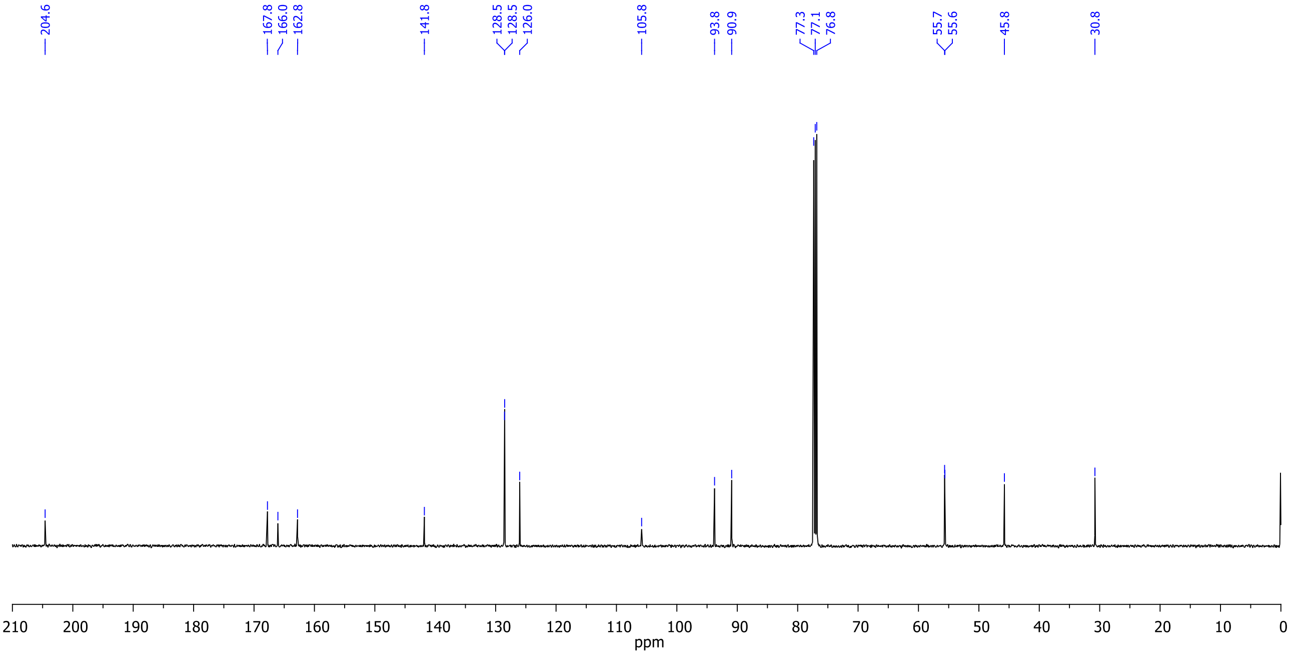

3-(2-bromophenyl)-1-(2-hydroxy-4,6-dimethoxyphenyl)propan-1-one (17)

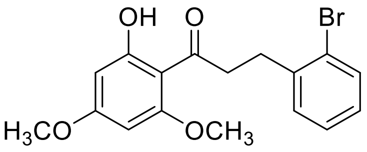

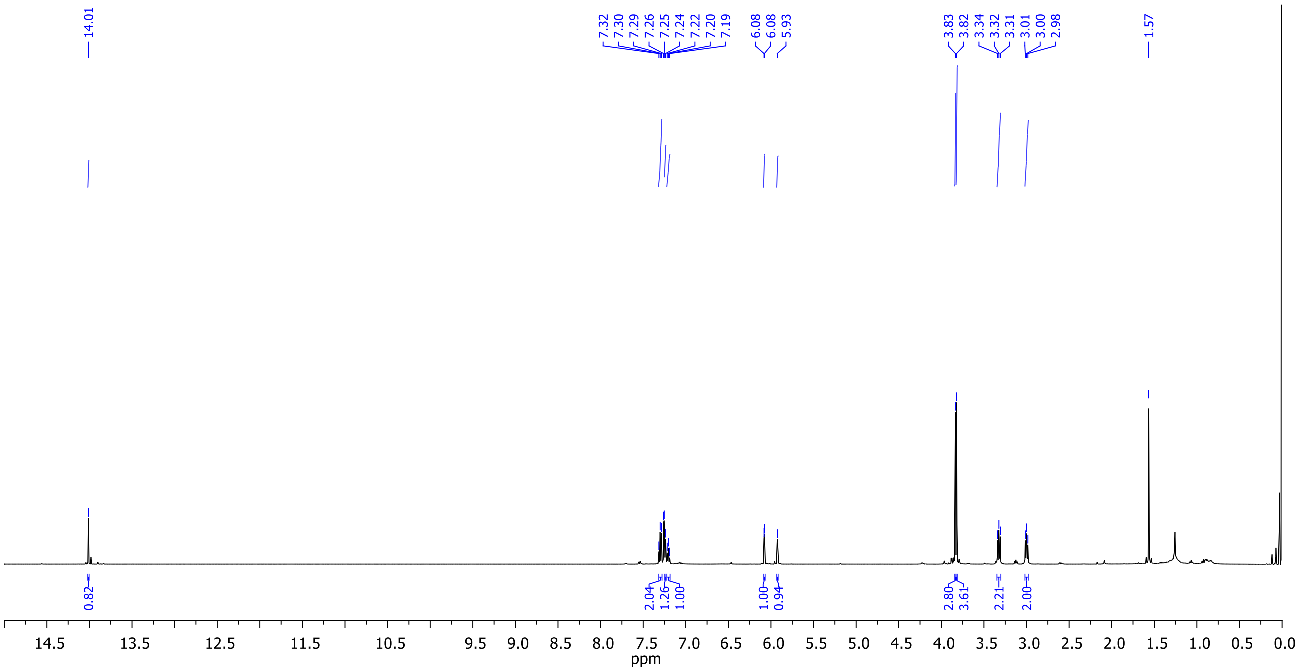

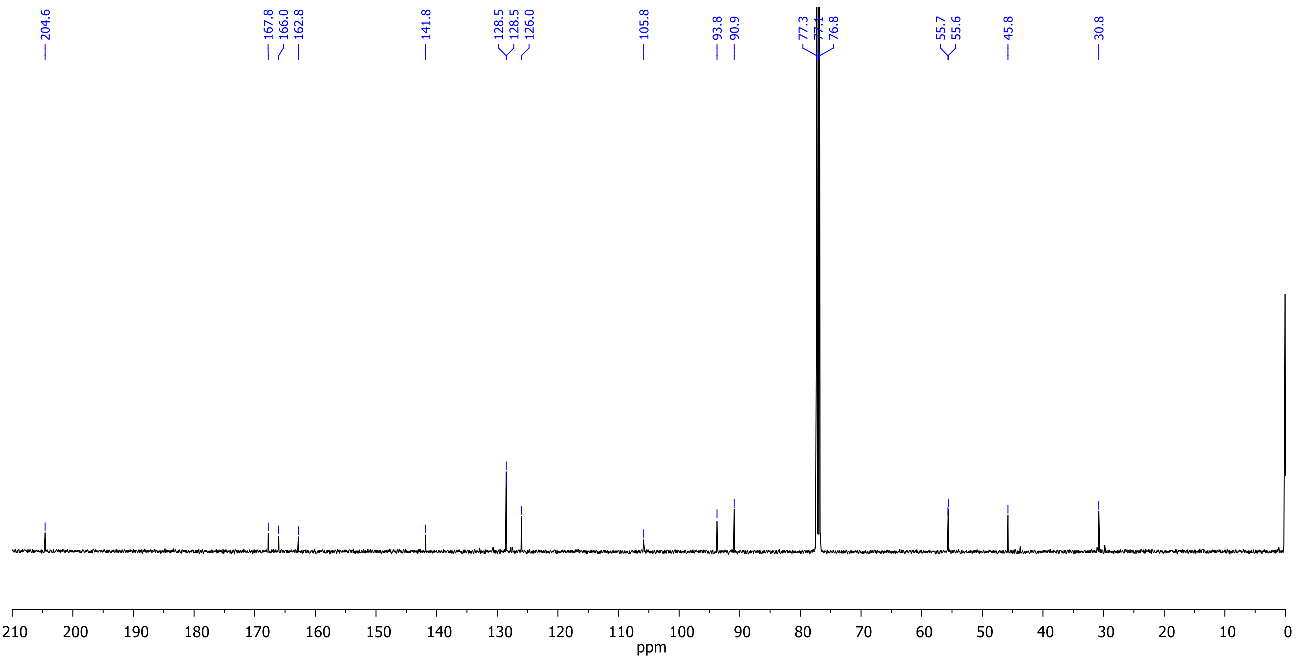

3-(3-bromophenyl)-1-(2-hydroxy-4,6-dimethoxyphenyl)propan-1-one (18)

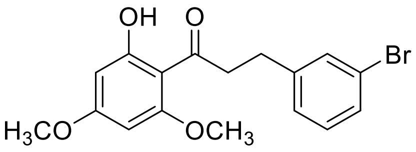

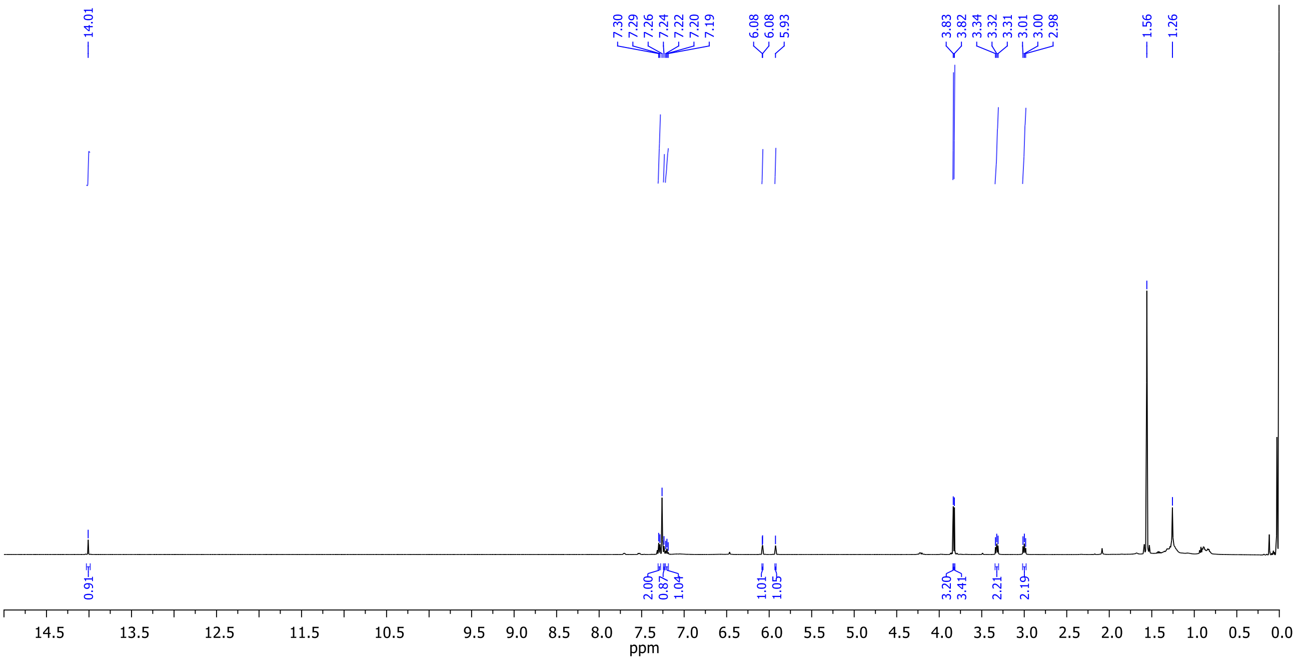

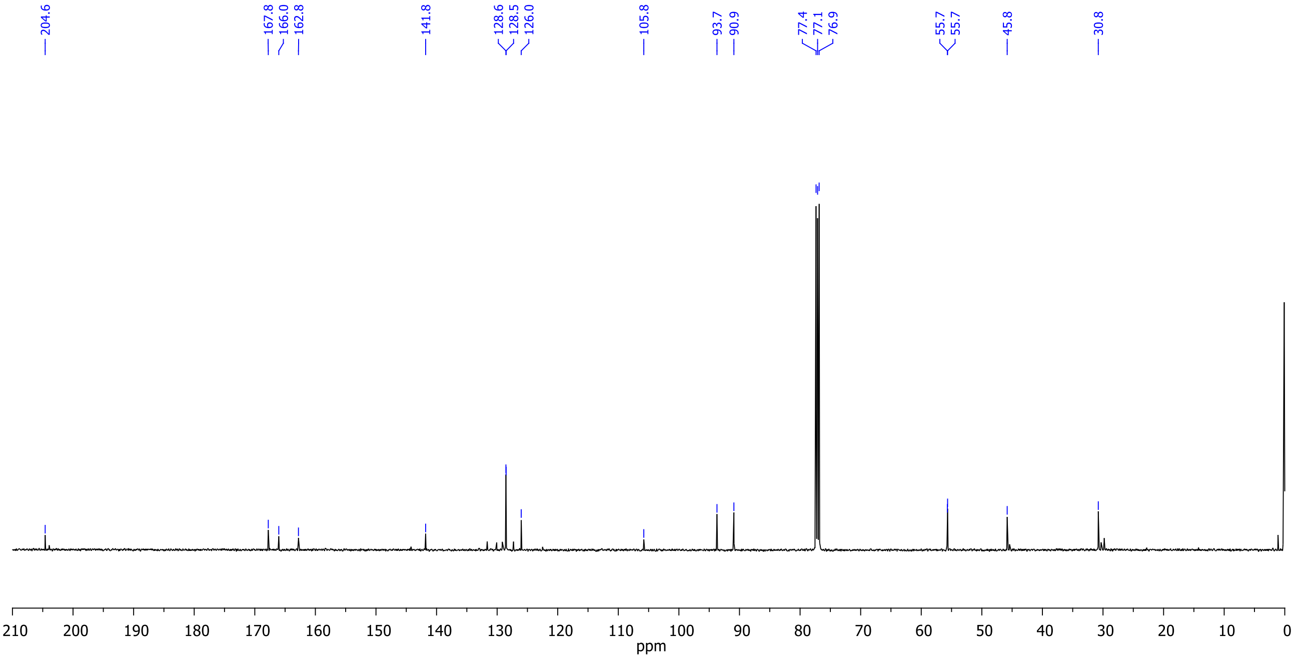

3-(4-bromophenyl)-1-(2-hydroxy-4,6-dimethoxyphenyl)propan-1-one (19)

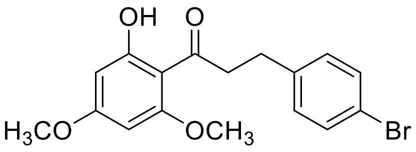

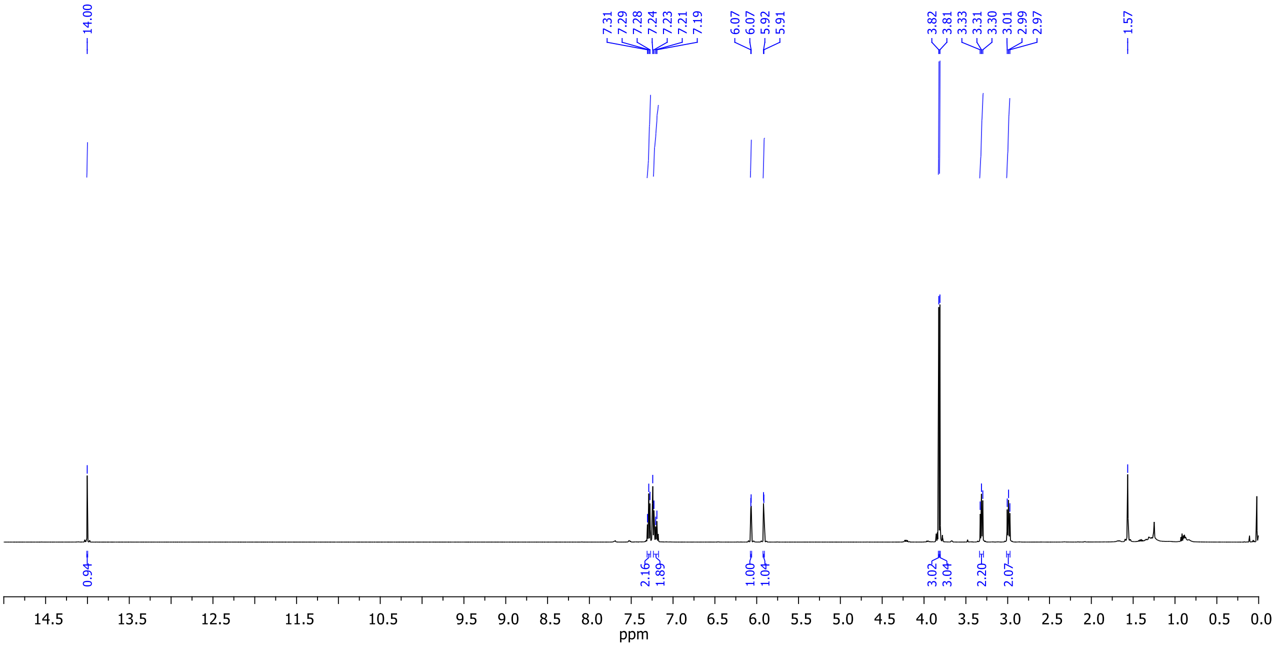

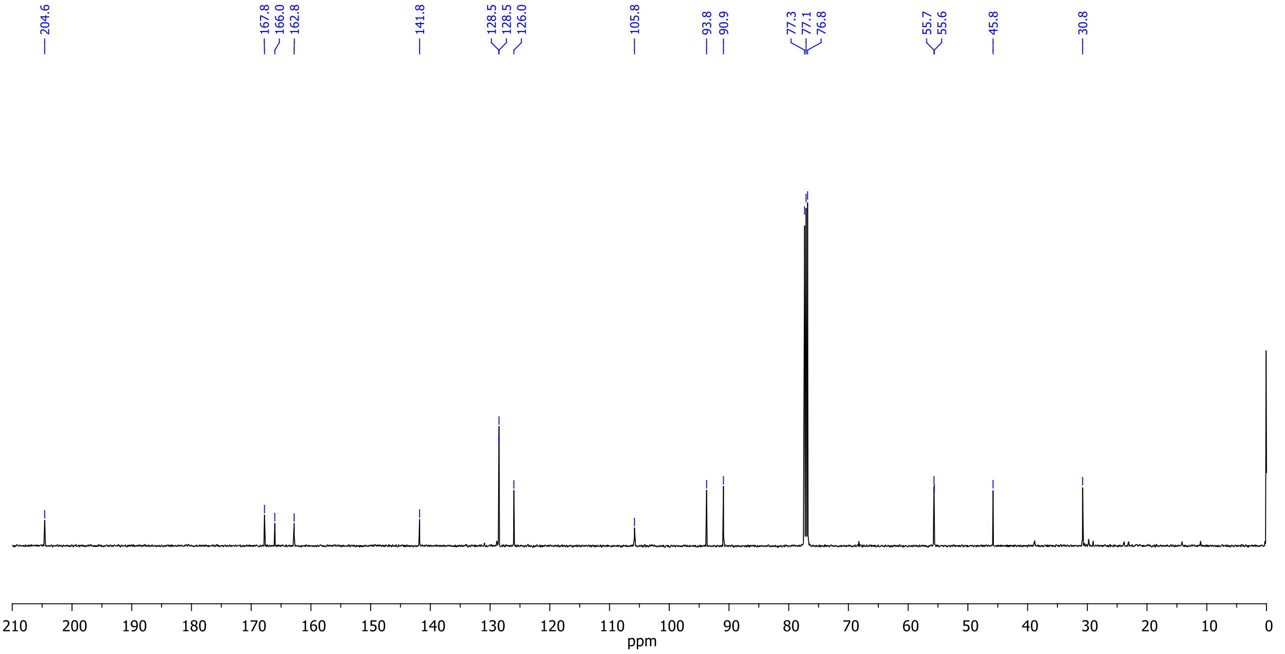

3-(3-(2-hydroxy-4,6-dimethoxyphenyl)-3-oxopropyl)benzoic acid (20)

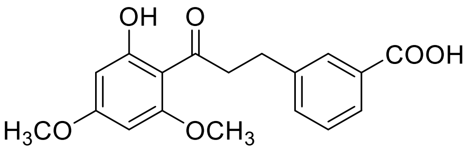

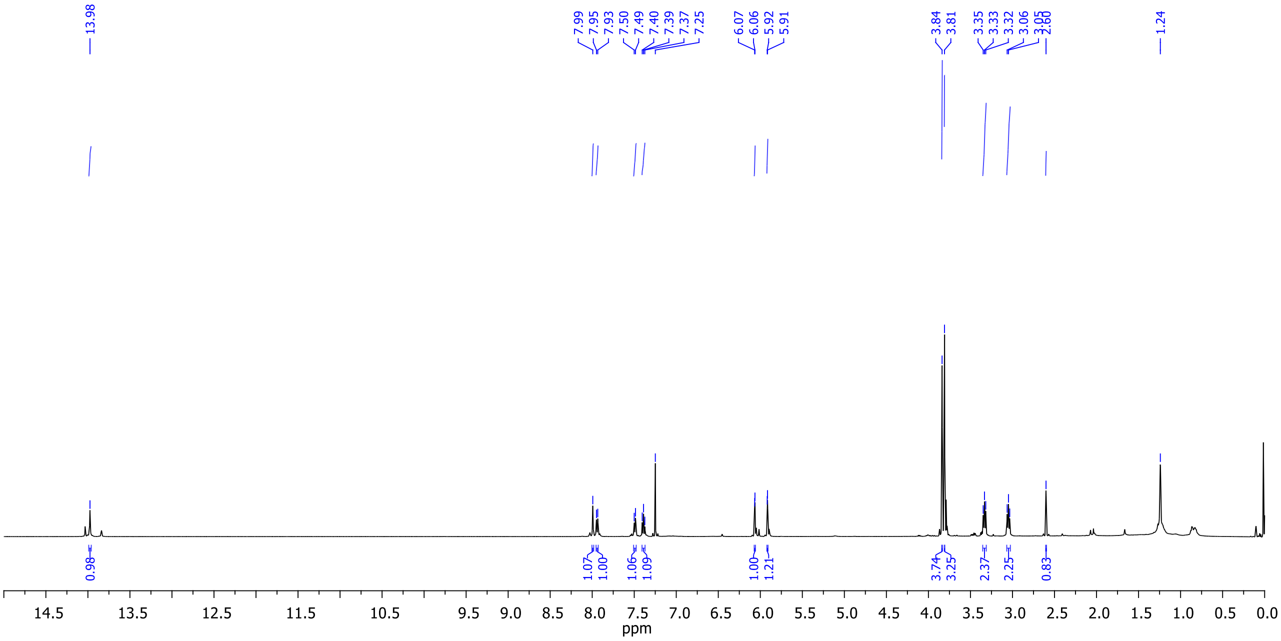

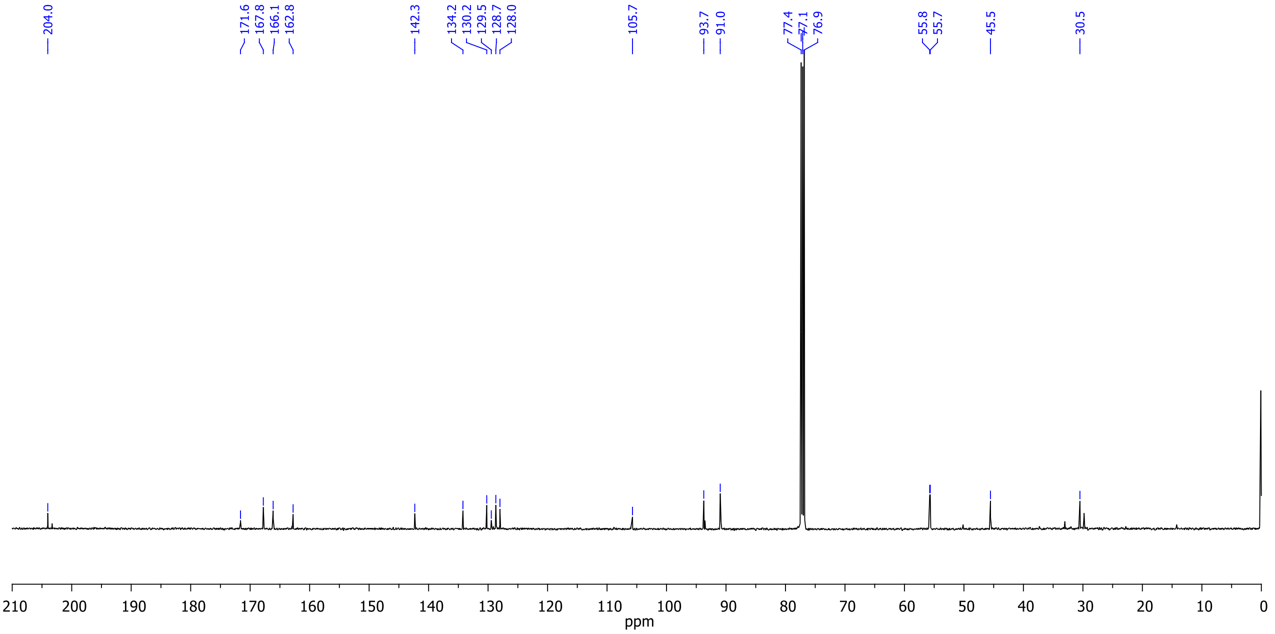

4-(3-(2-hydroxy-4,6-dimethoxyphenyl)-3-oxopropyl)benzoic acid (21)

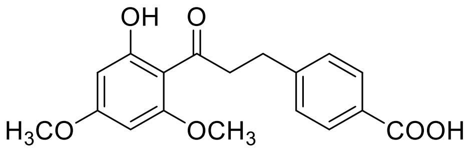

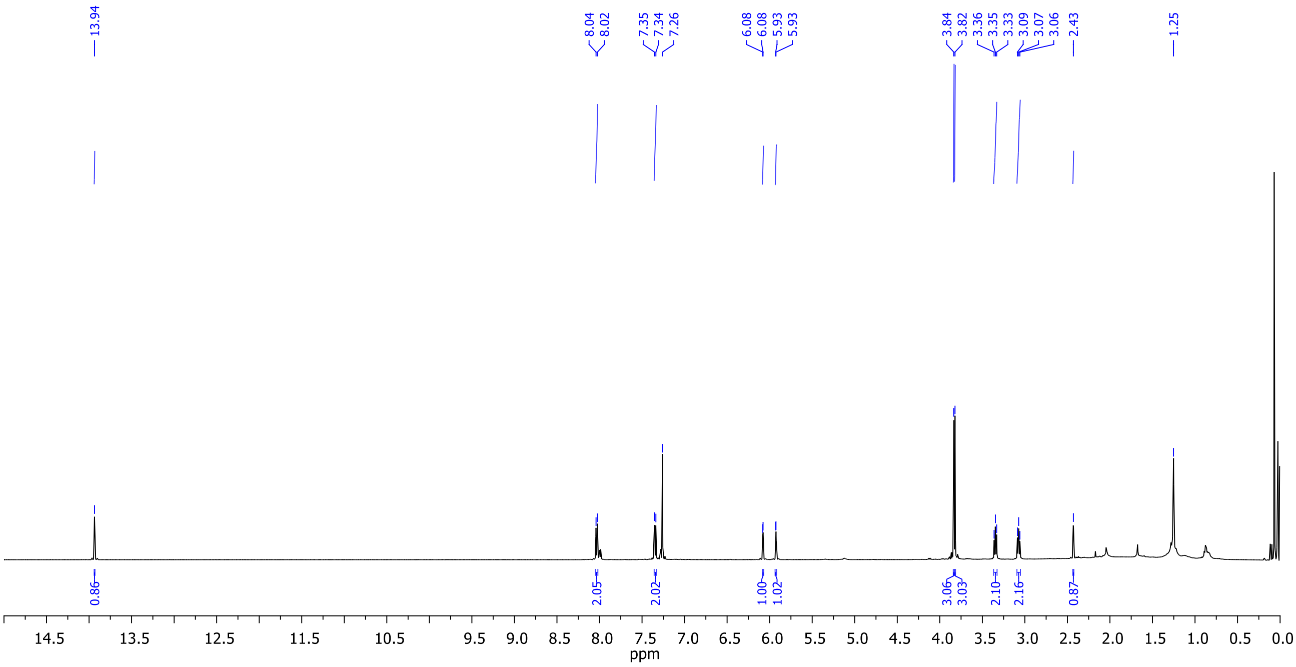

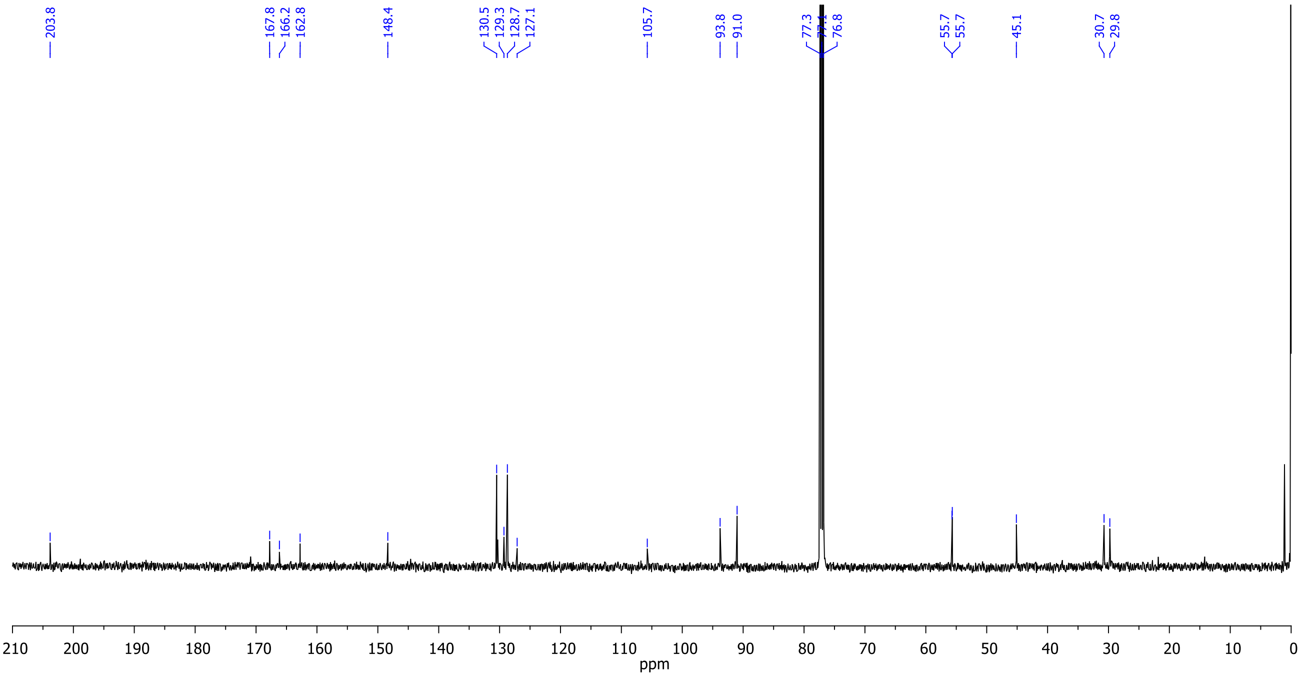

1-(2-hydroxy-4,6-dimethoxyphenyl)-3-(4-isopropoxyphenyl)propan-1-one (22)

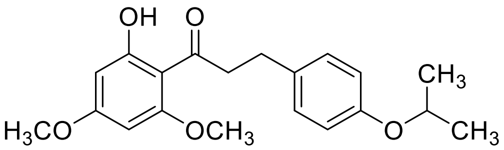

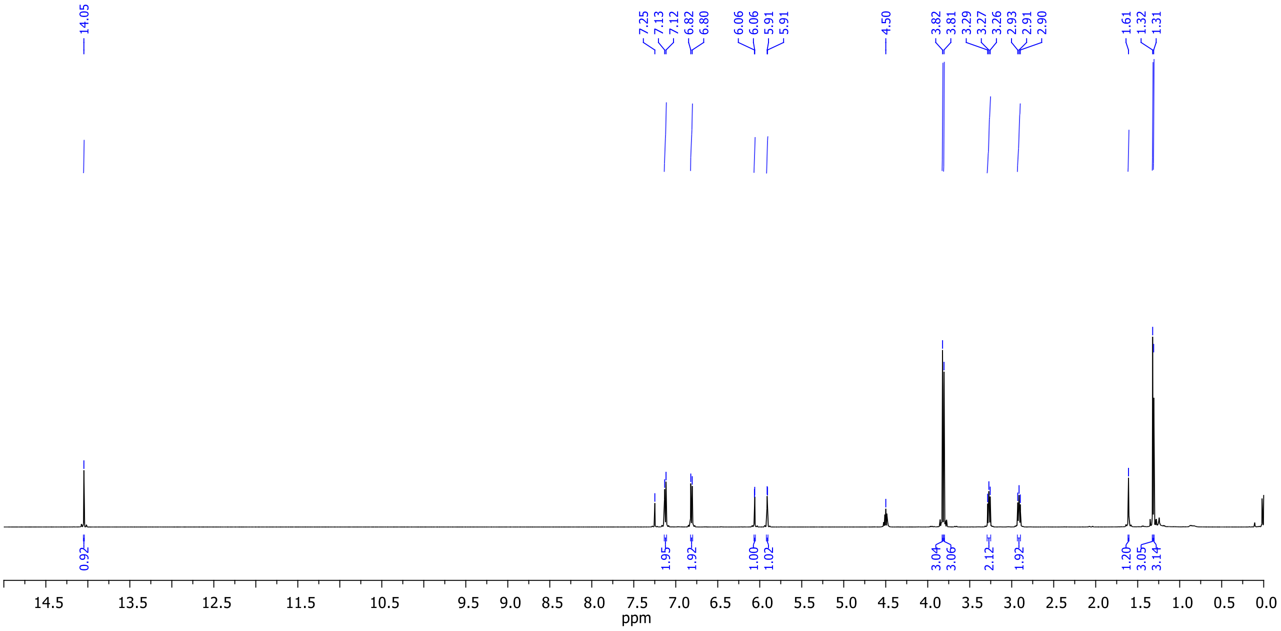

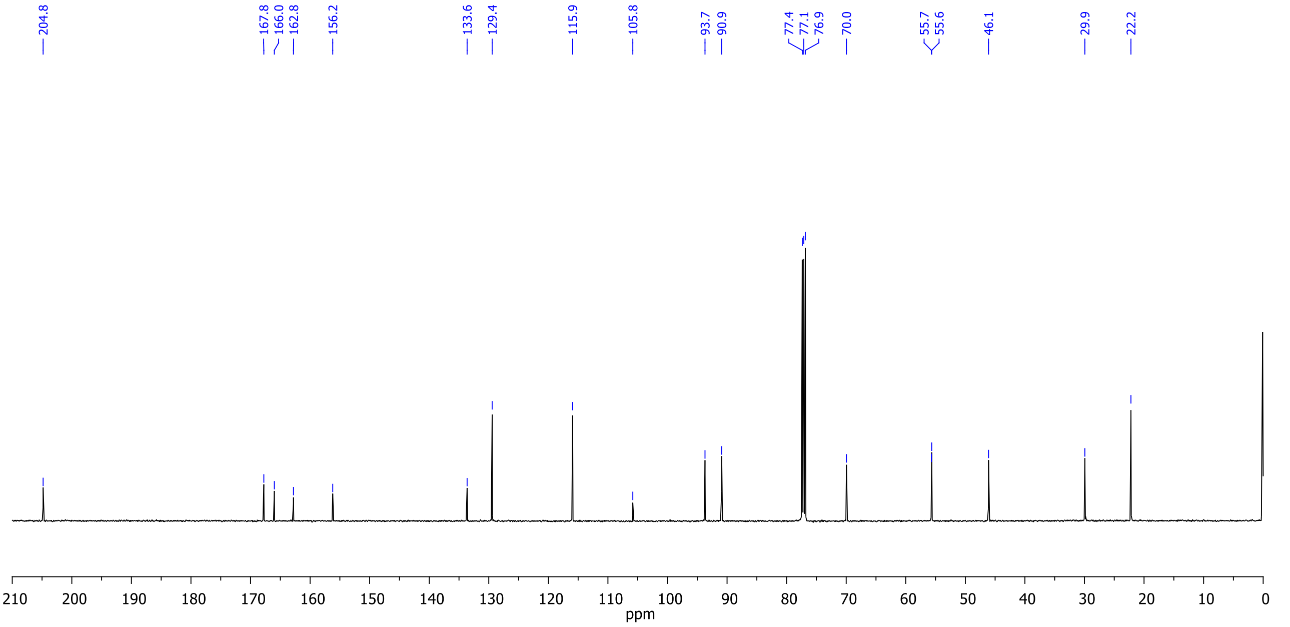

3-(4-(dimethylamino)phenyl)-1-(2-hydroxy-4,6-dimethoxyphenyl)propan-1-one (23)

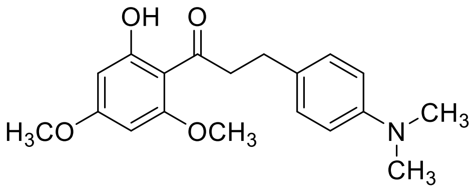

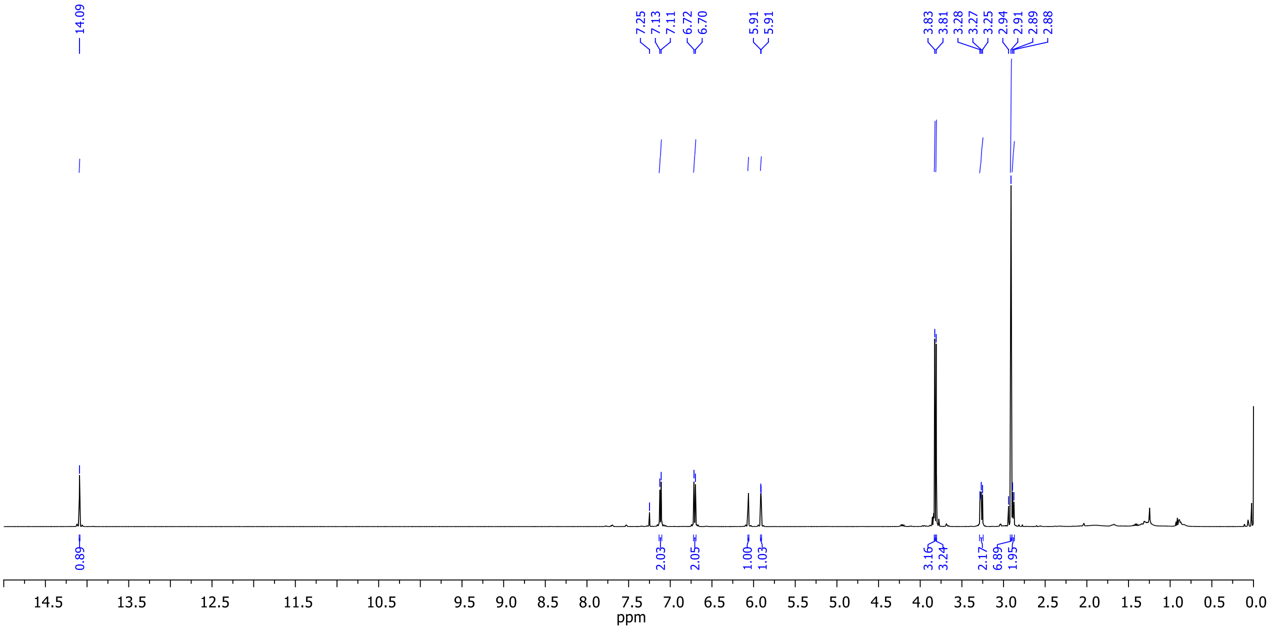

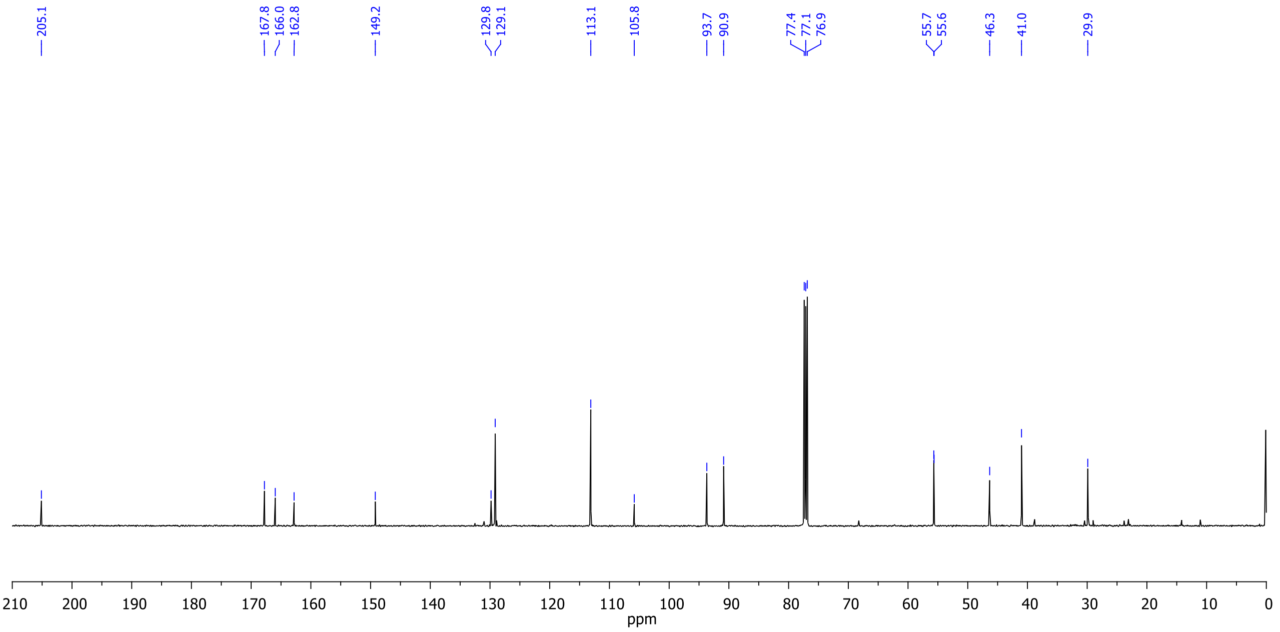

3-(4-hydroxy-3-methoxyphenyl)-1-(2-hydroxy-4,6-dimethoxyphenyl)propan-1-one (24)

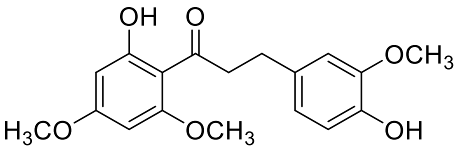

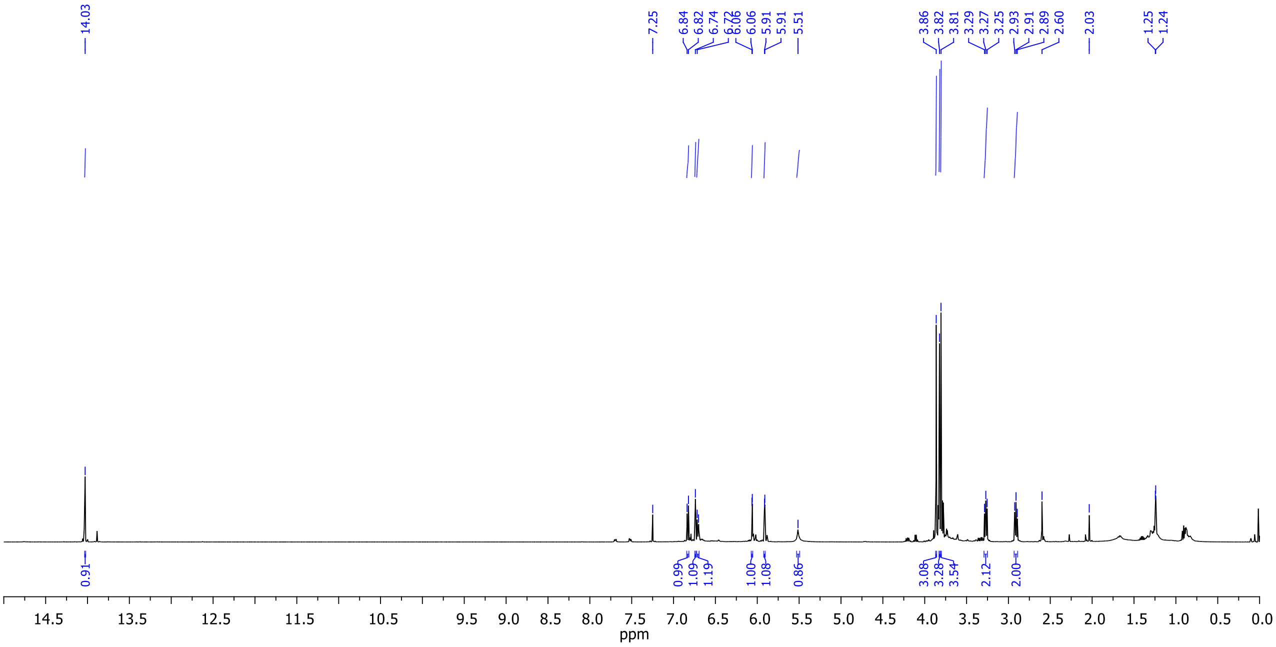

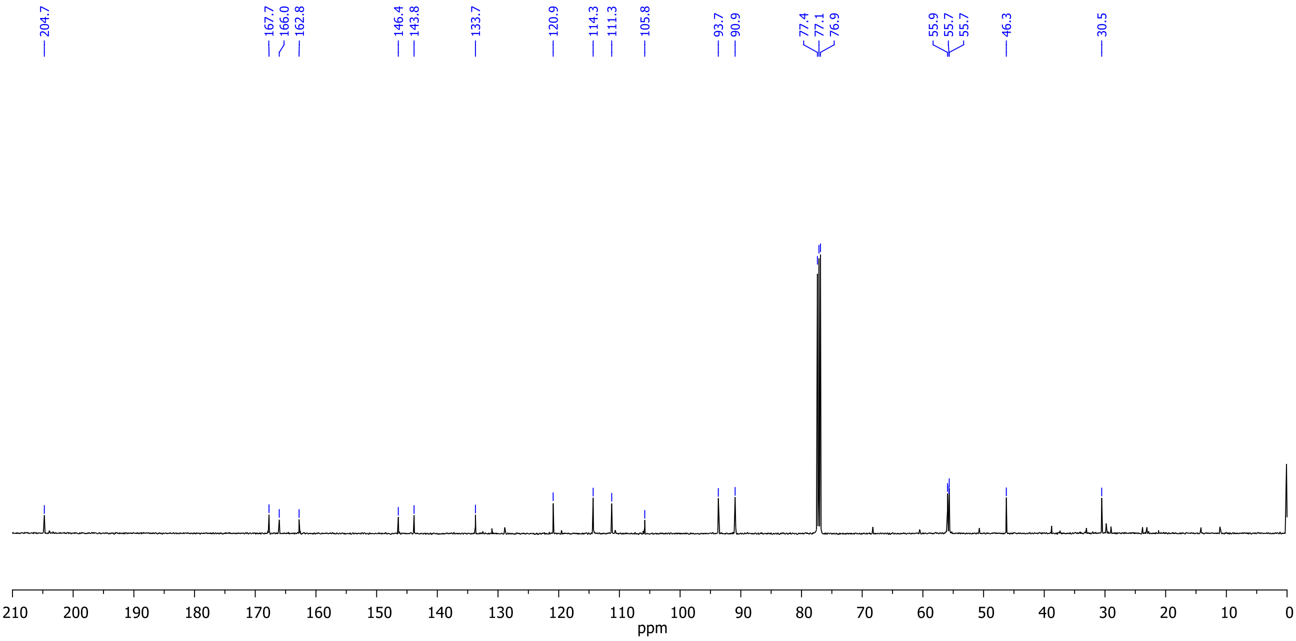

1-(2-hydroxy-4,6-dimethoxyphenyl)-3-(2,4,6-trimethoxyphenyl)propan-1-one (25)

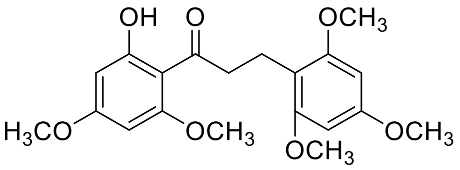

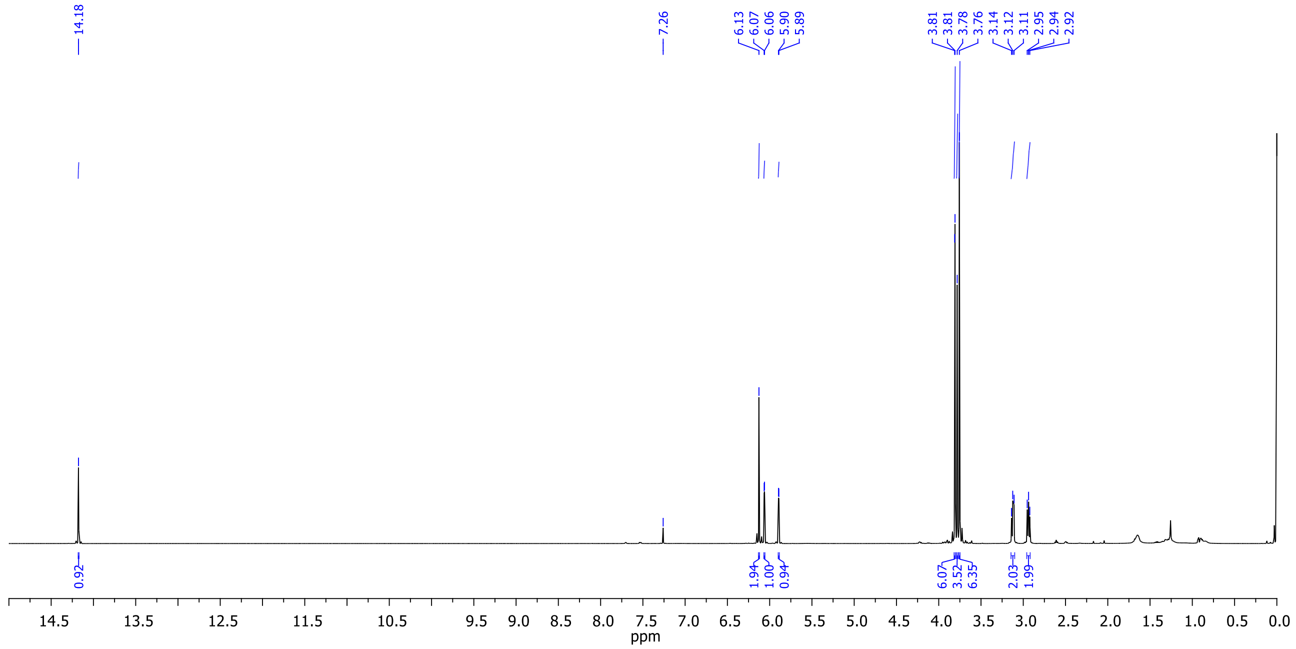

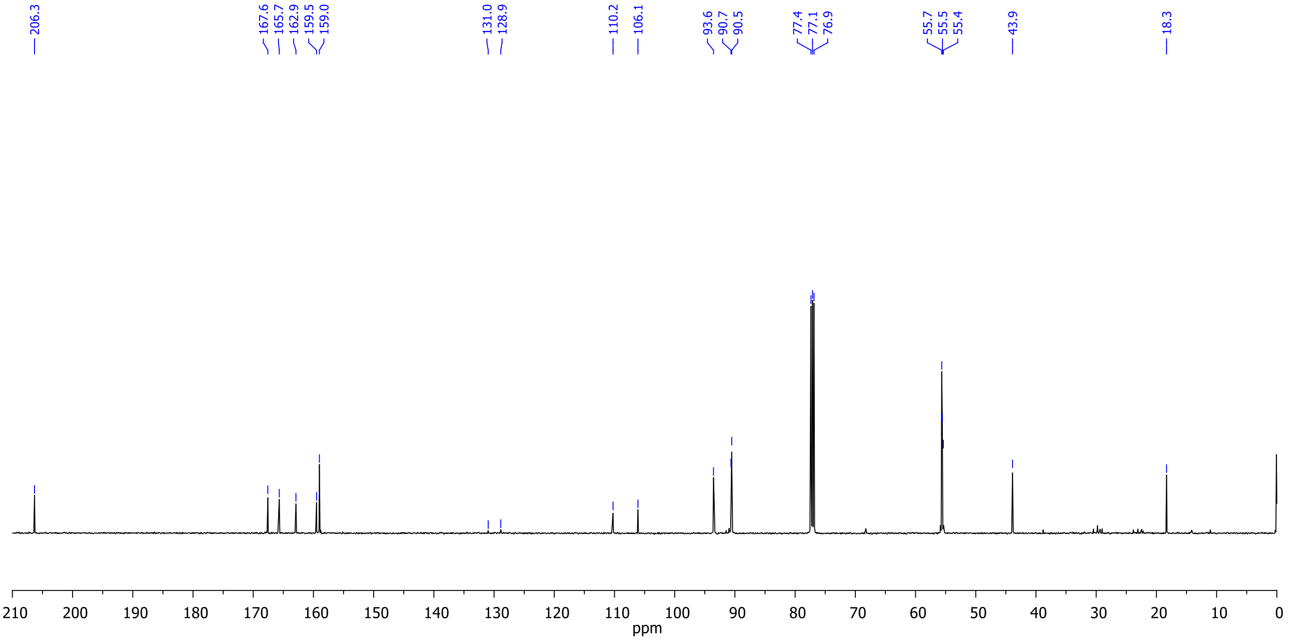

1-(2-hydroxy-4,6-dimethoxyphenyl)-3-(1-methyl-1H-pyrrol-2-yl)propan-1-one (26)

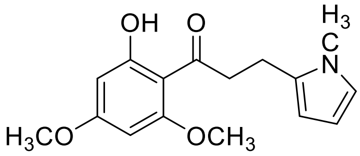

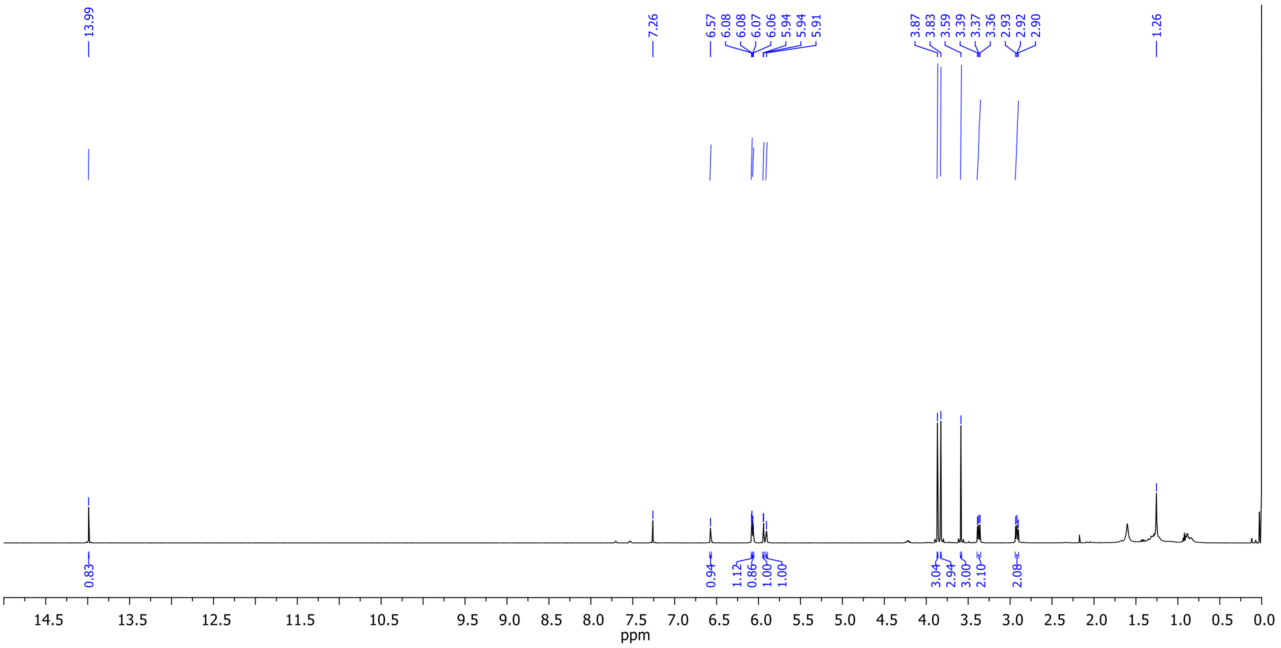

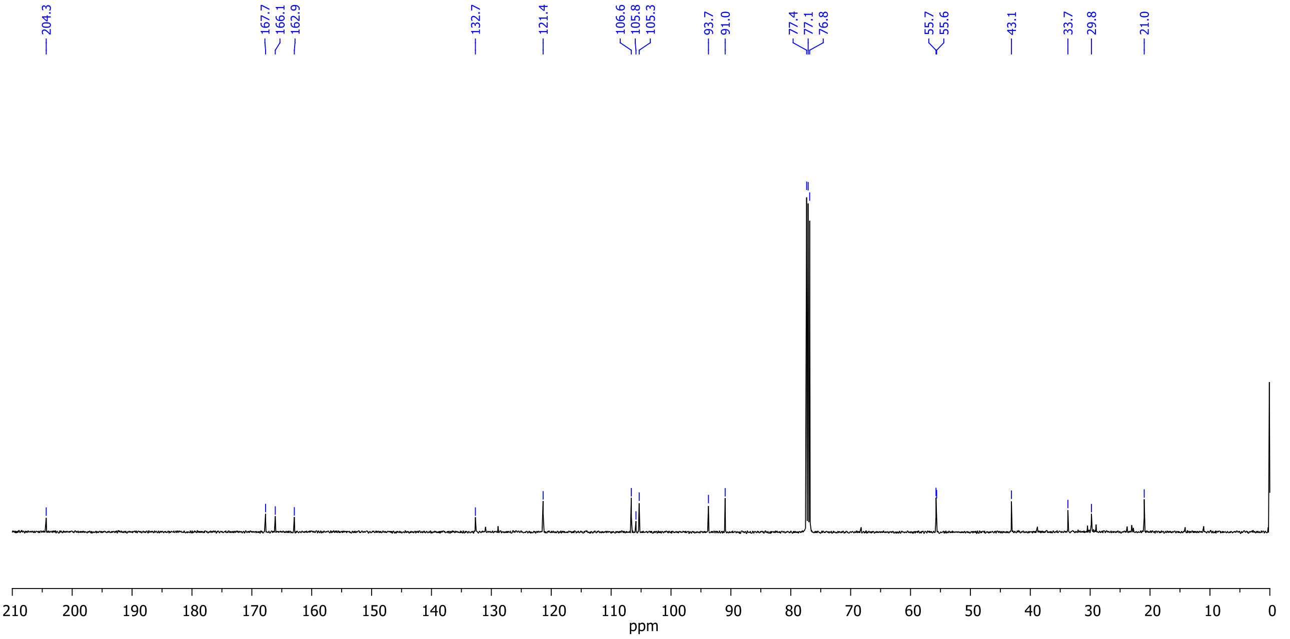

1-(2-hydroxy-4,6-dimethoxyphenyl)-3-(thiophen-2-yl)propan-1-one (27)

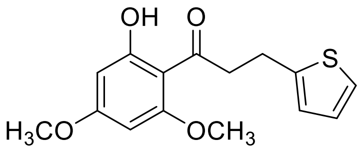

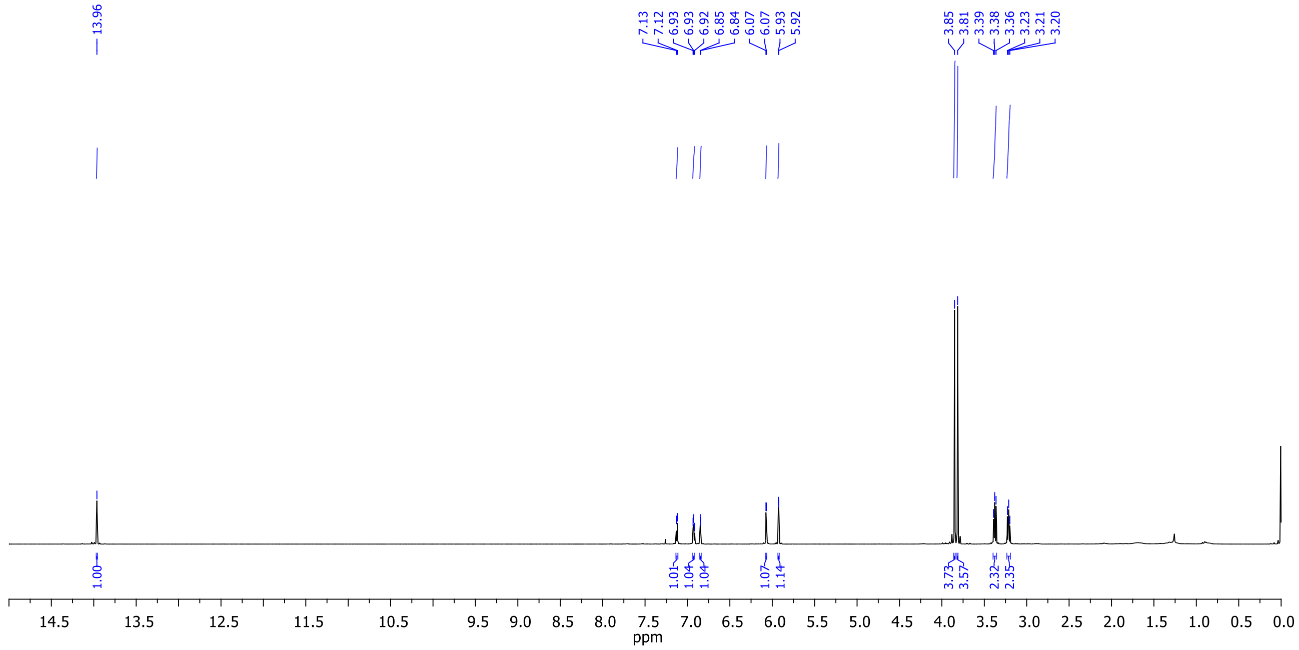

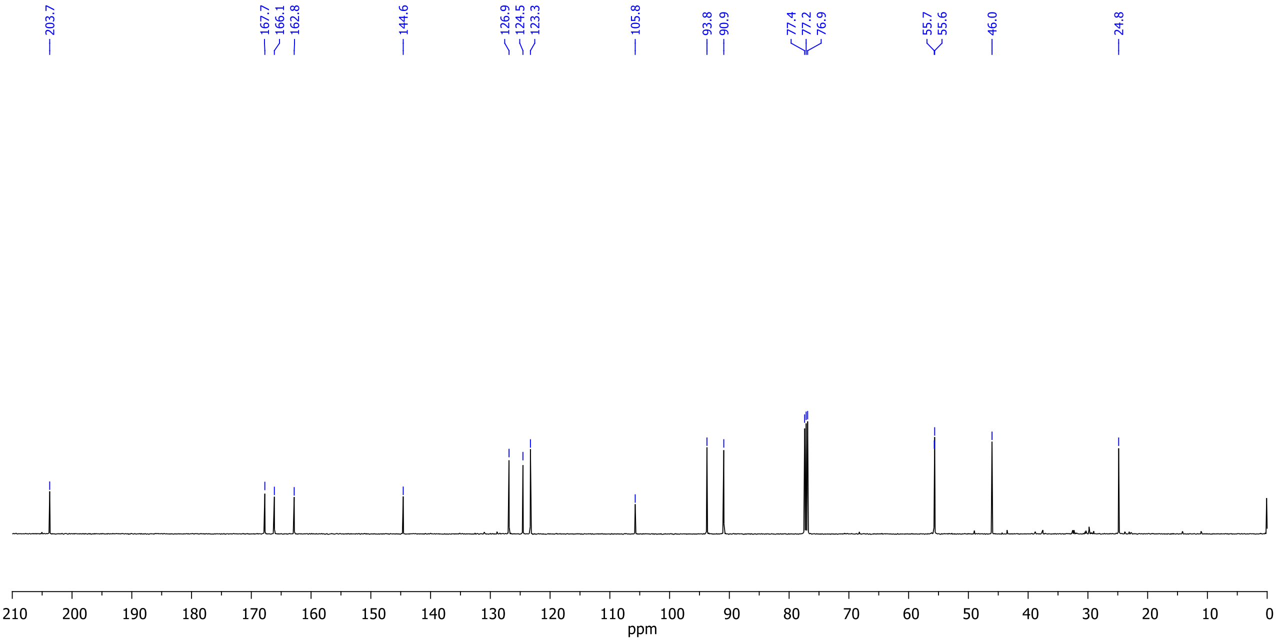

3) Reference

[1] Kostrzewa-Susłow, E.; Dymarska, M.; Guzik, U.; Wojcieszyńska, D.; Janeczko, T. *Stenotrophomonas maltophilia*: A Gram-Negative Bacterium Useful for Transformations of Flavanone and Chalcone. *Molecules* **2017**, *22*, 1830.
